# Discovery of Cymopolyphenols A–F From a Marine Mesophotic Zone *Aaptos* Sponge-Associated Fungus *Cymostachys* sp. NBUF082

**DOI:** 10.3389/fmicb.2021.638610

**Published:** 2021-02-22

**Authors:** Tingting Wang, Jing Zhou, Jiabin Zou, Yutong Shi, Wenli Zhou, Peng Shao, Tianze Yu, Wei Cui, Xiaohui Li, Xingxin Wu, Jing Ye, Xiaojun Yan, C. Benjamin Naman, J. Enrico H. Lazaro, Shan He

**Affiliations:** ^1^Li Dak Sum Marine Biopharmaceutical Research Center, Department of Marine Pharmacy, College of Food and Pharmaceutical Sciences, Ningbo University, Ningbo, China; ^2^College of Fisheries, Tianjin Agricultural University, Tianjin, China; ^3^Zhejiang Provincial Key Laboratory of Pathophysiology, School of Medicine, Ningbo University, Ningbo, China; ^4^State Key Laboratory of Pharmaceutical Biotechnology, School of Life Sciences, Nanjing University, Nanjing, China; ^5^National Institute of Molecular Biology and Biotechnology, University of the Philippines Diliman, Quezon, Philippines

**Keywords:** mesophotic coral ecosystems, twilight zone, sponges, fungi, sponge-associated fungi, natural products, polyphenols, dihydroisobenzofuran

## Abstract

Mesophotic coral ecosystems (MCEs) have complex but understudied biodiversity, especially for natural products discovery. Untargeted metabolomics research on 80 extracts prepared from marine sponge-associated fungi, half from shallow reefs (<30 m) and half from MCEs (30–150 m), facilitated prioritization for further study a *Cymostachys* fungus from a 103 m deep *Aaptos* sponge. LC-MS target-directed isolation yielded a series of new compounds, cymopolyphenols A−F (**1**–**6**), and two known phenylspirodrimanes, F1839-I (**7**) and stachybotrylactone (**8**). This is the first report of natural products from the recently described genus, *Cymostachys*. Compounds **1**–**6** and **8** contain a dihydroisobenzofuran moiety, and **4**–**6** are low-order polymers of **1** with novel scaffolds. The structures of the compounds were established by spectroscopic and spectrometric data interpretation, with further support from X-ray crystallography studies of **3** and **4**. Compound **3** undergoes facile racemization in solution and was found to crystalize as a racemic mixture. Compound **5** was also obtained in racemic form, and after chiral chromatography, both separated enantiomers racemized in solution by a presumed keto-enol tautomerization. Compounds **1** and **3**–**6** were found to be weakly antimicrobial (MIC 16–64 μg/ml) *in vitro* against several Gram-positive and Gram-negative human or aquatic pathogens, compound **5** was shown to chelate iron *in vitro* at 10 μM, and **8** activated plant disease resistance *in vivo* in a transgenic model organism.

## Introduction

Natural products research has long been instrumental in generating lead molecules for drug discovery, and many natural products have reached the clinic without structural modification by medicinal chemistry (Cragg et al., 2009; Lachance et al., 2012; Agarwal et al., 2020; Newman and Cragg, 2020). For decades, scientists have followed the adage that studying biodiversity leads to chemodiversity, and the continued exploration of environmental niches and different branches of life has been fruitful (Suffness and Douros, 1981). Studies of marine organisms were at one point considered to be pioneering, and now thousands of marine natural products have been reported. These have provided a resource for drug development and, “as shown at the global marine pharmaceutical pipeline website,^1^ there are currently nine approved marine-derived pharmaceuticals, and an additional 31 compounds are either in Phase I, II, and III of *clinical* pharmaceutical development” (Mayer et al., 2020).

In the ocean, mesophotic coral ecosystems (MCEs, also known as twilight zone reefs) that range from 30 to 150 m deep represent an understudied environmental frontier for the collection of sponges and other macroscopic organisms that harbor diverse microbes (Olson and Kellogg, 2010; Weiss, 2017). It was reported that MCEs represent approximately 80% of coral reef habitat worldwide, yet very little is known about these deep habitats comparing to shallow reefs (Pyle and Copus, 2019). The biodiversity found in MCEs appears to differ significantly from that of shallow reefs, but a small fraction of life present there has been categorized taxonomically or examined in natural products research due to technical challenges (Sinniger et al., 2016; Lesser et al., 2018; Rocha et al., 2018). One strategy to access this resource is the investigation of assemblages produced from dredging, but this practice is extremely damaging to the environment (Machida et al., 2014; Pedrosa et al., 2020). Because most everything comes up broken into pieces, it is also challenging to determine the producing organism of any molecules discovered, e.g., by analytical comparison with extracts of sorted and identified biomass fragments (Machida et al., 2014; Pedrosa et al., 2020). More natural product studies have thus been reported on mesophotic zone organisms after being collected by a remotely operated vehicle (ROV) or autonomous underwater vehicle (AUV). However, this practice is very costly and usually reserved for studying the much deeper bathypelagic and abyssopelagic zones, or even hadopelagic trenches (Schupp et al., 2009). Scientific SCUBA diving to the mesophotic zone is preferable, but carries many challenges including the need for mixed gases, multiple tanks, and/or rebreather apparatus, and relatively few divers are trained for such depths. There are additionally dramatic safety limitations, including short working time at depth, long decompression stops to return to the surface, and risks of nitrogen narcosis and oxygen toxicity. Still, technical scientific diving is more eco-friendly than dredging and more cost-effective and efficient than using an ROV or AUV for sample collection.

A previous preliminary survey of mesophotic zone organisms from Guam reported that “extracts from the twilight zone sponges and gorgonians resulted in an astonishing 72% hit rate” using *in vitro* cancer chemopreventive and antiproliferative bioassays and already yielded some new natural products (Schupp et al., 2009; Wright et al., 2012). While many researchers have studied macroscopic marine organisms, some natural products they discovered have been suspected or shown to be produced by associated microorganisms (Hirata and Uemura, 1986; Still et al., 2014; McCauley et al., 2020; Newman and Cragg, 2020). Recently, the multinational EU-funded program, TASCMAR (Tools And Strategies to access original bioactive compounds by Cultivating MARine invertebrates and associated symbionts^2^), has begun to harness of the microbial diversity of some mesophotic zone invertebrates for the purpose of natural products discovery projects, and these have reported new bioactive molecules from interesting microbes (Le Goff et al., 2019; Nikolaivits et al., 2019; Letsiou et al., 2020).

Fungal metabolism is well-characterized as being diverse, and often leads to natural products with useful pharmacological activities, but may require efforts to activate in laboratory cultures as exemplified by the “One Strain, MAny Compounds” (OSMAC) approach (Keller, 2019). It is now typical to cultivate in a laboratory the microbes found associated with sponges, especially fungi, and then reproducibly access the biosynthetic potential of these organisms under different conditions (Li and Wang, 2009; Zhang et al., 2020). Meanwhile, it is understood that mesophotic zone organisms represent a vastly unexplored biological diversity with great potential for natural products drug discovery. In the current study, sample prioritization was achieved by the OSMAC strategy combined with LC-MS/MS molecular networking (Wang et al., 2016) of organic extracts produced from 40 cultured fungi isolated from mesophotic zone sponges and 40 more from shallow reef sponges collected by scientific diving. This allowed a *Cymostachys* fungus to be selected for further study based on the observed production of then-hypothesized and now-demonstrated new natural products of interest.

**Figure 1:**
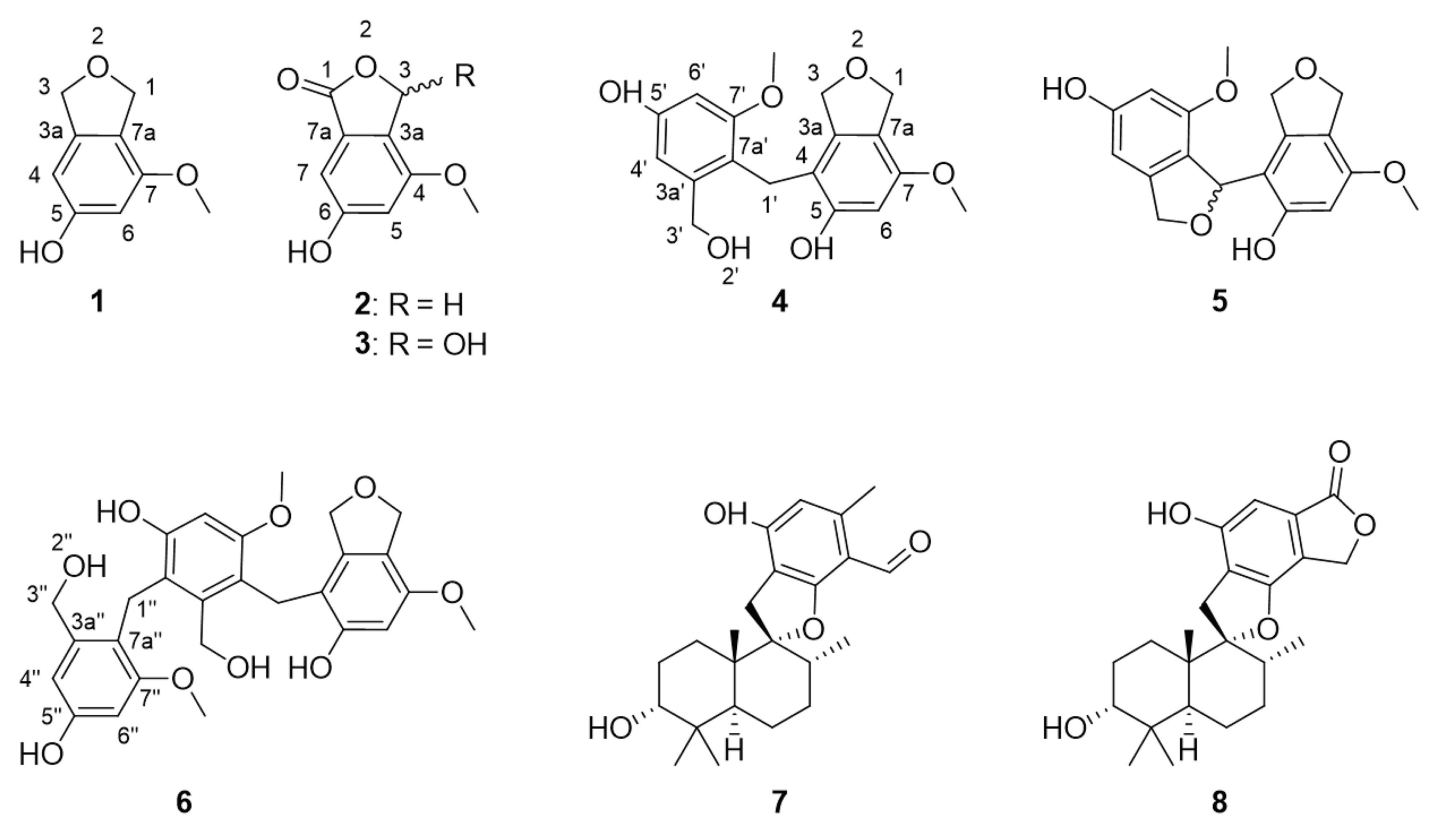
Structures of compounds 1–8, here discovered from *Cymostachys* sp. NBUF082. Compounds 7 and 8 were previously reported from *Stachybotrys* fungi.

The genus *Cymostachys* was only recently described, and no earlier literature exists on the natural products chemistry of any species therein (Lombard et al., 2016). *Cymostachys* is closely related to the genus *Stachybotrys*, and the latter has been extensively studied for its natural product chemistry (Wang et al., 2015; Zhao et al., 2017; Jagels et al., 2019; Zheng et al., 2019). For example, compounds **7** and **8** (Figure 1) were both originally discovered from *Stachybotrys* fungi (Ayer and Miao, 1993; Sakai et al., 1995; Zhao et al., 2017). The organism prioritization, targeted compound isolation and characterization of new molecules for ongoing natural product drug discovery efforts are described herein.

## Materials and Methods

### General Experimental Procedures

Optical rotations were acquired on a JASCO P-2000 automatic polarimeter in MeOH at 20°C. NMR spectra were recorded on a Bruker AVANCE NEO 600 spectrometer with a 5 mm inverse detection triple resonance (H-C/N/D) cryoprobe having z-gradients, and Bruker AVANCE 500 spectrometer with a 5 mm double resonance broadband room temperature probe. Spectra were collected using standard Bruker pulse programs, and chemical shifts were recorded relative to the solvent peak in DMSO-*d*_6_ (*δ*_H_ 2.50 and *δ*_C_ 39.52). High-resolution electrospray ionization mass spectra (HRESIMS) were measured on an Agilent (Santa Clara, CA, United States) 6545 Q-TOF instrument. Reversed-phase HPLC purification was performed using a Waters HPLC equipped with a 1525 binary pump, and a Thermo Scientific (Waltham, MA, United States) ODS-2 Hypersil column (5 μm, 250 mm × 10 mm). Analytical chiral HPLC was performed using a Waters HPLC equipped with a 1525 binary pump and a Sepax Technologies (Newark, Delaware, United States) Chiralomix SA column (5 μm, 250 mm × 4.6 mm). Normal phase column chromatography and thin-layer chromatography were accomplished using coarse (200–300 mesh) and fine GF254 (10–20 μm) silica, respectively (Qingdao Marine Chemical Company, China). Sephadex LH-20 (Pharmacia Biotech, Sweden) was used for gel filtration chromatography, and YMC*GEL ODS-A (AA12S50; YMC Co., Ltd., Japan) was used for reverse phase column chromatography. Biological assays were read for absorbance determination on a Thermo Scientific Multiskan GO microplate spectrophotometer.

### Organism Collection and Identification

The fungi evaluated in this study were isolated from sponges collected in a shallow water reef and the deeper MCEs near Apo Island, Negros Oriental, Philippines (9°04'40.6''N 123°15'57.3''E and 9°04'33.0''N 123°15'59.1''E) by scientific technical SCUBA diving in October 2018. The details of specific sponge identification and sampling depths are listed in Supplementary Table S1. The fresh inner tissue of the each sponge was sliced and stuck on petri dishes containing modified Czapek’s medium (sucrose 3.0 g, Na_2_NO_3_ 3.0 g, MgSO_4_·7H_2_O 0.5 g, FeSO_4_·7H_2_O 0.001 g, KH_2_PO_4_ 1.0 g, KCl 0.5 g, yeast extract 1.0 g, kanamycin 150 mg, ampicillin sodium 150 mg, sea salt 35.0 g, agar powder 20.0 g, and H_2_O up to a total volume of 1 L), modified potato dextrose agar (modified PDA: potato 20.0 g, glucose 2.0 g, kanamycin 150 mg, ampicillin sodium 150 mg, sea salt 35.0 g, agar powder 20.0 g, and H_2_O up to a total volume of 1 L), and modified martin medium (peptone 10.0 g, yeast extract 20.0 g, sucrose 1.0 g, KH_2_PO_4_ 1.0 g, MgSO_4_ 0.5 g, kanamycin 150 mg, ampicillin sodium 150 mg, sea salt 35.0 g, agar powder 20.0 g, and H_2_O up to a total volume of 1 L). Two weeks later, fungal colonies on the plates were picked and purified on petri dishes containing PDA (glucose 20.0 g, potato 200 g, sea salt 35.0 g, agar powder 20.0 g, and H_2_O up to a total volume of 1 L). Voucher specimens were deposited at the College of Food and Pharmaceutical Sciences, Ningbo University, Ningbo, China, available from SH.

The fungal strain studied most extensively in this work, NBUF082, was inoculated at three points on PDA and cultivated at 28°C for 5 days. The fungal colonies were fast-growing and flocculated, and they turned from white to light brown color on cultivation day 5. The reverse side of the medium was fawn-colored and non-extravasated. The strain was able to be identified as belonging to the genus *Cymostachys* according to its morphological traits (Lin et al., 2016), and sequence analysis of the ITS region (GenBank accession no. MW077215) as described previously (Henríquez et al., 2014; Lombard et al., 2016). Two other fungi were isolated from the same *Aaptos* sponge in addition to *Cymostachys* sp. NBUF082. These were identified as belonging to genera *Roussoella* and *Aspergillus*, respectively, based on morphological traits and sequence analysis of the ITS region (Henríquez et al., 2014).

### Small-Scale Cultivation, Extraction, and Molecular Networking

Inspired by the OSMAC strategy, the obtained sponge-derived fungi were each cultured separately in three different types of media, potato dextrose broth (PDB: glucose 20.0 g, potato 200.0 g, sea salt 35.0 g, and H_2_O up to a total volume of 1 L), Czapek-Dox medium (sucrose 30.0 g, Na_2_NO_3_ 3.0 g, MgSO_4_·7H_2_O 0.5 g, FeSO_4_·7H_2_O 0.001 g, KH_2_PO_4_ 1.0 g, KCl 0.5 g, yeast extract 1.0 g, sea salt 35.0 g, and H_2_O up to a total volume of 1 L), and modified Martin medium (peptone 10.0 g, yeast extract 20.0 g, sucrose 10.0 g, KH_2_PO_4_ 1.0 g, MgSO_4_ 0.5 g, sea salt 35.0 g, and H_2_O up to a total volume of 1 L). The fungal mycelia on petri-dishes were cut into squares (0.5 cm^3^ × 0.5 cm^3^ × 0.5 cm^3^) and incubated into 1 L Erlenmeyer flasks containing 400 ml of above-mentioned medium. The cultures were incubated for 15 days at 28°C with agitation (120 rpm), and then extracted with EtOAc (v/v, 1:1) three times each. The crude extracts were concentrated under vacuum with rotary evaporators.

The extracts were dissolved in MeOH to final concentrations of 1 mg/ml and preprocessed by 0.22 μm membrane filtration. A 3 μl aliquot of each sample was injected into the LC-HRESIMS and eluted at 0.8 ml/min (MeOH/H_2_O with 0.1% formic acid, v/v, 30%→99%): 30% for 5 min to 99% in 17 min, held for 3 min, to 30% in 1 min, and held for 4 min. The mass spectrometer was set to observe m/z 190–2000 in positive ESI mode and with an automated data-dependent MS/MS scan enabled. The resulting data were uploaded to the Global Natural Product Social Molecular Networking web interface (GNPS^3^), and the results were used to generate molecular network diagrams using the freely available open source visualization software, Cytoscape.^4^

### Large-Scale Fermentation, Extraction, and Isolation

The *Cymostachys* fungus of interest was scaled up in culture size for chemical investigation. First, it was cultivated on potato dextrose agar (PDA) at 28°C for 7 days. The mycelia on PDA in petri dishes were cut into squares (0.5 cm^3^ × 0.5 cm^3^ × 0.5 cm^3^) and incubated into 280 L × 1 L Erlenmeyer flasks, each containing 400 ml PDB medium (80 g potato dextrose, 8 g glucose, 14.0 g sea salt, and 400 ml H_2_O). The cultures were incubated for 15 days at 28°C with agitation (120 rpm) and then extracted with EtOAc (v/v, 1:1) for three times.

The combined organic phase was concentrated under reduced pressure to give 350 g (partially wet weight) of crude extract, which was subjected to column chromatography (CC) over silica gel (PE/EtOAc, v/v, 100:0→0:100 then EtOAc/MeOH v/v, 100:0→0:100) to give 10 fractions (Fr.1–Fr.10). Of these, Fr.5, which eluted from the column with 1:1 PE/EtOAc, v/v, was separated with Sephadex LH-20 in MeOH to yield 15 subfractions (Fr.5.1–Fr.5.15). Fr.5.13 was further purified by RP-HPLC with CH_3_CN/H_2_O (45:55, 2 ml/min) to afford compounds **4** (*t*_R_ = 48 min, 11.3 mg), **5** (*t*_R_ = 42 min, 12.2 mg), and **6** (*t*_R_ = 57 min, 5.7 mg). Crude Fr.6, which eluted from the column with EtOAc, was chromatographed again with silica gel (PE/EtOAc, v/v, 100:0→0:100 then EtOAc/MeOH v/v, 100:0→0:100) to afford 10 subfractions (Fr.6.1–Fr.6.10). Fr.6.6 and Fr.6.7, which both eluted with EtOAc, were subjected to RP-HPLC to yield compounds **7** (*t*_R_ = 30 min, 3.4 mg) and **8** (*t*_R_ = 39 min, 14.6 mg) by elution with CH_3_CN/H_2_O (42:58, 2 ml/min) and CH_3_CN/H_2_O (45:55, 2 ml/min), respectively. Fr. 6.8, which was eluted from the column by 40:1 EtOAc/MeOH, v/v, was separated with Sephadex LH-20 in MeOH to yield 15 subfractions (Fr.6.8.1–Fr.6.8.15). Further purification of Fr.6.8.8 *via* RP-HPLC (CH_3_CN/H_2_O, 36:64, 2 ml/min) afforded compounds **1** (*t*_R_ = 28 min, 10.2 mg) and **2** (*t*_R_ = 30 min, 0.8 mg). Fr. 6.9, which was eluted from the column by 20:1 EtOAc/MeOH, v/v, was separated with Sephadex LH-20 in MeOH to yield fifteen subfractions (Fr.6.9.1–Fr.6.9.15). Subfraction Fr.6.9.5 yielded **3** (*t*_R_ = 25 min, 10.0 mg) after being subjected to RP-HPLC (CH_3_CN/H_2_O, 50:50, 2 ml/min).

### Isolated Materials (New Natural Products)

Cymopolyphenol A (**1**): White powder; UV (MeOH) *λ*_max_ (log *ε*) = 280 (3.74) nm; for ^1^H NMR and ^13^C NMR data see Table 1; HR-ESI-MS [M + H]^+^ *m/z* 167.0706 (calcd. for C_9_H_11_O_3_, 167.0703).
Cymopolyphenol B (**2**): White powder; UV (MeOH) *λ*_max_ (log *ε*) = 285 (3.08) nm; for ^1^H NMR and ^13^C NMR data see Table 1; HR-ESI-MS [M + H]^+^ *m/z* 181.0500 (calcd. for C_9_H_9_O_4_, 181.0495).
Cymopolyphenol C (**3**): Colorless prisms; [*α*]25D 0 (*c* 0.1, MeOH); UV (MeOH) *λ*_max_ (log *ε*) = 285 (3.11) nm; for ^1^H NMR and ^13^C NMR data see Table 1; HR-ESI-MS [M + H]^+^ *m/z* 197.0453 (calcd. for C_9_H_9_O_5_, 197.0444).
Cymopolyphenol D (**4**): Colorless prisms; UV (MeOH) *λ*_max_ (log *ε*) = 290 (3.66); for ^1^H NMR and ^13^C NMR data see Table 2; HR-ESI-MS [M + Na]^+^ *m/z* 355.1159 (calcd. for C_18_H_20_O_6_Na, 355.1152).
Cymopolyphenol E (**5**): Light brown powder; [*α*]25D 0 (*c* 0.1, MeOH); UV (MeOH) *λ*_max_ (log *ε*) = 285 (3.82) nm; for ^1^H NMR and ^13^C NMR data see Table 2; HR-ESI-MS [M + Na]^+^ *m/z* 353.0997 (calcd. for C_18_H_18_O_6_Na, 353.0996).
Cymopolyphenol *F* (**6**): White powder; UV (MeOH) *λ*_max_ (log *ε*) = 285 (3.81) nm; for ^1^H NMR and ^13^C NMR data see Table 2; HR-ESI-MS [M + Na]^+^ *m/z* 521.1790 (calcd. for C_27_H_30_O_9_Na, 521.1782).

### Single Crystal X-ray Diffraction Analysis

The crystals obtained for **3** and **4** were evaluated on a Bruker APEX-II CCD diffractometer through Ga Kα (*λ* = 1.34139 Å). The structures were solved by direct methods (SHELXT-2014) and refined *via* full-matrix least-squares difference Fourier techniques using SHELXL-2018/3. Crystallographic data for the structures reported in this paper have been deposited with the Cambridge Crystallographic Data Centre. Copies of the data can be obtained, free of charge, on application to the Director, CCDC, 12 Union Road, Cambridge CB2 1EZ, United Kingdom (fax: +44-(0)1223-336033 or e-mail: deposit@ccdc.cam.ac.uk).

*Crystallographic data for* **3**: C_9_H_8_O_5_, *M*_r_ = 196.15, prism from MeOH/H_2_O (50:1), space group *C*c, *a* = 3.8907(3) Å, *b* = 15.5114(11) Å, *c* = 13.8032(10) Å, *V* = 827.53(11) Å^3^, *Z* = 4, *μ* = 0.718 mm^−1^, *F*(000) = 408.0; crystal size: 0.120 mm^3^ × 0.110 mm^3^ × 0.090 mm^3^; 1,478 unique reflections with 1,368 obeying the *I* ≥ 2*σ*(*I*); *R* = 0.0336(1368), *wR*2 = 0.0849(1478), *S* = 1.055; supplementary publication no. CCDC-2027079.
*Crystallographic data for* **4**: C_18_H_20_O_6_, *M*_r_ = 332.34, prism from MeOH/DCM (40:1), space group *P*_−1_, *a* = 4.8536(2) Å, *b* = 11.5267(5) Å, *c* = 14.9026(7) Å, *V* = 769.37(6) Å^3^, *Z* = 2, *μ* = 0.572 mm^−1^, *F*(000) = 352.0; crystal size: 0.120 mm^3^ × 0.110 mm^3^ × 0.080 mm^3^; 2,792 unique reflections with 2,287 obeying the *I* ≥ 2σ(*I*); *R* = 0.0377(2287), *wR*2 = 0.1016(2792), *S* = 1.030; supplementary publication no. CCDC-2019163.

### *In vitro* Cytotoxicity Test Protocols

Compounds **1** and **3**–**8** were tested in serial dilutions from the maximum concentration of 20 μM for their inhibition toward CCRF-CEM human T lymphoblast cells *via* lactate dehydrogenase testing and U87 human glioblastoma with MTT according to published protocols (Boudreau et al., 2012; Williams et al., 2017).

### *In vitro* Antimicrobial Assay Protocols

Antibacterial susceptibility was tested against several Gram-positive and Gram-negative human or aquatic pathogens, namely *Pseudoalteromonas carrageenovora*, *Vibrio shilanii*, *V. scophthalmi*, *V. alginolyticus*, *Salmonella typhi*, *Pseudomonas aeruginosa*, *Staphylococcus aureus*, and *Bacillus pumilus*. Compounds **1** and **3**–**8** were dissolved in DMSO and tested at a concentration of 64, 32, 16, 8, and 4 μg/ml according to a published protocol (CLSI, 2018; Bibi et al., 2020). Briefly, the bacteria were grown in MH medium (beef powder 6.0 g, soluble starch 1.5 g, acid hydrolyzed casein 17.5 g, and H_2_O up to a total volume of 1 L) for 24 h at 28°C with agitation (180 rpm), then diluted with sterile MH medium to match 0.5 McFarland standard. 100 μl of each bacteria supernatant, 100 μl MH medium with 0.001% 2,3,5-triphenyltetrazolium chloride (an indicator of viable bacteria), together with test or control materials were incubated in 96-well plates. The treated bacteria were cultured statically at 28°C for 24 h, then the inhibition data were recorded optically. Norfloxacin (from Shanghai Yuanye Bio-Technology Co., Ltd.) was used as positive control, and this was dissolved in DMSO at the same concentrations as the tested compounds. Blank media with the same volume of DMSO as the test samples was used as the negative control.

### Iron Chelation Evaluation

Ferrozine can chelate Fe^2+^ to afford a complex with an absorbance at 562 nm, which allows for a facile chemical assay that was repeated according to published protocols (Da Lozzo et al., 2002; Azran et al., 2015). In brief, the reaction system on a 96-well plate was composed of 160 μl CH_3_COONa (100 mM), 40 μl FeCl_2_ (1.5 mg/ml), and 10 μl of compounds **1** and **3**–**8** (0.3, 1, 3, and 10 μM), separately. At the same concentrations, EDTA, a known chelator of Fe^2+^ was used as a positive control. The reaction medium, CH_3_COONa (100 mM), was used as a negative control. Following sample addition and 30-min light-proof standing, 10 μl ferrozine (40 mM) was added and the absorbance at 562 nm was collected after another 5-min light-proof standing.

### *In vivo* Activation of the GUS Reporter in PR1::GUS Transgenic *Arabidopsis thaliana* Plants

Following a published method (Wang et al., 2018), clean *Arabidopsis thaliana* seeds ProPR1::GUS (purchased from Arabidopsis Biological Resource Center) were sown in Murashige and Skoog medium (PhytoTechnology Laboratories, United States), with or without added individual compounds **1** and **3**–**8** at 10 μM and maintained at 4°C. After 3 days, the culture conditions were altered to 22°C with a 16 h light/8 h dark photoperiod. After another 10 days, the plants were dyed in GUS histochemical staining stock at 37°C for 3 h, and the chlorophyll of the plants were washed with 70% ethanol. Observation and photo documentation of the results were carried out with an optical microscope.

## Results and Discussion

### Sample Prioritization

**Figure 2:**
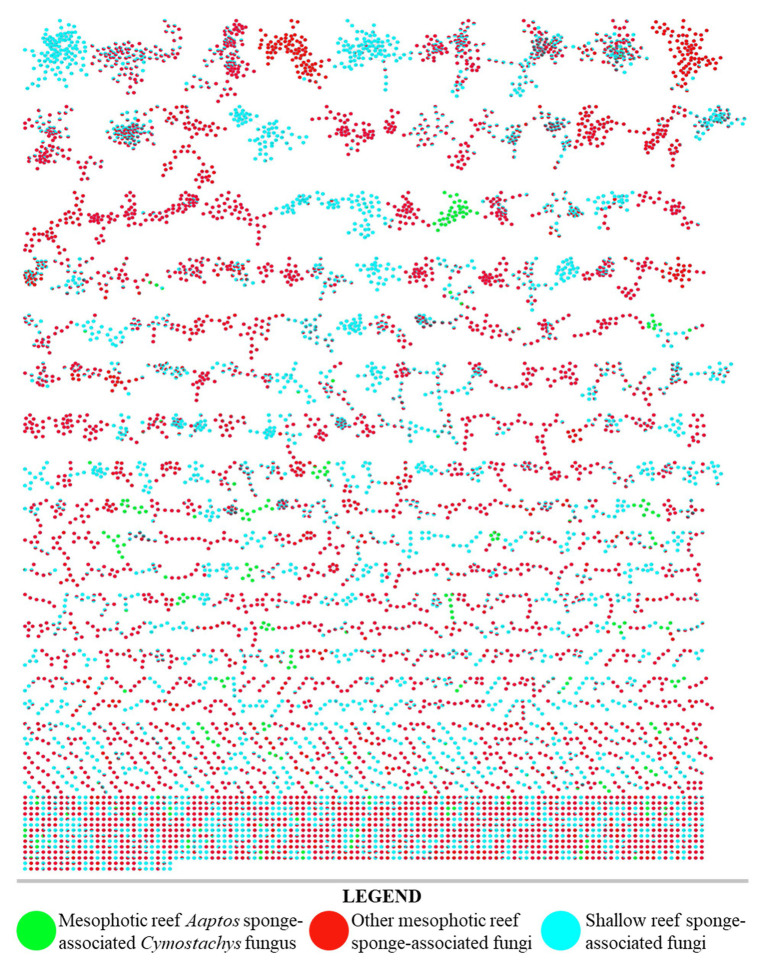
LC–MS/MS derived molecular network of organic extracts produced from 80 fungal cultures (40 from shallow reef sponges, 40 from mesophotic zone sponges). Single node clusters, or self-loop nodes, were excluded for brevity.

From a series of sponges that were collected at diverse depths of 7–103 m (Supplementary Table S1), some 80 fungal strains were isolated in laboratory culture by conventional methods (Höller et al., 2000; Liu et al., 2019). The fungal strains were divided into two groups of each *n* = 40, representing shallow reef and MCE origins of the sponges that yielded the isolated microbial samples. Each strain (*n* = 80) was cultivated in small-scale replicates using three different types of growth media to evaluate their secondary metabolite production potential by the OSMAC approach. Organic extracts were prepared from all 240 strain-media combination cultures and evaluated by TLC. The most natural product-rich culture of each strain was selected for further evaluation by untargeted LC-MS/MS analysis (*n* = 80). All obtained data were analyzed together using the Global Natural Product Social Molecular Networking (GNPS) web interface (Wang et al., 2016). A molecular network was prepared and annotated (Figure 2), from which it was obvious that the extracts of fungal strains prepared from MCE sponge samples were largely distinct from their shallow water counterparts. Although this remains a relatively small sample set of *n* = 40 per group, the major compositional difference between the MCE and shallow reef samples here analyzed is postulated to be a widespread phenomenon. Further sample collections and laboratory investigations are planned for evaluating this hypothesis.

One organism from the MCE subset of the above strain library was selected for further study in part because the morphology of the producing organism separated it from typically studied genera of fungal natural product producers. Upon close examination of the literature, it was determined that the morphology of strain NBUF082 matched what was described for the genus *Cymostachys* when it was established in recent reports, and it was also conclusively identified as a *Cymostachys* sp. by the ITS region sequence of this organism (Lin et al., 2016; Lombard et al., 2016). Although *Cymostachys* is closely related to the genus *Stachybotrys*, only the latter has been extensively studied for its natural product chemistry (Wang et al., 2015; Zhao et al., 2017; Jagels et al., 2019; Zheng et al., 2019). In contrast, no natural products reported from *Cymostachys* could be found at the onset of this study, and it was proposed that this organism could produce new and interesting natural products chemistry.

Furthermore, the result of using the OSMAC approach to screen the 240 strain x culture condition set of combinations showed that the *Cymostachys* sp. NBUF082 was a moderately high yielding producer of natural products when grown on potato dextrose agar (PDA). With this media, the organism generated 451.8 mg/L organic extract, compared with a range from about 5 mg/L to 1 g/L for other strain x culture condition combinations. When the extract from this sample was evaluated by LC-MS/MS molecular networking, it was selected for further study based on the observation of several distinct molecules compared to the remainder of the data set. The nodes in the network owing to this sample have been colored green in Figure 2 to show how it stood out from all the 40 shallow reef and the remaining 39 MCE originating sponge-derived fungi. Many more broadly distributed metabolites and molecular feature node clusters were observed to be shared between data sets resulting from shallow and MCE samples, and these are apparent as split-color pie graph nodes in Figure 2. Some metabolites were found to be more broadly distributed, but exclusively observed in the same depth-based subsets, and these were purposefully overlooked for prioritization in this research study. For example, metabolites coming from two or more shallow-derived samples and not the MCE subset were lumped together (shown in only blue in Figure 2) and avoided for targeted isolation here, as were those resulting from two or more MCE-derived samples but not the shallow subset (shown in only red in Figure 2). The prioritized fungal strain, *Cymostachys* sp. NBUF082, was cultivated in large-scale for natural product discovery. Some other fungal samples along with their OSMAC culture conditions from the 240 combination set were also considered to be chemically interesting, and these were ranked lower in priority to be investigated and reported on in due time.

### Structure Elucidation

**Figure 3:**
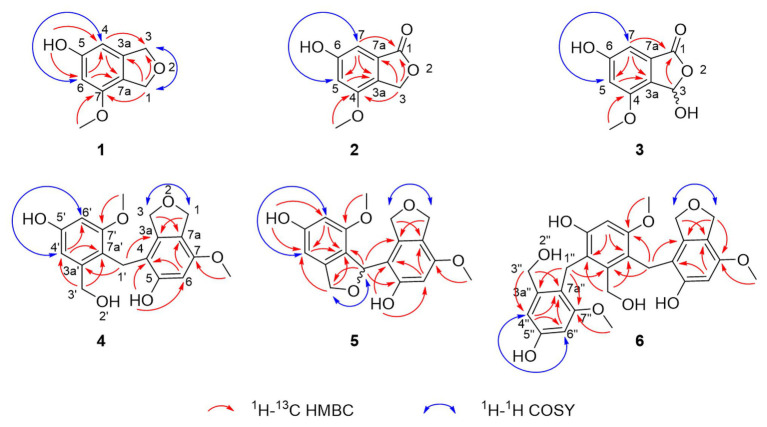
Selected correlations used to determine the planar structures of cymopolyphenols A-F (1–6). Red single-sided arrows represent cross peaks from the ^1^H-^13^C HMBC spectrum. Blue double-sided arrows show protons correlated in the ^1^H-^1^H COSY spectrum, and long-range *J* coupling that was determined for each from the ^1^H spectrum.

Compound **1** was obtained as a white powder and assigned the molecular formula C_9_H_10_O_3_ based on a proton adduct peak in the HRESIMS spectrum at *m/z* 167.0706 [M + H]^+^ (calcd. for C_9_H_11_O_3_, 167.0703). This formula indicated that **1** possessed five degrees of unsaturation. The ^1^H and ^13^C NMR data of **1** (Table 1) demonstrated the existence of one methoxy group (*δ*_H_ 3.71, *δ*_C_ 55.0; 7-*O*-CH_3_), two oxygenated methylenes (*δ*_H_ 4.84, *δ*_c_ 70.9; CH_2_-1 and *δ*_H_ 4.88, *δ*_C_ 73.1; CH_2_-3), two aromatic methines (*δ*_H_ 6.26, *δ*_C_ 99.7; CH-4 and *δ*_H_ 6.27, *δ*_C_ 97.7; CH-6), and four nonprotonated sp^2^ carbons (*δ*_C_ 141.5; C-3a, *δ*_C_ 159.1; C-5, *δ*_C_ 154.3; C-7, and *δ*_C_ 116.3; C-7a). A substituted phenolic group was deduced from the 1D NMR data, which accounted for four of five required degrees of unsaturation. The downfield protons CH_2_-1 and CH_2_-3 exhibited long range coupling with each other (*J* = 2.3 Hz), as is typical for the methylene units of a dihydroisobenzofuran, and the dihydrofuran subunit accounted for the last degree of unsaturation required for **1**. Observed correlations in the ^1^H-^13^C HMBC spectrum for H-1 with C-3, C-3a, and C-7, H-4 with C-3 and C-7a, H-6 with C-4 and C-7a, the protons of 7-*O*-CH_3_ with C-7, and of 5-OH with C-4 and C-6 (Figure 3) were used to determine the substitution pattern for the aromatic ring. Altogether, this established the structure of **1** as 7-methoxy-1,3-dihydroisobenzofuran-5-ol, a new fungal natural product here named cymopolyphenol A, which is a methoxy analog of the 1,3-dihydroisobenzofuran-4,6-diol previously reported from *Neolentinus lepideus* (Li et al., 2013).

**Table 1:** ^1^H and ^13^C NMR spectroscopic data for **1**–**3** in DMSO-*d*_6_*^a^*.

Position	1		2		3	
	*δ*_C_, type	*δ*_H_ (*J* in Hz)	*δ*_C_, type	*δ*_H_ (*J* in Hz)	*δ*_C_, type	*δ*_H_ (*J* in Hz)
1	70.9, CH_2_	4.84 t (2.3)	170.8, C		168.1, C	
3	73.1, CH_2_	4.88 t (2.3)	68.0, CH_2_	5.23 s	102.0, CH	6.44 s
3a	141.5, C		125.9, C		122.5, C	
4	99.7, CH	6.26 s*^b^*	154.9, C		156.1, C	
5	159.1, C		105.0, CH	6.75 d (1.8)	105.2, CH	6.75 d (1.8)
6	97.7, CH	6.27 s*^b^*	160.3, C		161.8, C	
7	154.3, C		101.4, CH	6.71 d (1.8)	101.6, CH	6.70 d (1.8)
7a	116.3, C		127.2, C		129.2, C	
7-OCH_3_	55.0, CH_3_	3.71 s				
4-OCH_3_			55.8, CH_3_	3.83 s	55.8, CH_3_	3.83 s
5-OH		9.46 br s				
6-OH				10.21 br s		10.39 s

a Data recorded at 298 K, 600 MHz (^1^H) and 150 MHz (^13^C). Assignments supported by 2D NMR.

b Partially overlapped.

Compound **2** was also obtained as a white powder. The molecular formula of **2** was determined to have two less hydrogen atoms and one more oxygen than **1**, or C_9_H_8_O_4,_ after observation of the proton adduct peak in the HRESIMS spectrum at *m/z* 181.0500 [M + H]^+^ (calcd. for C_9_H_9_O_4_, 181.0495). This formula requires six degrees of unsaturation, which is one more than **1** has. The ^1^H and ^13^C NMR spectra of **2** resemble those of **1**, except that the absence of one oxygenated methylene group accompanied the addition of a carbonyl at *δ*_C_ 170.8 (C-1) for **2** (note that different carbon numbering schemes emerged for **1** and **2** due to the priority of this carbonyl), and the long range coupling observed for CH_2_-1 in **1** was not observed for the corresponding CH_2_-3 in **2** (Table 1). The ^1^H-^13^C HSQC and HMBC spectra of **1** and **2** were also similar, and key correlations from H-3 to C-4 and H-7 to C-1 (Figure 3) led to the structure elucidation of **2** as an analog of **1** with C-3 being oxidized to an ester carbonyl and re-numbered as C-1. The incremented oxidation state of C-1 in **2** satisfied the difference in molecular formula and the corresponding additional unsaturation required compared to **1**, as well as an observed respective upfield shift of C-7a and downfield shifts of H-7, C-3a, C-5, and C-7. It was considered that the alternative position on the hydrofuran ring in **2** might instead be the oxidized carbon in the furan-1(3*H*)-one ring, if some 4-bond HMBC correlations were observed. However, the corresponding compound with C-1 bearing the lactone carbonyl adjacent the methoxy group rather than the phenyl proton has been reported in the literature as an intermediate in the total synthesis of notholaenic acid, and this alternative compound (measured in the same NMR solvent) has spectroscopic data that is distinct from **2** (El-Feraly et al., 1985). Thus compound **2** was established as 6-hydroxy-4-methoxyisobenzofuran-1(3*H*)-one, a new fungal natural product congener of the sparalides reported from *Sparassis crispa* (Wulf.) (Bang et al., 2017), here named as cymopolyphenol B.

**Figure 4:**
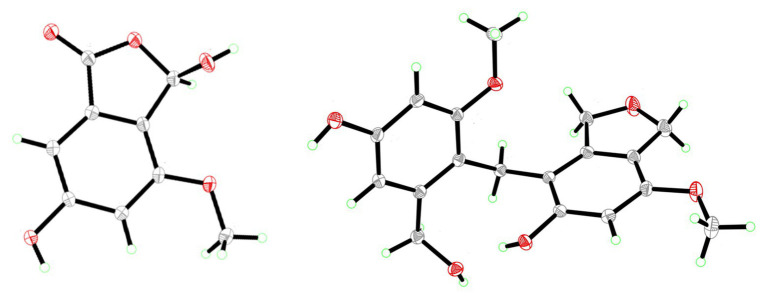
X-ray ORTEP drawings of compounds 3 (left) and 4 (right).

Compound **3** was purified in crystalline form as colorless prisms. The molecular formula for this compound was obtained as C_9_H_8_O_5_ due to the proton adduct peak observed in the HRESIMS at *m*/*z* 197.0453 [M + H]^+^ (calcd. for C_9_H_9_O_5_, 197.0444). Compared with compound **2**, this requires the same six degrees of unsaturation but one additional oxygen atom. The NMR data of **2** and **3** are quite similar (Table 1), with the noteworthy difference being that the oxygenated methylene of **2** (*δ*_H_ 5.23, *δ*_c_ 68.0; CH_2_-3) was absent in **3**, and instead a significantly deshielded oxygenated methine group was observed (*δ*_H_ 6.44, *δ*_c_ 102.0; CH-3). The HSQC and ^1^H-^13^C HMBC spectra of **2** and **3** were also otherwise similar, and key correlations from H-3 to C-1 and H-7 to C-1 (Figure 3) led to the structure elucidation of **3** as an analog of **2** with C-3 being oxidized as a hemiacetal. Since this compound was obtained in crystalline form and is relatively devoid of signals in the ^1^H NMR spectrum, it was decided to investigate the configuration of C-3 by X-ray crystallography rather than using a Mosher ester analysis. The crystallographic study of **3** confirmed the planar structure of this molecule (Figure 4). It was also found that this material was obtained in crystalline form as a racemic mixture, as indicated by the non-centrosymmetric space group *Cc* that would be invalid if **3** were enantio-pure (Parsons, 2017). The optical rotation measured for **3** {[*α*]25D (*c* 0.1, MeOH)} was also zero, further supporting the assignment of the racemic mixture. This likely resulted from keto-enol tautomerization or lactone ring opening and re-closure in solution and during the extraction and purification process rather than non-stereospecific biosynthesis. The instability of **3** was further noted with the observation of an impurity of the proposedly 3-*O*-methyl analog in the NMR spectra measured first in CD_3_OD (then diluted in MeOH for sample transfer) and later in DMSO-*d*_6_, since corresponding peaks were not in the HRESIMS, nor was this impurity observed in the same sample by X-ray crystallography. The new natural product, **3**, was in summary established as 3,6-dihydroxy-4-methoxyisobenzofuran-1(3*H*)-one, here named as cymopolyphenol C.

Compound **4** was afforded in crystalline form as colorless prisms. The molecular formula was determined to be C_18_H_20_O_6_ from its sodium adduct peak at *m/z* 355.1159 [M + Na]^+^ (calcd. for C_18_H_20_O_6_Na, 355.1152) in the HRESIMS. This formula calls for nine degrees of unsaturation. The ^1^H and ^13^C NMR data (Table 2) revealed that **4** possesses two methoxy groups (*δ*_H_ 3.678, *δ*_C_ 54.9; 7-*O*-CH_3_; and *δ*_H_ 3.682, *δ*_C_ 55.3; 7'-*O*-CH_3_), one aliphatic methylene (*δ*_H_ 3.674, *δ*_C_ 21.1; CH_2_-1'), three oxygenated methylenes (*δ*_H_ 4.70, *δ*_C_ 70.3; CH_2_-1, *δ*_H_ 4.16, *δ*_C_ 71.8; CH_2_-3, and *δ*_H_ 4.23, *δ*_C_ 60.0; CH_2_-3'), three aromatic methines (*δ*_H_ 6.37, *δ*_C_ 97.7; CH-6, *δ*_H_ 6.52, *δ*_C_ 105.3; CH-4' and *δ*_H_ 6.28, *δ*_C_ 96.9; CH-6′), and nine nonprotonated sp^2^ carbons [*δ*_C_ 139.5 (C-3a), 112.8 (C-4), 155.6 (C-5), 151.4 (C-7), 116.4 (C-7a), 143.3 (C-3a'), 156.7 (C-5'), 158.2 (C-7'), and 114.2 (C-7a')]. From the NMR data, several key features from compounds **1**–**3** were observed in **4**. The HMBC correlations from H-1 to C-3a, from H-3 to C-7a, from 5-OH to C-4 and C-6, from H-6 to C-4 and C-7a, and 7-*O*-CH_3_ to C-7, together with the long-range spin system of H-1 and H-3 (*J* = 2.4 Hz) indicated a 7-methoxy-1,3-dihydroisobenzofuran-5-ol moiety in **4**. Inspection of the remaining NMR signals led to the establishment of a related tetra-substituted phenolic moiety that is representative of a ring opening between C-1 and O-2 in the dihydrofuran subunit of compound **1**, here presumed to be a monomeric subunit of **4**. The open-ring and closed-ring subunits mentioned above were determined to be connected from methylene C-1' to the nonprotonated aromatic C-4, with evidence of HMBC correlations from H-1' to both C-3a and C-3a'. The structure of **4** was thus established as a homodimer of 7-methoxy-1,3-dihydroisobenzofuran-5-ol (**1**) with ring opening between C-1' and O-2'. Compound **4** is here named as cymopolyphenol D, systematically 4-[4-hydroxy-2-(hydroxymethyl)-6-methoxybenzyl]-7-methoxy-1,3-dihydroisobenzofuran-5-ol. The structure of this new natural product was further confirmed by single-crystal X-ray diffraction analysis (Figure 4).

**Table 2:** ^1^H and ^13^C NMR Spectroscopic Data for **4**–**6** in DMSO-*d*_6_*^a^*.

Position	4		5		6	
	*δ*_C_, type	*δ*_H_ (*J* in Hz)	*δ*_C_, type	*δ*_H_ (*J* in Hz)	*δ*_C_, type	*δ*_H_ (*J* in Hz)
1	70.3, CH_2_	4.70 t (2.4)	70.2, CH_2_	4.72 m	70.1, CH_2_	4.67 t (2.3)
3	71.8, CH_2_	4.16 t (2.4)	71.6, CH_2_	4.49 br d (12.7) 3.99 br d (12.7)	71.9, CH_2_	4.00 t (2.3)
3a	139.5, C		140.0, C		139.5, C	
4	112.8, C		114.3, C		114.1, C	
5	155.6, C		156.2, C		155.4, C	
6	97.7, CH	6.37 s	97.8, CH	6.36 s	97.6, CH	6.38 s
7	151.4, C		153.0, C		151.2, C	
7a	116.4, C		116.6, C		116.2, C	
5-OH		9.43 s		9.45 s		
7-OCH_3_	54.9, CH_3_	3.678 s*^b^*	54.9, CH_3_	3.71 s	54.8, CH_3_	3.68 s
1'	21.1, CH_2_	3.674 br s*^b^*	76.4, CH	6.41 dd (3.0, 1.9)	22.6, CH_2_	3.84 s*^c^*
3'	60.0, CH_2_	4.23 s	72.4, CH_2_	5.00 dd (12.4, 3.0) 4.84 dd (12.4, 1.9)	57.1, CH_2_	4.39 s
3a'	143.3, C		142.4, C		141.2, C	
4'	105.3, CH	6.52 d (2.4)	99.3, CH	6.27 d (1.8)	119.0, C	
5'	156.7, C		159.3, C		154.8, C	
6'	96.9, CH	6.28 d (2.4)	97.9, CH	6.23 d (1.8)	98.0, CH	6.30 s
7'	158.2, C		154.9, C		156.0, C	
7a'	114.2, C		117.8, C		117.0, C	
5'-OH		9.23 s		9.49 s		
7'-OCH_3_	55.3, CH_3_	3.682*^b^* s	55.2, CH_3_	3.52 s	55.0, CH_3_	3.61 s
1''					22.4, CH_2_	3.76 s*^c^*
3''					61.1, CH_2_	4.54 s
3a''					142.2, C	
4''					105.9, CH	6.44 d (2.4)
5''					155.8, C	
6''					97.3, CH	6.14 d (2.4)
7''					158.2, C	
7a''					117.3, C	
7''-OCH_3_					54.5, CH_3_	3.35 s

a Data recorded at 298 K, 600 MHz (^1^H) and 150 MHz (^13^C) or 500 MHz (^1^H) and 125 MHz (^13^C). Assignments supported by 2D NMR.

b Signals partially overlapped.

c Might be interchanged.

Compound **5** was isolated as a light-brown powder. The molecular formula of **5** was established as C_18_H_18_O_6_ based on a sodium adduct peak observed in the HRESIMS at *m/z* 353.0997 [M + Na]^+^ (calcd. for C_18_H_18_O_6_Na, 353.0996). This formula requires 10 degrees of unsaturation, or one more than for the structure of **4**. Comparison of the ^1^H and ^13^C NMR data (Table 2) for **5** with those of **4** showed strong similarities except for the presence in **5** of one additional oxymethine (*δ*_H_ 6.41, *δ*_C_ 76.4; C-1') that accompanied the absence of an aliphatic methylene from **4**. Furthermore, the oxymethine C-1' exhibited long range coupling (*J* = 3.0, 1.9 Hz) with the diastereotopic protons of oxymethylene C-3′ [*δ*_H_ 5.00 (dd, *J* = 12.4, 3.0 Hz) and 4.84 (dd, *J* = 12.4, 1.9 Hz), *δ*_C_ 72.4], which was consistent with the protons of C-1 and C-3 coupling in compounds **1** and **4**, while the corresponding groups (C-1' and C-3') were not coupled and were observed as singlets in **4** (Figure 3). It was accordingly suggested that **5** is an analog of **4**, and another homodimer of **1**, but with both dihydroisobenzofuran subunits intact. This proposal accounted for the additional degree of unsaturation required for **5**, and was further supported in concept and attachment point by the HMBC correlations from H-1' to C-3a, C-5, and C-7a'. Therefore, the structure of **5** was established as a new homodimer of 7-methoxy-1,3-dihydroisobenzofuran-5-ol (**1**), as shown. Since C-1' is a chiral center, the optical rotation of **5** was measured, and this compound was found to be racemic {[*α*]25D 0 (*c* 0.1, MeOH)}. It was attempted to purify the enantiomers of **5** by HPLC using a chiral column, but the completely resolved separated peaks were found upon reinjection to have undergone racemization in solution. Accordingly, this racemic mixture (**5**) was assigned the common name cympolyphenol E and systematic name 7,7'-dimethoxy-1,1',3,3'-tetrahydro-[1,4'-biisobenzofuran]-5,5'-diol.

Compound **6** was obtained as a white powder and found to have the molecular formula C_27_H_30_O_9_ based on the observed sodium adduct peak at *m/z* 521.1790 in the HRESIMS (calcd. For C_27_H_30_O_9_Na, 521.1782). The NMR data of **6** had the hallmarks of both **4** and **5** (Table 2), and indicated the presence of one 7-methoxy-1,3-dihydroisobenzofuran-5-ol and two 3-(hydroxymethyl)-5-methoxyphenol moieties as monomeric substructures presumably all derived from **1**. The structural subunits were able to be connected unequivocally from C-1' to C-4 and C-1'' to C-4' by the observation of HMBC correlations from H-1' to C-3a, C-3a', and C-7' along with those from H-1'' to C-3a', C-3a'', and C-7'' (Figure 3). The structure of **6** was thus established as a homotrimer of 7-methoxy-1,3-dihydroisobenzofuran-5-ol (**1**), with one intact dihydroisobenzofuran moiety at the terminal monomeric subunit, as shown. This achiral molecule was assigned the common name of cymopolyphenol F, or 4-(4-hydroxy-3-(4-hydroxy-2-(hydroxymethyl)-6-methoxybenzyl)-2-(hydroxymethyl)-6-methoxybenzyl)-7-methoxy-1,3-dihydroisobenzofuran-5-ol.

It was considered whether compounds **4**–**6**, representing low-order polymers of **1**, might be extraction artifacts as opposed to biosynthetic products of fungal metabolism. These molecules could be biosynthesized *de novo*, using **1** as an intermediate, through radical coupling, or by acid/base reactions *via* a quinone methide pathway catalyzed by fungal enzymes. Alternatively, the compounds might be generated incidentally by the extraction and isolation protocol. However, the corresponding LC-MS peaks for these compounds were observed in the crude extract prior to purification by the relatively harsher conditions of repeated chromatographic separation. Furthermore, preliminary attempts to chemically synthesize compounds **4**–**6** from **1** at identical medium PDB used in scale-up fermentation, acid (pH = 4.0) and base (pH = 10.0) solvents at 28°C with agitation (120 rpm) for 15 days were unsuccessful.

Compounds **7** and **8** were identified as previously described molecules, respectively F1839-I and stachybotrylactone, by comparison of obtained NMR, MS, and optical rotation data with literature values (Ayer and Miao, 1993; Sakai et al., 1995; Zhao et al., 2017). Interestingly, stachybotrylactone was established at the time of its discovery as a spontaneous Cannizzaro reaction degradation product of the related molecule, stachybotrydial (Ayer and Miao, 1993). However, this known phenylspirodrimane fungal natural product contains as a substructure one of the new molecules here reported, **2**.

### Biological Evaluation

Compound **2** was not obtained in sufficient quantity for biological testing in the present study. However, the purified compounds **1** and **3**–**8** were tested *in vitro* with a small array of bioassays to evaluate their potential for use in medicine, agriculture, aquaculture, or other biotechnology applications. For example, *in vitro* against the U87 human glioblastoma and CCRF-CEM human T lymphoblast cell lines, none of the tested compounds were found to be antiproliferative or cytotoxic (IC_50_ > 20 μM). Accordingly, these compounds were evaluated for activity against an array of aquatic and human pathogens including Gram-negative and Gram-positive bacteria: *P. carrageenovora*, *V. shilanii*, *V. scophthalmi*, *V. alginolyticus*, *S. typhi*, *P. aeruginosa*, *S. aureus*, and *B. pumilus*. Compounds **1** and **3**–**6** were found to be weakly antimicrobial (MIC 16–64 μg/ml) *in vitro* against some of these pathogens, as detailed in Table 3. While these compounds are not active against the human pathogens at concentrations with pharmaceutical relevance, it is of interest to find selective agents for the potential treatment of aquatic pathogens for use in preventing economic losses in the aquaculture industry without risking the induction of drug resistance in human pathogens. Compounds **4** and **6** were the most active tested against the aquatic pathogens, which may indicate an ecological role of these new natural products and potential direction for further development based on the same scaffolds.

**Table 3:** *In vitro* antimicrobial activity observed for 1 and 3–8.

Cpd	Minimum inhibition concentration (MIC, μg/ml)							
	*Pseudoalteromonas carrageenovora*	*Vibrio shilanii*	*Vibrio scophthalmi*	*Vibrio alginolyticus*	*Salmonella typhi*	*Pseudomonas aeruginosa*	*Staphylococcus aureus*	*Bacillus pumilus*
**1**	>64	>64	>64	64	>64	>64	>64	>64
**3**	32	>64	64	64	32	64	>64	>64
**4**	32	32	32	64	64	64	>64	>64
**5**	64	>64	>64	>64	>64	>64	32	>64
**6**	32	32	16	64	32	64	>64	>64
**7**	>64	>64	>64	>64	>64	>64	>64	>64
**8**	>64	>64	>64	>64	>64	>64	>64	>64
**PC***^a^*	0.5	1	2	1	1	1	1	1
**NC***^b^*	>64	>64	>64	>64	>64	>64	>64	>64

a PC: norfloxacin, used as a positive control. The MIC of this compound was not tested below 0.5 μg/ml in this experiment, but it has been shown to be active ≤ 0.125 μg/ml in all organisms tested by the same experiment conducted at a different time.

b NC: blank media, used as a negative control.

**Figure 5:**
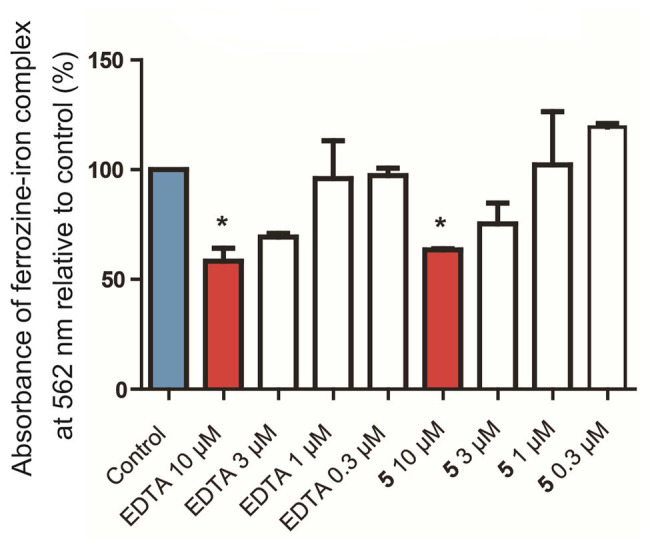
*In vitro* chelation of 5 to Fe^2+^ in a ferrozine-based chemical assay. Control: Blank media, sodium acetate in water, used as a negative control. EDTA: Ethylenediaminetetraacetate, used as a positive control. *p < 0.05 vs. control.

Due to the amount of oxygen atoms in the isolated compounds, especially **4**–**6**, it was considered whether these molecules could chelate iron. The chelation of iron by secondary metabolites has ecological implications, e.g., with siderophores, and can also play a role in various aspects of human health. For example, the deposition of iron in nerves causes oxidative stress and inflammation, leading to the kind of nerve damage that can be found in traumatic brain injury, Alzheimer’s disease, and Parkinson’s disease. When the pure molecules were tested in a ferrozine Fe^2+^ chelation chemical assay, it was found that compound **5** concentration-dependently chelated iron (Figure 5) with nearly the same efficacy at 10 μM as the positive control, ethylenediaminetetraacetate (EDTA). Interestingly, compound **4** was inactive in the same assay at 10 μM. This suggests that while the flexibility afforded to **4** by its structural subunits being linked with the C-1' methylene group rather than the C-1' dihydroisofuran methine in **5** may give it preferential antibacterial activity, the relatively locked conformation of **5** is more suitable for iron chelation. This also suggests that iron chelation is not the primary mechanism of antibiotic action of **4**.

**Figure 6:**
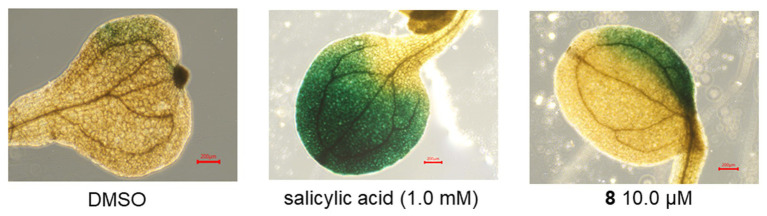
*In vivo* activation of the GUS reporter in PR1::GUS transgenic *Arabidopsis thaliana* plants. DMSO was used as a negative control. Salicylic acid was used as a positive control.

Finally, the compounds were evaluated with an *in vivo* assay of inducing disease resistance in plants using the PR1::GUS transgenic model organism, *A. thaliana*. Pathogenesis-related protein 1 (PR1) is correlated to plant disease resistance, and β-glucuronidase (GUS) is attached as a reporter gene (Van Loon, 1997; Koornneef et al., 2008). As shown in Figure 6, plants treated with compound **8** at 10 μM were found to accumulate PR1, indicating the potential of this molecule to enhance plant disease resistance. The remaining compounds, including the close structural analog, **7**, were not found to activate PR1 in the same test model at 10 μM. This newly discovered function of **8** merits further investigation of structurally related molecules in this and similar ecological studies.

## Conclusion

From preliminary MS/MS-based molecular networking analysis of 80 extracts prepared from marine sponge-associated fungal cultures, half from shallow reefs and half from MCEs, it was found that the extracts of fungal strains prepared from mesophotic zone sponge samples contained different chemistry than their shallow water counterparts. It is hypothesized that this is a representative phenomenon that should encourage the further chemical investigation of mesophotic zone organisms, and the purposeful generation and analysis of a larger data set is planned. The investigation of a prioritized *Cymostachys* fungus that was isolated from its association with a 103 m deep *Aaptos* sponge led to the discovery and structural characterization of a new series of compounds, cymopolyphenols A−F (**1**–**6**) along with the known fungal natural products F1839-I (**7**) and stachybotrylactone (**8**). Compounds **1**–**6** and **8** all contain a dihydroisobenzofuran skeleton, and **4**–**6** appear to be low-order polymers of **1** that present new scaffolds. Structural analogs of these compounds with different oxidation states, increased order of polymerization, and methylation patterns are predicted to emerge from future research on related organisms.

Compounds **1**–**6** are hydrogen deficient molecules, and each has a proton-to-heavy-atom ratio under 1, yet the structures of these molecules were able to be established by spectroscopic and spectrometric data interpretation. This fortuitous occurrence was due in equal parts to the dispersion of signals in the ^1^H NMR spectrum without significant overlapping, and the distribution of the associated hydrogen atoms throughout the molecules that allowed for informative long-range correlations to be observed. Still, further support for all the structures was garnered from the X-ray crystallographic study of 3 and **4**. Compounds **1** and **3**–**6** were found to be weakly antimicrobial (MIC 16–64 μg/ml) *in vitro* against several Gram-positive and Gram-negative human or aquatic pathogens. These data are not suggestive of a meaningful lead for pharmaceutical development, but could potentially be useful in the development of aquaculture treatments or represent clues to an ecological role of the compounds.

## Data Availability Statement

The datasets presented in this study can be found in online repositories. The names of the repository/repositories and accession number(s) can be found in the article/Supplementary Material.

## Author Contributions

All authors conceived the research, analyzed the data, contributed to the study, and approved the final version of the manuscript. TW, JZh, JZo, YS, PS, TY, XL, and JY carried out the experiments. WZ, WC, XW, XY, CN, JL, and SH revised the manuscript.

### Conflict of Interest

The authors declare that the research was conducted in the absence of any commercial or financial relationships that could be construed as a potential conflict of interest.

## Footnotes

^1^https://www.midwestern.edu/departments/marinepharmacology.xml

^2^http://www.tascmar.eu/

^3^http://gnps.ucsd.edu/

^4^https://cytoscape.org/
