# Supplementary material for: Discovery of Cymopolyphenols A–F From a Marine Mesophotic Zone Aaptos Sponge-Associated Fungus Cymostachys sp. NBUF082
Source: Front Microbiol. 2021 Feb 22;12:638610. doi: 10.3389/fmicb.2021.638610 (PMC7937805; doi:10.3389/fmicb.2021.638610)
Supplement: Supplementary file 1 [file Data_Sheet_1.pdf]

Table of Contents

Table S1. Sponge sampling and taxonomic information. 3

The ITS region of *Cymostachys* sp. NBUF082 4

Figure S1. Chiral HPLC analysis of **5** and its separated enantiomers. 5

Figure S2. ^{1}H NMR spectrum of cymopolyphenol A (**1**) in DMSO-d_6 (600 MHz). 6

Figure S3. ^{13}C NMR spectrum of cymopolyphenol A (**1**) in DMSO-d_6 (150 MHz). 7

Figure S4. DEPT135 spectrum of cymopolyphenol A (**1**) in DMSO-d_6 (150 MHz). 8

Figure S5. ^{1}H-^{1}H COSY spectrum of cymopolyphenol A (**1**) in DMSO-d_6. 9

Figure S6. ^{1}H-^{13}C HSQC spectrum of cymopolyphenol A (**1**) in DMSO-d_6. 10

Figure S7. ^{1}H-^{13}C HMBC spectrum of cymopolyphenol A (**1**) in DMSO-d_6. 11

Figure S8. NOESY spectrum of cymopolyphenol A (**1**) in DMSO-d_6. 12

Figure S9. ^{1}H NMR spectrum of cymopolyphenol B (**2**) in DMSO-d_6 (600 MHz). 13

Figure S10. ^{13}C NMR spectrum of cymopolyphenol B (**2**) in DMSO-d_6 (150 MHz). 14

Figure S11. DEPT135 spectrum of cymopolyphenol B (**2**) in DMSO-d_6 (150 MHz). 15

Figure S12. ^{1}H-^{1}H COSY spectrum of cymopolyphenol B (**2**) in DMSO-d_6. 16

Figure S13. ^{1}H-^{13}C HSQC spectrum of cymopolyphenol B (**2**) in DMSO-d_6. 17

Figure S14. ^{1}H-^{13}C HMBC spectrum of cymopolyphenol B (**2**) in DMSO-d_6. 18

Figure S15. NOESY spectrum of cymopolyphenol B (**2**) in DMSO-d_6. 19

Figure S16. ^{1}H NMR spectrum of cymopolyphenol C (**3**) in DMSO-d_6 (600 MHz). 20

Figure S17. ^{13}C NMR spectrum of cymopolyphenol C (**3**) in DMSO-d_6 (150 MHz). 21

Figure S18. DEPT135 spectrum of cymopolyphenol C (**3**) in DMSO-d_6 (150 MHz). 22

Figure S19. ^{1}H-^{1}H COSY spectrum of cymopolyphenol C (**3**) in DMSO-d_6. 23

Figure S20. ^{1}H-^{13}C HSQC spectrum of cymopolyphenol C (**3**) in DMSO-d_6. 24

Figure S21. ^{1}H-^{13}C HMBC spectrum of cymopolyphenol C (**3**) in DMSO-d_6. 25

Figure S22. NOESY spectrum of cymopolyphenol C (**3**) in DMSO-d_6. 26

Figure S23. ^{1}H NMR spectrum of cymopolyphenol D (**4**) in DMSO-d_6 (600 MHz). 27

Figure S24. ^{13}C NMR spectrum of cymopolyphenol D (**4**) in DMSO-d_6 (150 MHz). 28

Figure S25. DEPT135 spectrum of cymopolyphenol D (**4**) in DMSO-d_6 (150 MHz). 29

Figure S26. ^{1}H-^{1}H COSY spectrum of cymopolyphenol D (**4**) in DMSO-d_6. 30

Figure S27. ^{1}H-^{13}C HSQC spectrum of cymopolyphenol D (**4**) in DMSO-d_6. 31

Figure S28. ^{1}H-^{13}C HMBC spectrum of cymopolyphenol D (**4**) in DMSO-d_6. 32

Figure S29. NOESY spectrum of cymopolyphenol D (**4**) in DMSO-d_6. 33
Figure S30. ^{1}H NMR spectrum of cymopolyphenol E (**5**) in DMSO-d_6 (600 MHz). 34
Figure S31. ^{13}C NMR spectrum of cymopolyphenol E (**5**) in DMSO-d_6 (150 MHz). 35
Figure S32. DEPT135 spectrum of cymopolyphenol E (**5**) in DMSO-d_6 (150 MHz)................ 36
Figure S33. ^{1}H-^{1}H COSY spectrum of cymopolyphenol E (**5**) in DMSO-d_6. 37
Figure S34. ^{1}H-^{13}C HSQC spectrum of cymopolyphenol E (**5**) in DMSO-d_6. 38
Figure S35. ^{1}H-^{13}C HMBC spectrum of cymopolyphenol E (**5**) in DMSO-d_6. 39
Figure S36. NOESY spectrum of cymopolyphenol E (**5**) in DMSO-d_6...................................... 40
Figure S37. ^{1}H NMR spectrum of cymopolyphenol F (**6**) in DMSO-d_6 (500 MHz).................. 41
Figure S38. ^{13}C NMR spectrum of cymopolyphenol F (**6**) in DMSO-d_6 (125 MHz)................. 42
Figure S39. DEPT135 spectrum of cymopolyphenol F (**6**) in DMSO-d_6 (125 MHz)................ 43
Figure S40. ^{1}H-^{1}H COSY spectrum of cymopolyphenol F (**6**) in DMSO-d_6.............................. 44
Figure S41. ^{1}H-^{13}C HSQC spectrum of cymopolyphenol F (**6**) in DMSO-d_6. 45
Figure S42. ^{1}H-^{13}C HMBC spectrum of cymopolyphenol F (**6**) in DMSO-d_6. 46
Figure S43. NOESY spectrum of cymopolyphenol F (**6**) in DMSO-d_6...................................... 47

Table S1. Sponge sampling and taxonomic information.

Sample Number / Code	Sampling Depth	Identity
MGSP201801	60 m	*Crella* (*Pytheas*) sp.
MGSP201802	103 m	*Aaptos* sp.
MGSP201803	62 m	*Chalinidae* sp.
MGSP201804	63 m	*Discodermia* sp.
MGSP201805	62 m	*Dasychalina* sp.
MGSP201806	82 m	Irciniidae gen. et sp. indet.
MGSP201807	7 m	*Haliclona* (*Soestella*) sp.
MGSP201809	60 m	*Halichondriidae* sp.
MGSP201810	39 m	*Coelocarteria* sp.
MGSP201811	13 m	*Cinachyrella* sp.
MGSP201812	13 m	*Haliclona* sp.
MGSP201813	27 m	*Plakortis* sp.
MGSP201814	15 m	*Plakortis* sp.
MGSP201815	84 m	*Ircinia* sp.
MGSP201816	22 m	*Spongia* sp.
MGSP201817	76 m	Demospongiae gen. et sp. indet.
MGSP201822	66 m	*Dysidea* sp.
MGSP201823	66 m	*Halichondria* sp.
MGSP201824	76 m	*Axinellida* sp.

The ITS region of *Cymostachys* sp. NBUF082

GCGGGGCTACGAGCTTCACTCCAACCCCATGTGAACCTTATACCACACGTTGCTTCGG
CGGGAACGCCCCGGCTCCTCGGAGCCCGGACCAGGCGCCCGCCGGGGACCCCCAAA
CCCATGTTTCCTACAGTATTCTCTGAGTGGCAAACGCAAAATAAATCAAAACTTTTAA
CAACGGATCTCTTGGCTCTGGCATCGATGAAGAACGCAGCGAAATGCGATAAGTAAT
GTGAATTGCAGAATTCAGTGAATCATCGAATCTTTGAACGCACATTGCGCCCGCCAGC
ATTCTGGCGGGCATGCCTGTCCGAGCGTCATTTCAACCCTCAGGGTCCCCCGGGAAC
CTGGTGTTGGGGATCGGCCCCGCCCGCACGGGCGTCGCCGTCCCCCAAATGCAGTGG
CGGTCCCGCCGCAGCCTCCCCTGCGTAGTAGCTAACACCTCGCACCGGAGCGCGGCG
CGGCCACGCCGTAAAACCCCCGACCTCATGAACGTTGACCTCGGATCAGGTAGGAAT
ACCCGCTGAACTTAAGCATATCAATAAGCGGAGGAATTA

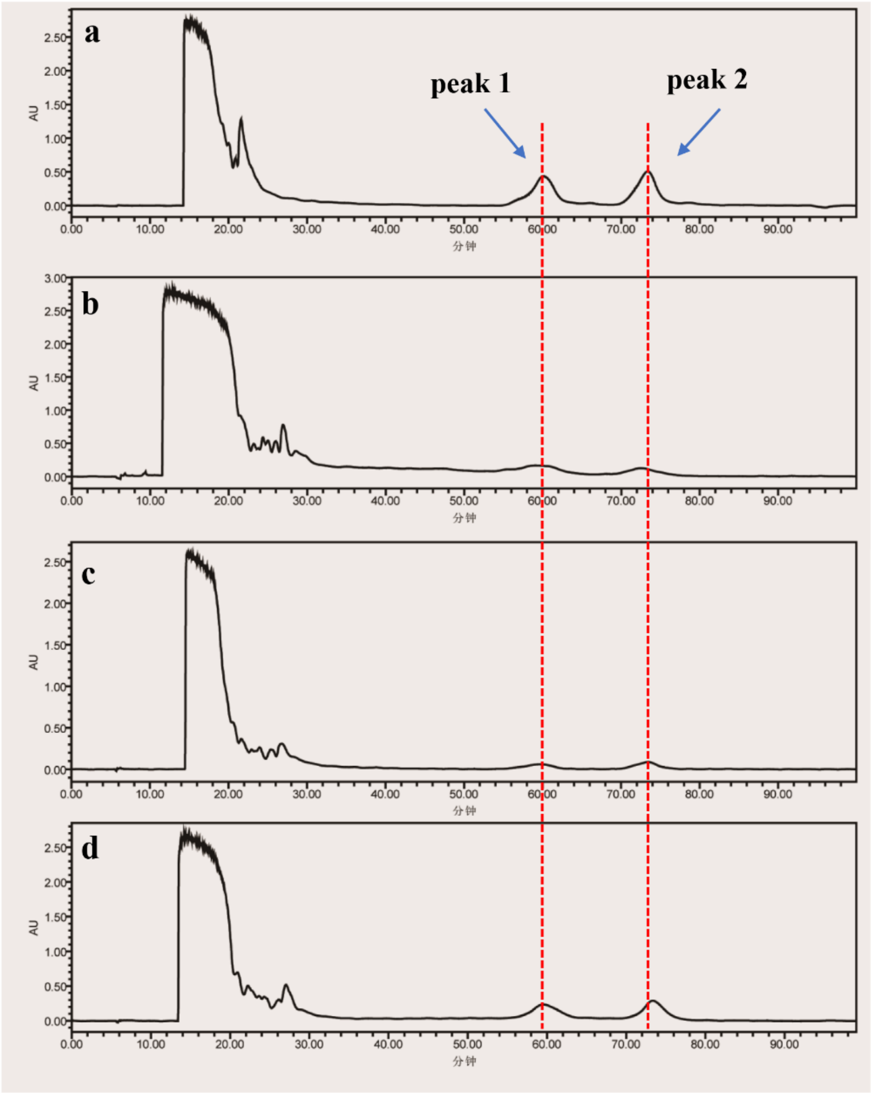

Figure S1. Chiral HPLC analysis of **5** and its separated enantiomers.

Panel a: Compound **5**.

Panel b: Re-injection of peak 1 after separation by chiral chromatography of **5**.

Panel c: Re-injection of peak 2 after separation by chiral chromatography of **5.**

Panel d: Co-injection of **5** with peaks 1 and 2 after separation by chiral chromatography of **5**.

Samples were evaluated on a Chiralomix SA column [(5 µm, 250 × 4.6 mm); Sepax Technologies (Newark, Delaware, USA)] with isocratic elution (n-hexane/ethanol, v/v, 92/8).

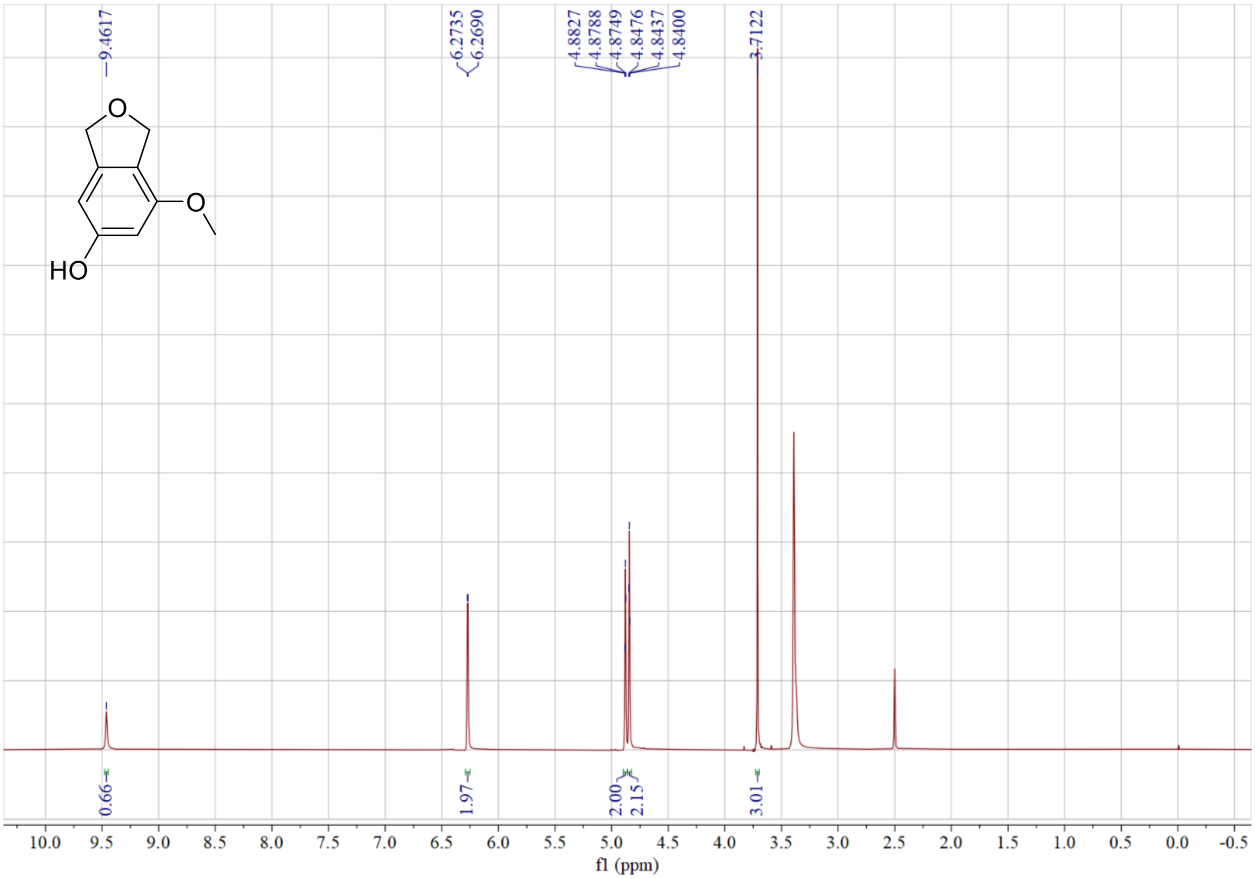

Figure S2. ^{1}H NMR spectrum of cymopolyphenol A (**1**) in DMSO-d_6 (600 MHz).

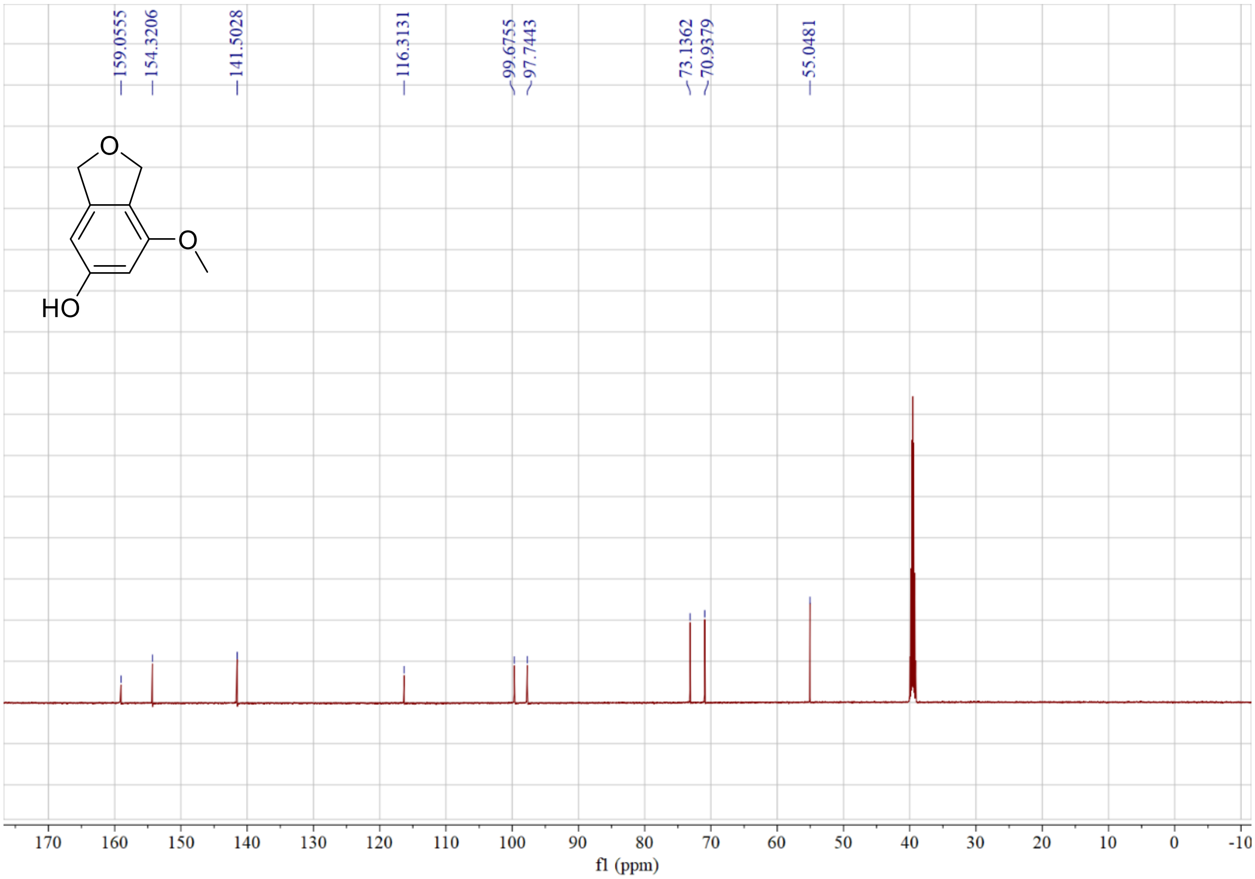

Figure S3. ^{13}C NMR spectrum of cymopolyphenol A (**1**) in DMSO-*d*$_6$ (150 MHz).

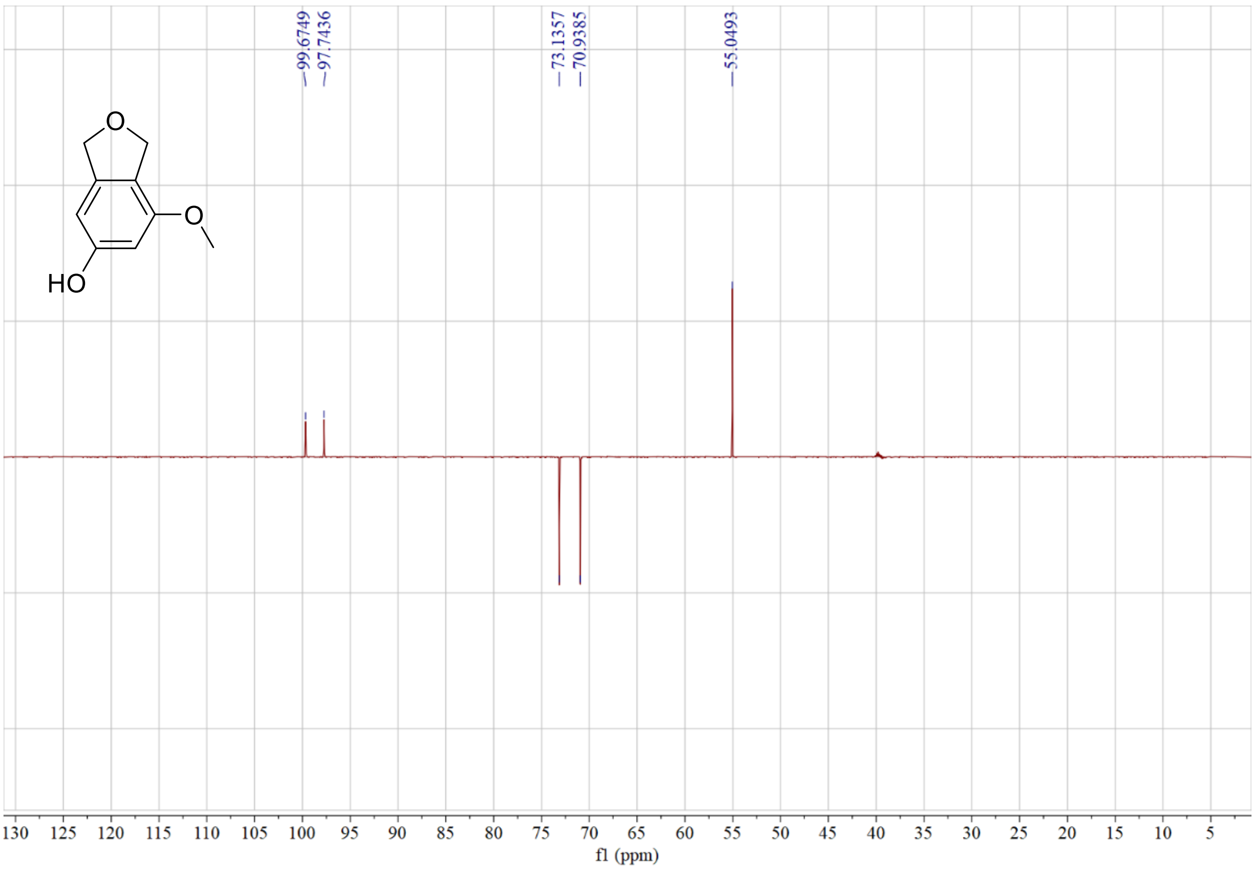

Figure S4. DEPT135 spectrum of cymopolyphenol A (**1**) in DMSO-d_6 (150 MHz).

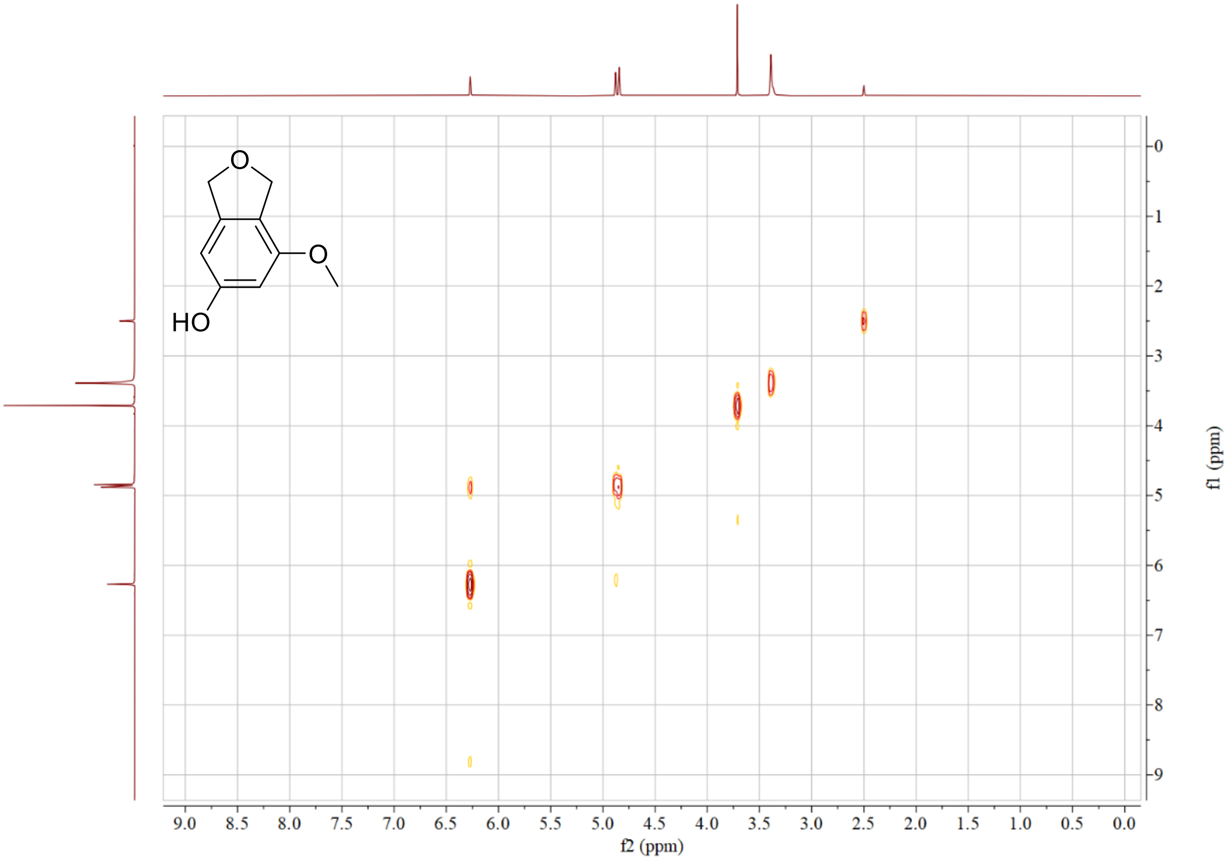

Figure S5. ^{1}H-^{1}H COSY spectrum of cymopolyphenol A (**1**) in DMSO-d_6.

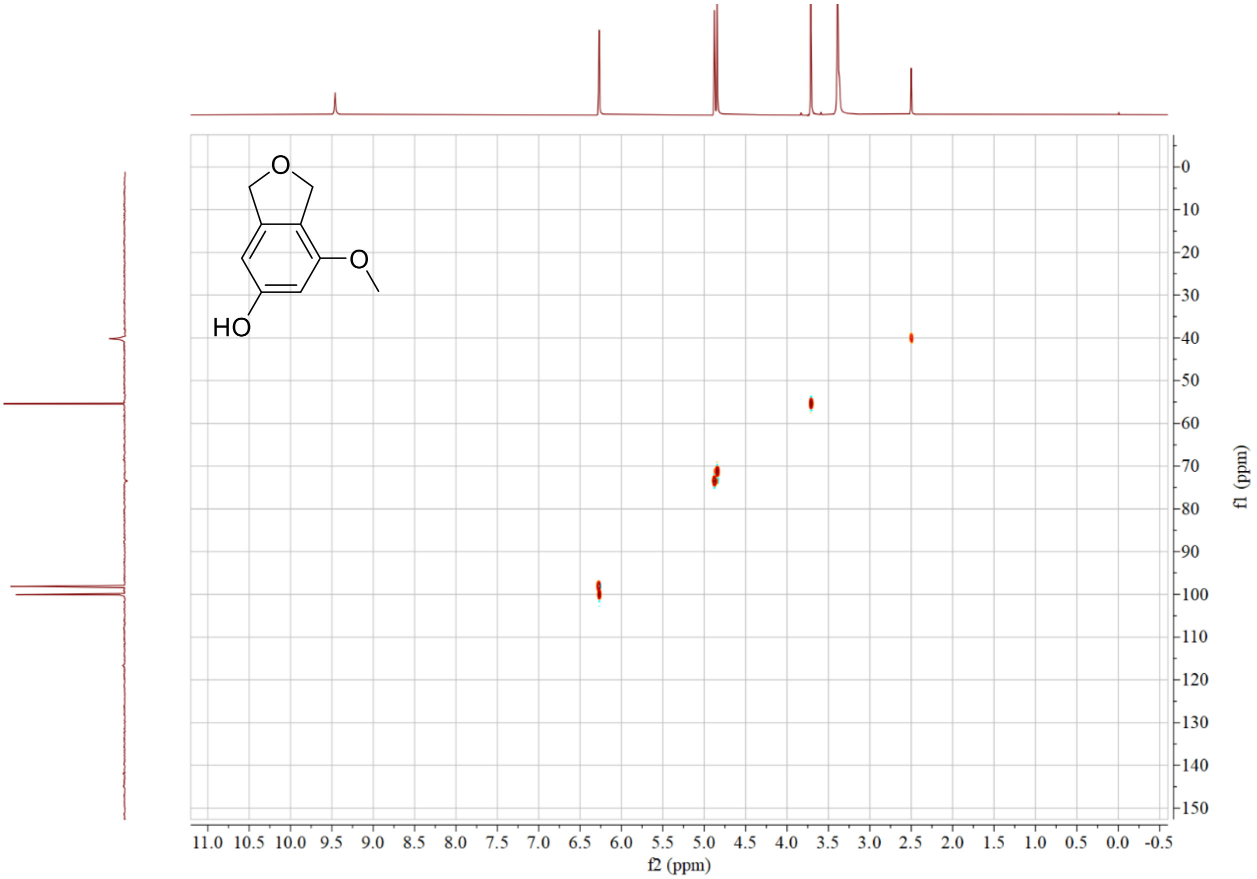

Figure S6. ^{1}H-^{13}C HSQC spectrum of cymopolyphenol A (**1**) in DMSO-*d*6.

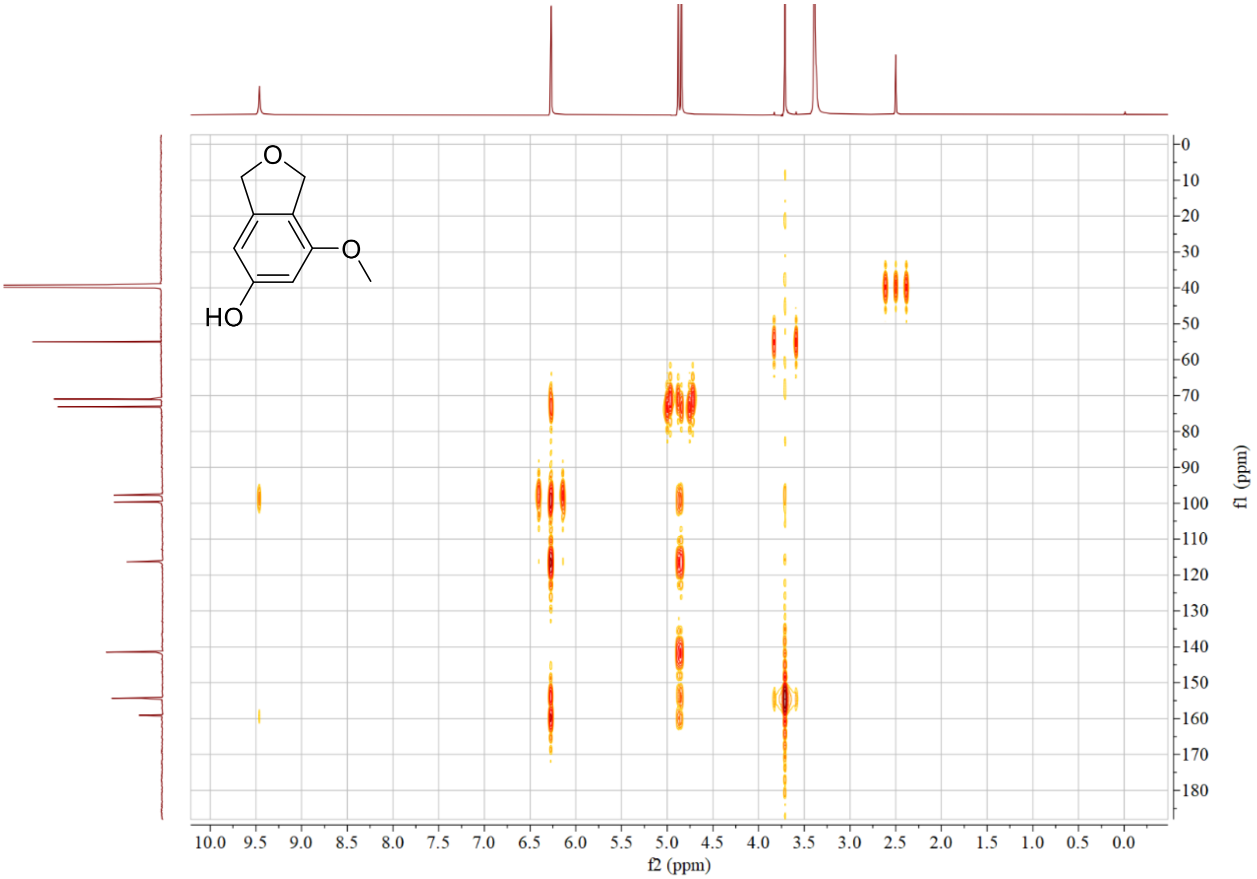

Figure S7. ^{1}H-^{13}C HMBC spectrum of cymopolyphenol A (**1**) in DMSO-d_6.

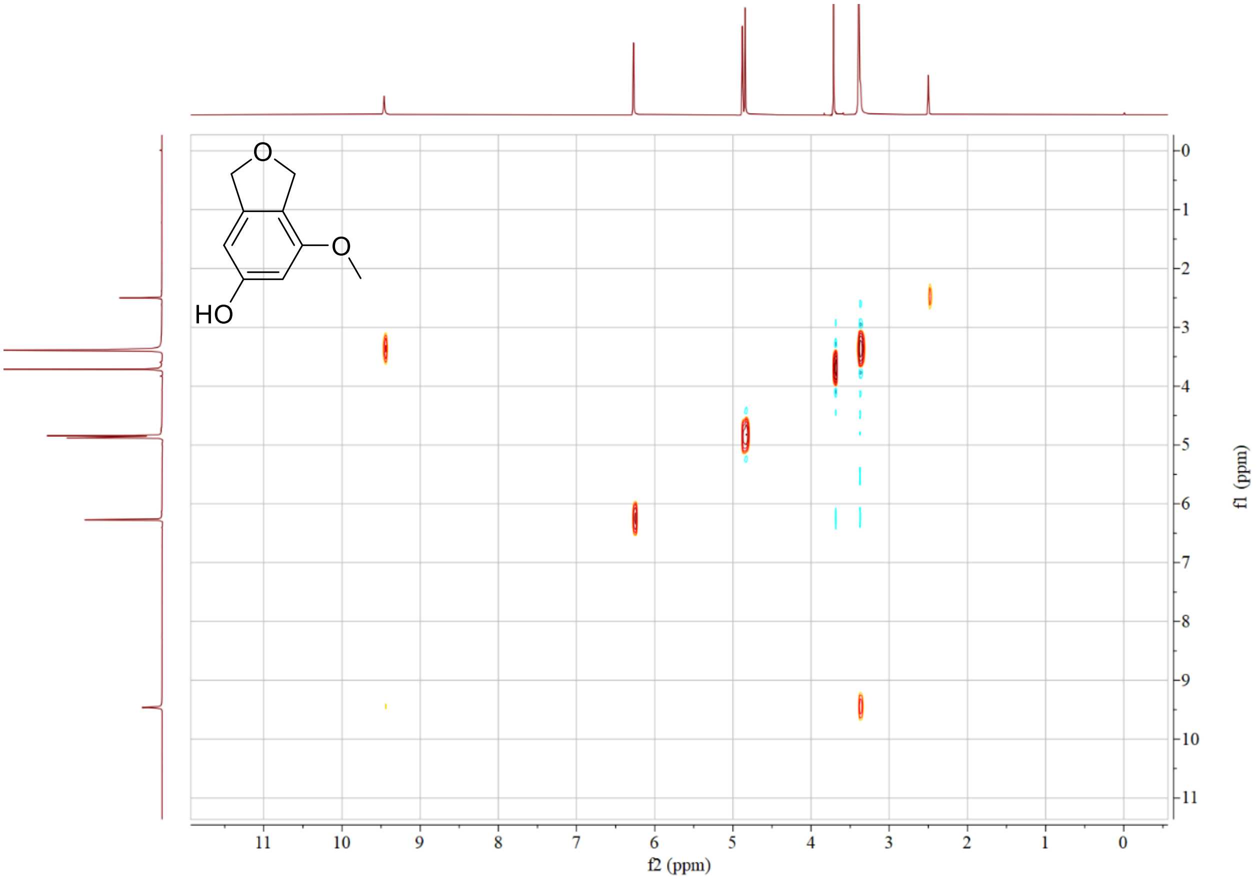

Figure S8. NOESY spectrum of cymopolyphenol A (**1**) in DMSO-d_6.

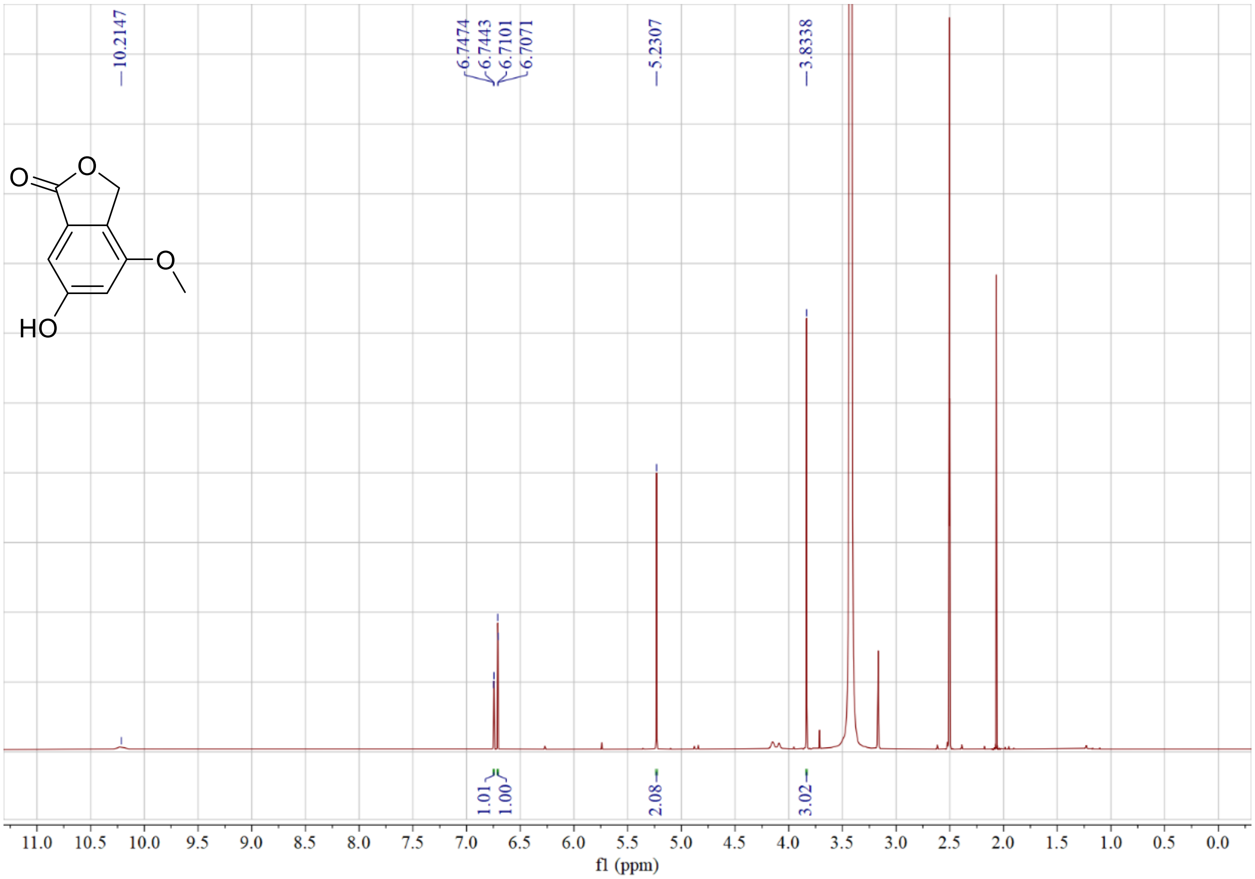

Figure S9. ^{1}H NMR spectrum of cymopolyphenol B (**2**) in DMSO-d_6 (600 MHz).

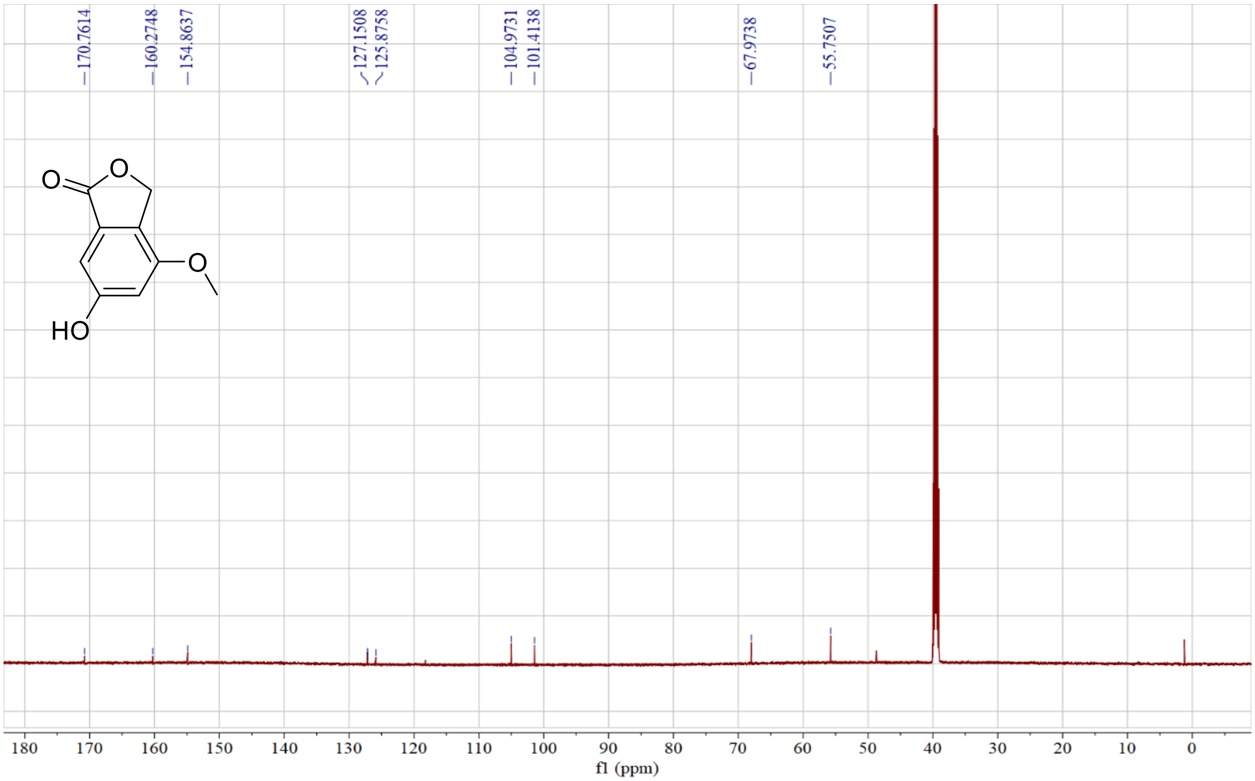

Figure S10. ^{13}C NMR spectrum of cymopolyphenol B (**2**) in DMSO-d_6 (150 MHz).

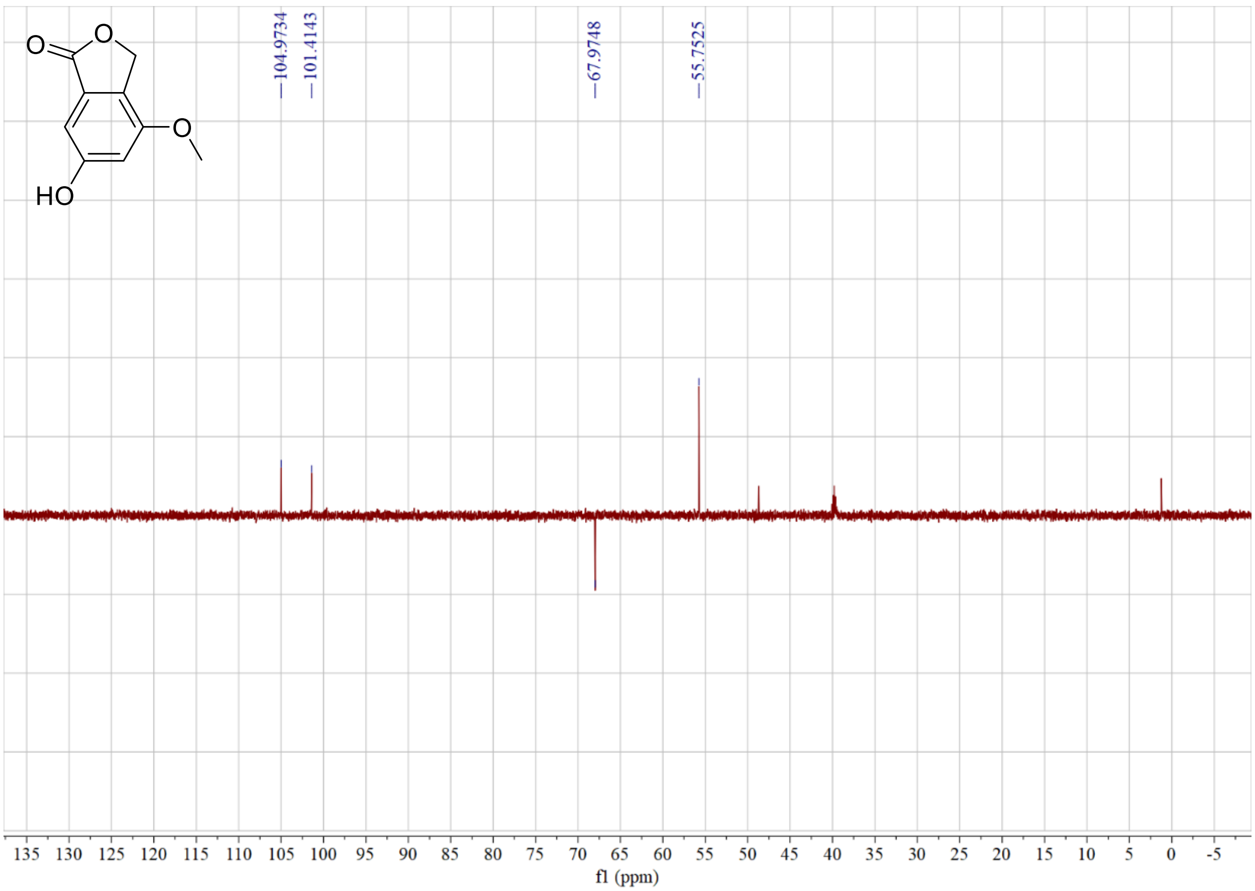

Figure S11. DEPT135 spectrum of cymopolyphenol B (**2**) in DMSO-d_6 (150 MHz).

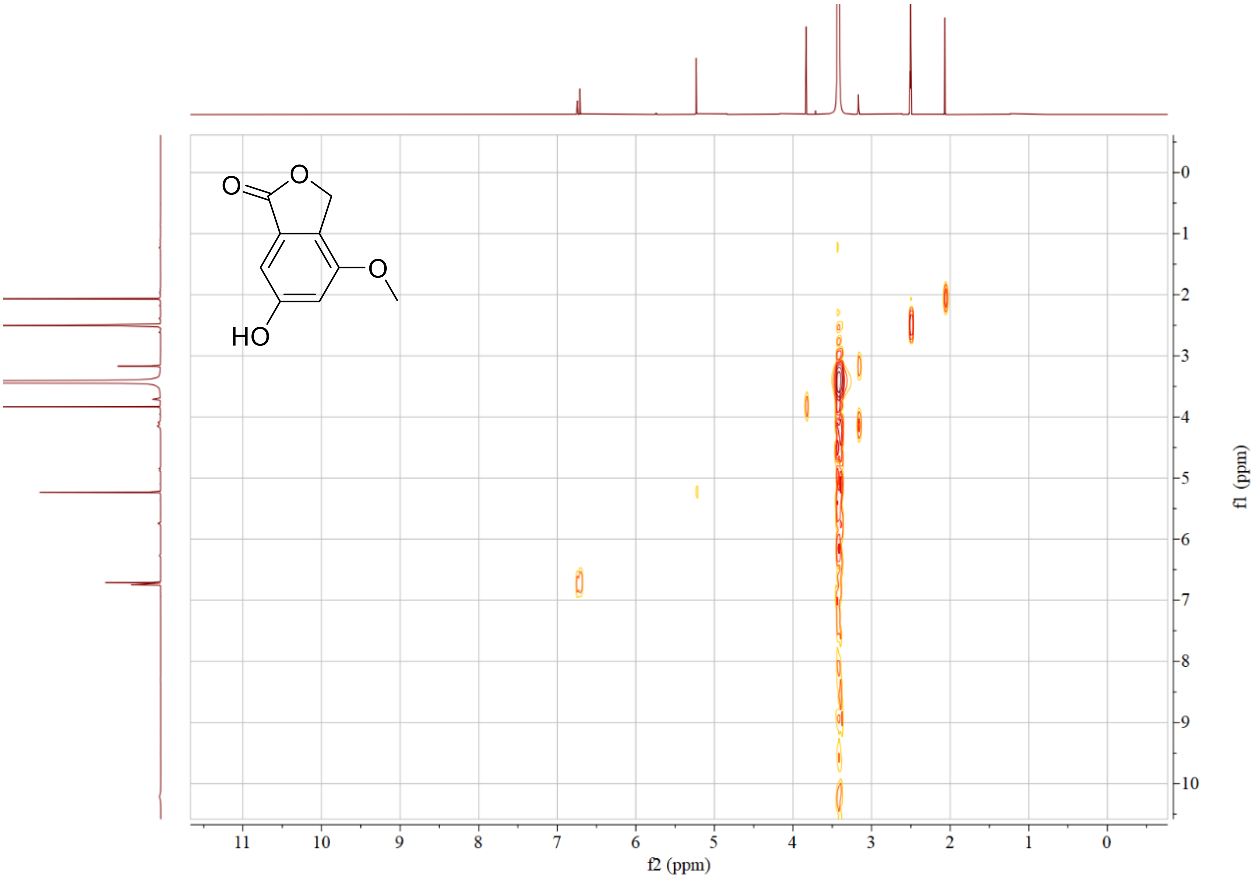

Figure S12. ^{1}H-^{1}H COSY spectrum of cymopolyphenol B (**2**) in DMSO-d_6.

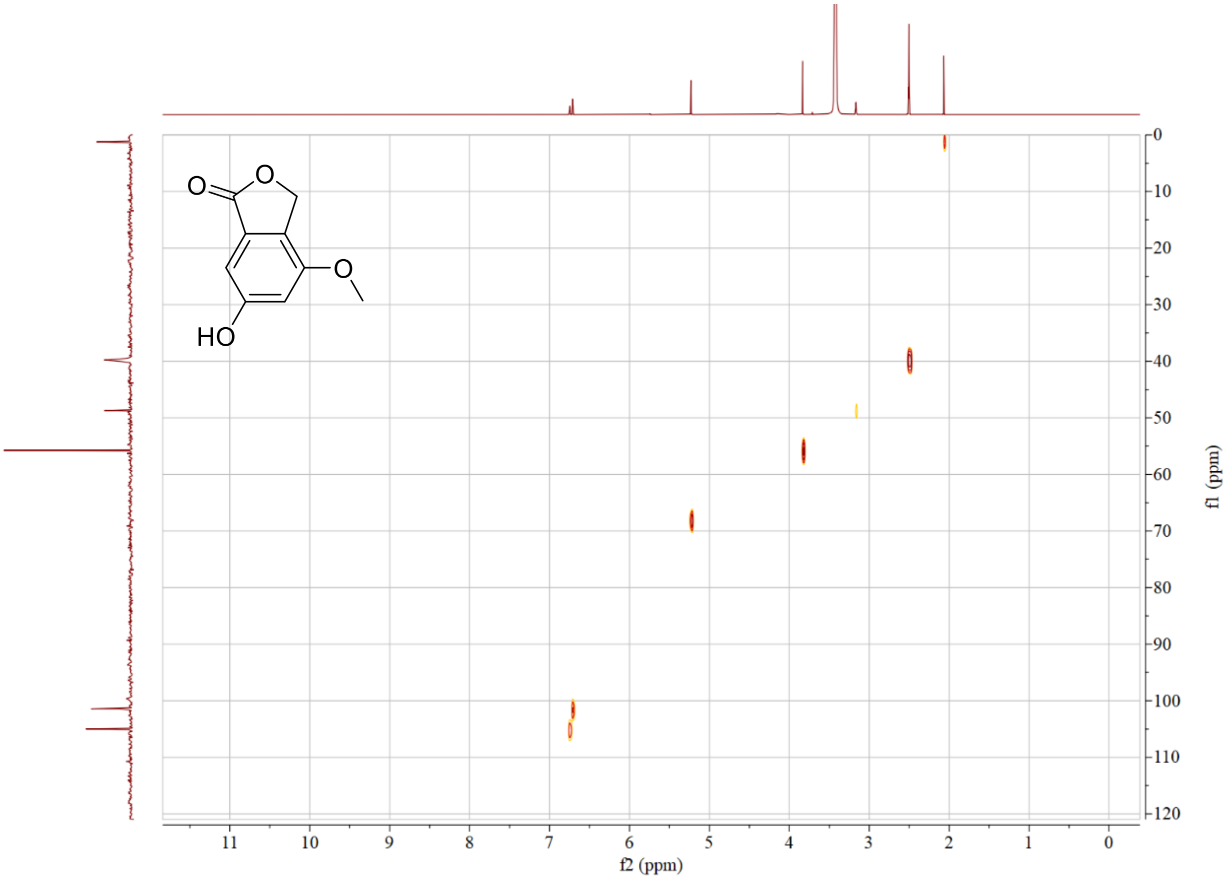

Figure S13. ^{1}H-^{13}C HSQC spectrum of cymopolyphenol B (**2**) in DMSO-d_6.

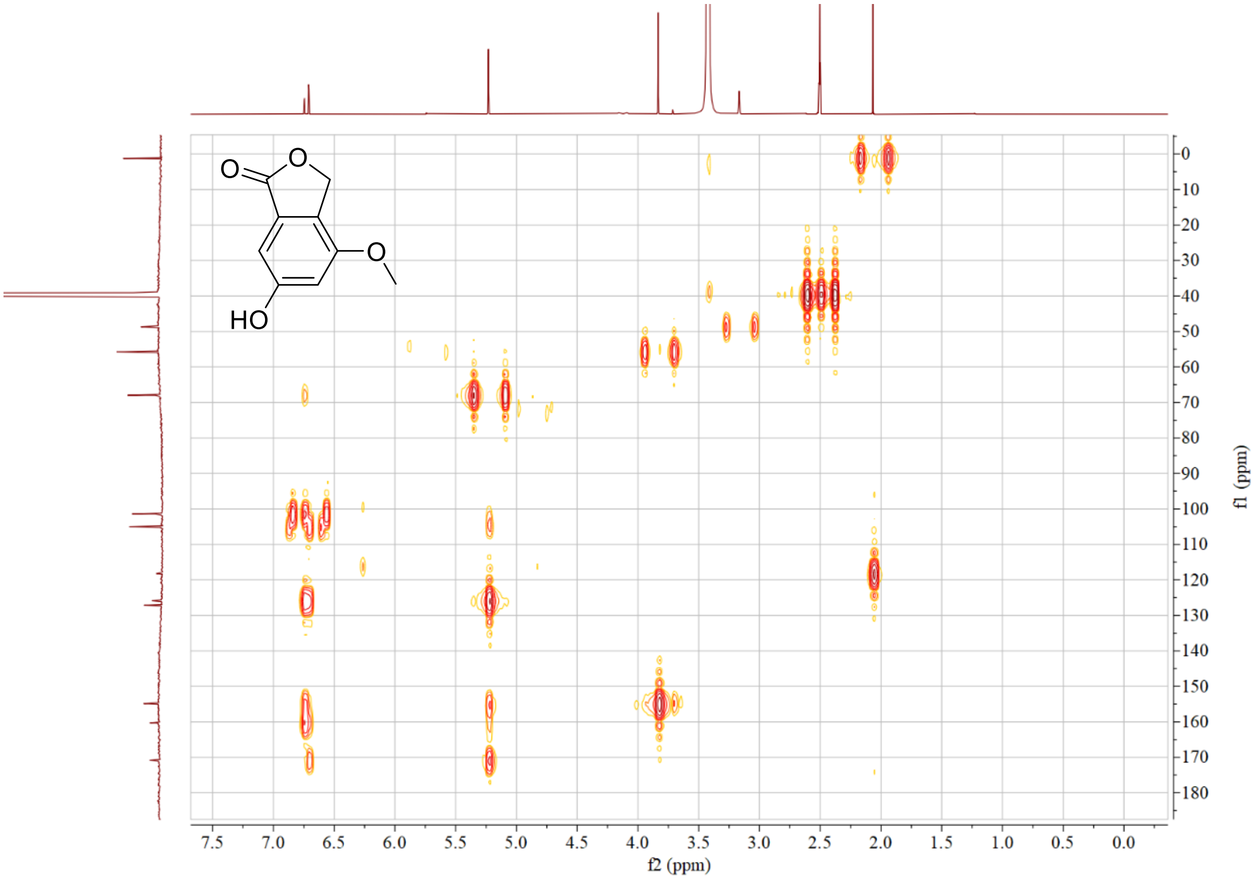

Figure S14. ^{1}H-^{13}C HMBC spectrum of cymopolyphenol B (**2**) in DMSO-d_6.

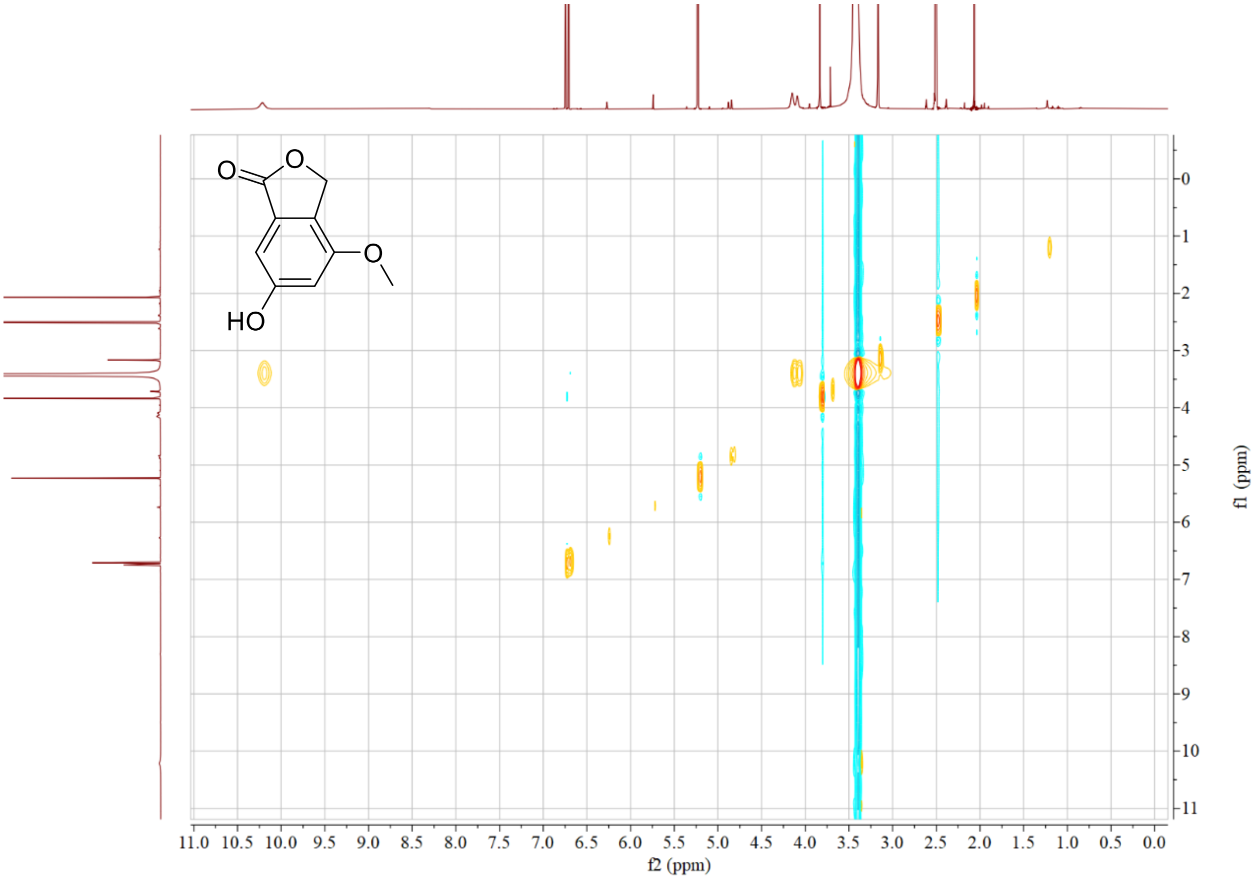

Figure S15. NOESY spectrum of cymopolyphenol B (**2**) in DMSO-d_6.

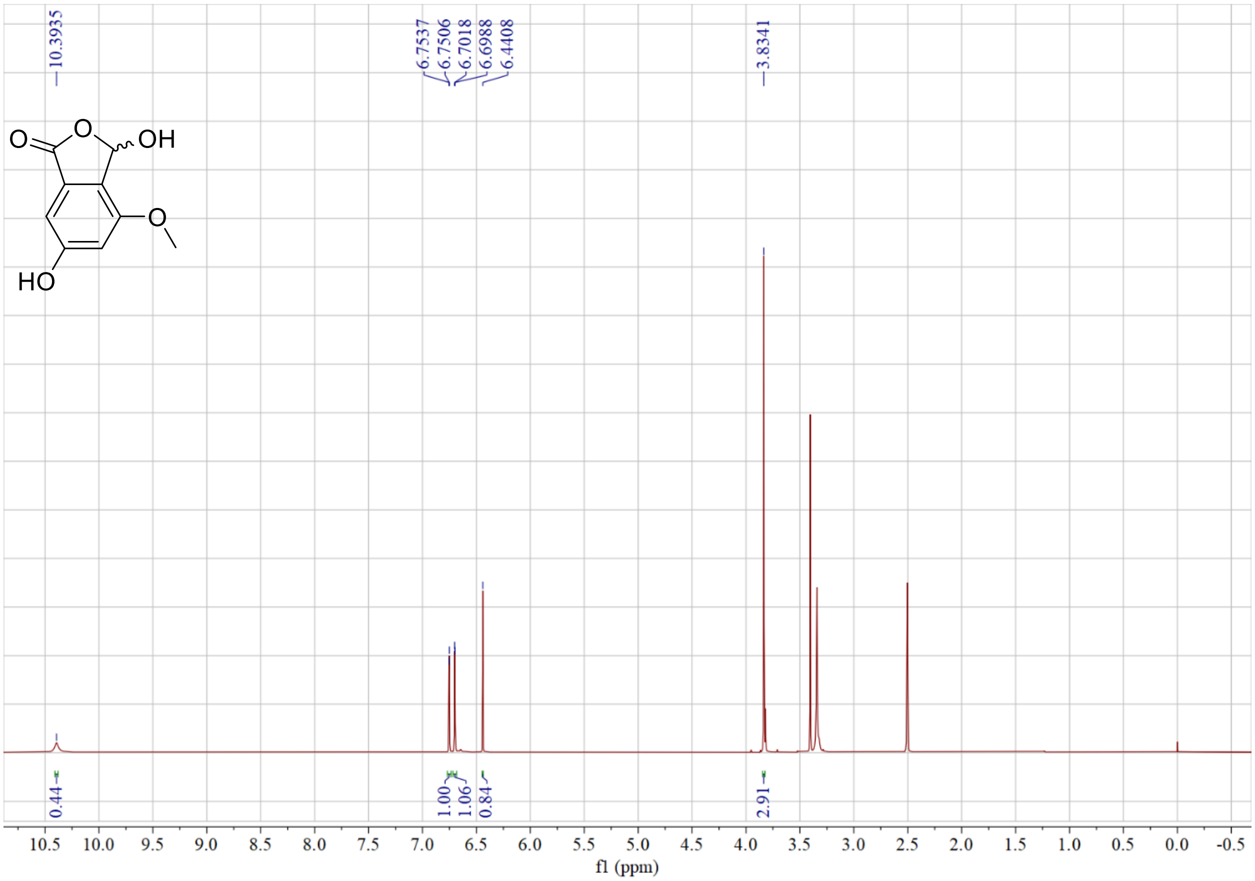

Figure S16. ^{1}H NMR spectrum of cymopolyphenol C (**3**) in DMSO-d_6 (600 MHz).

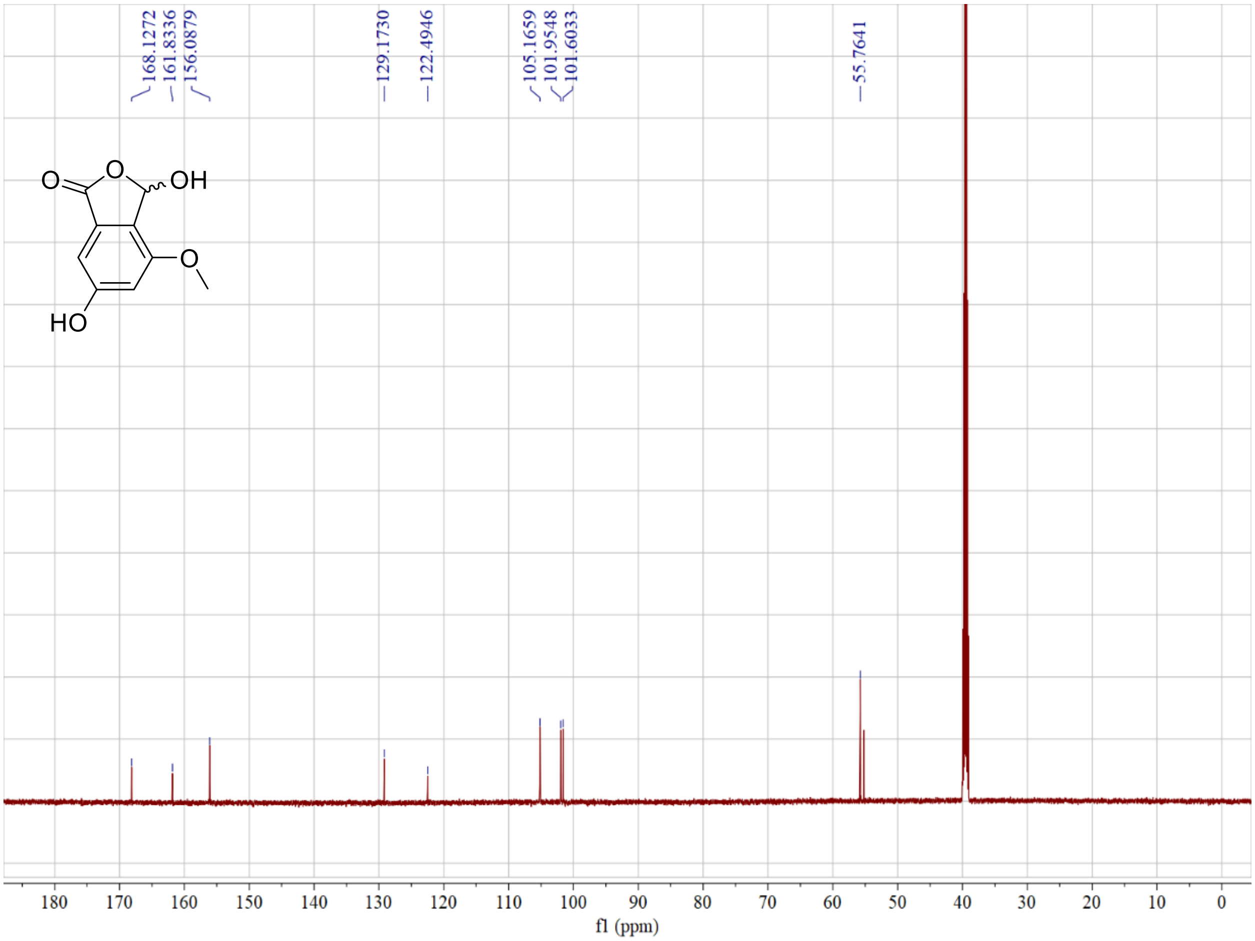

Figure S17. ^{13}C NMR spectrum of cymopolyphenol C (**3**) in DMSO-d_6 (150 MHz).

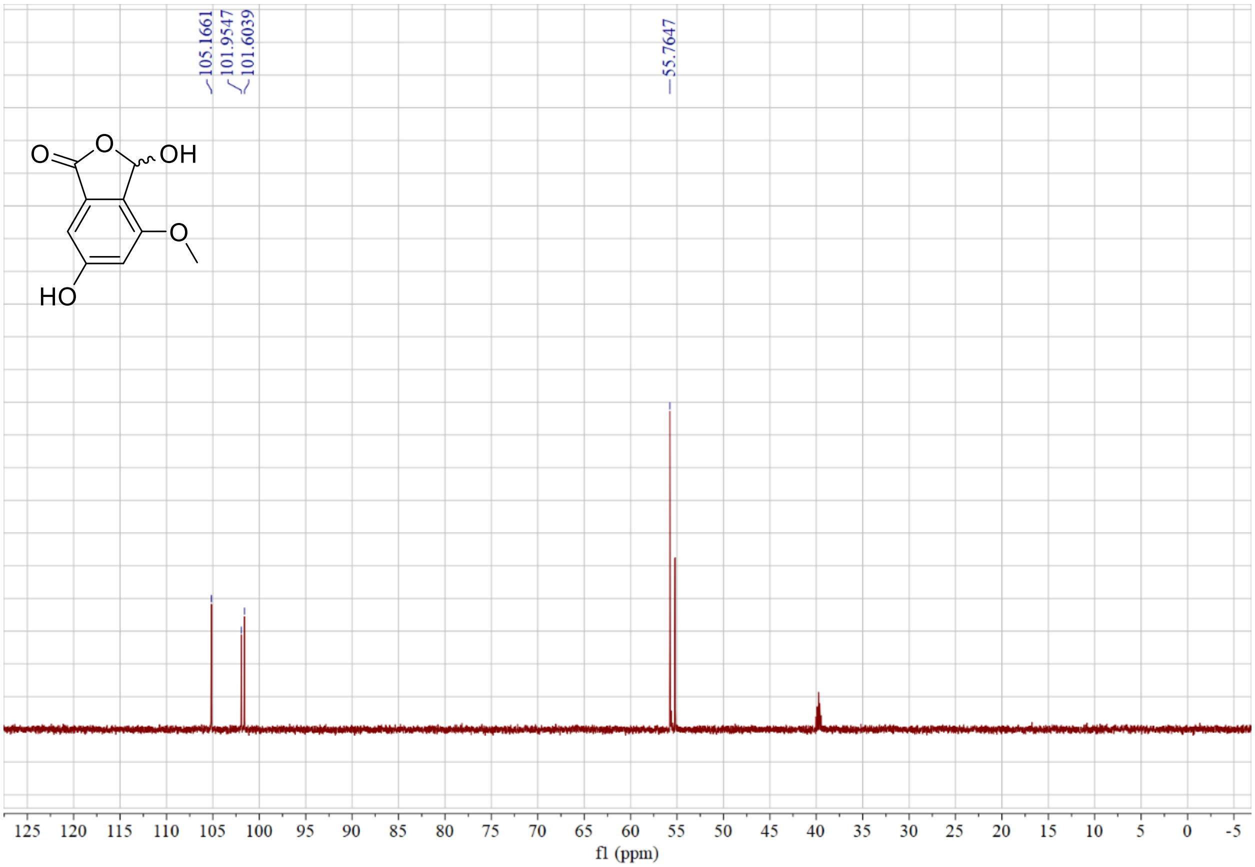

Figure S18. DEPT135 spectrum of cymopolyphenol C (**3**) in DMSO-d_6 (150 MHz).

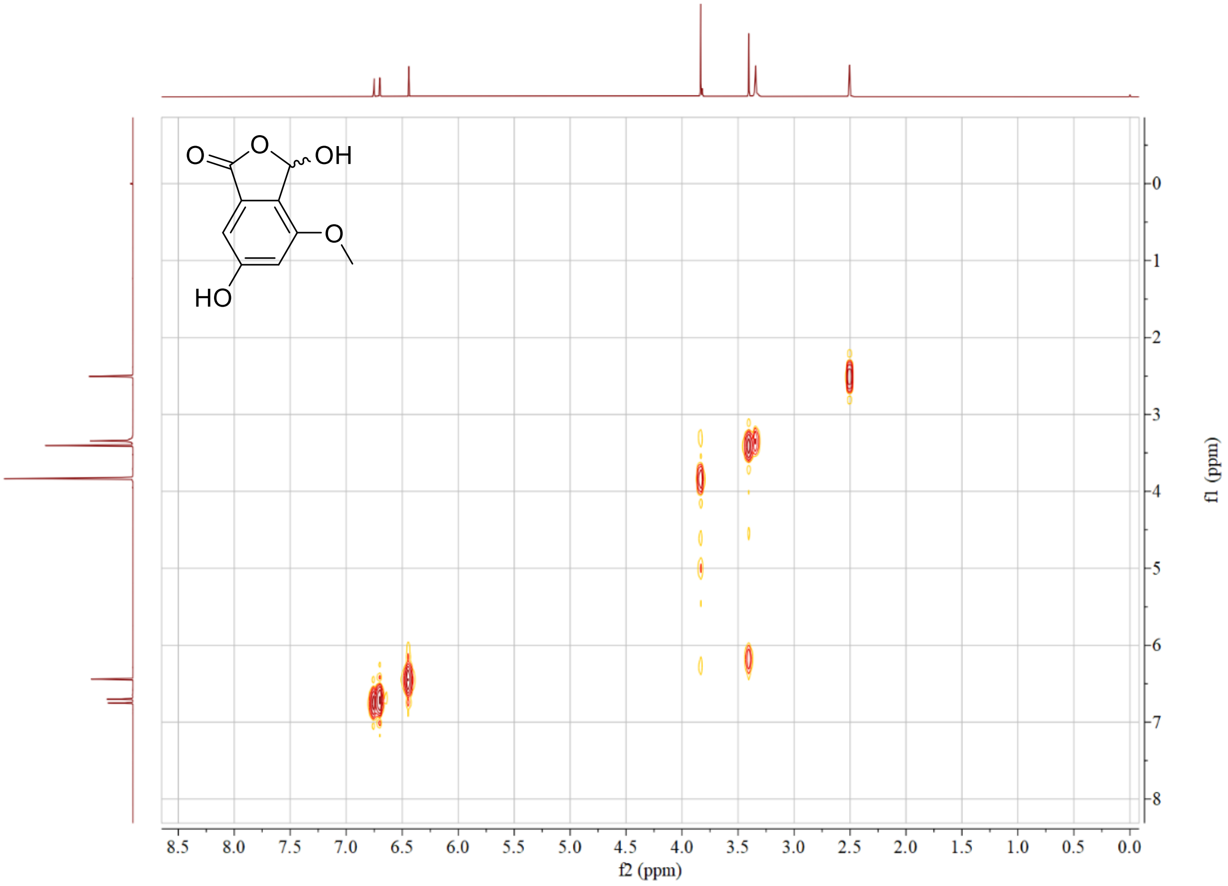

Figure S19. ^1H-^1H COSY spectrum of cymopolyphenol C (**3**) in DMSO-d_6.

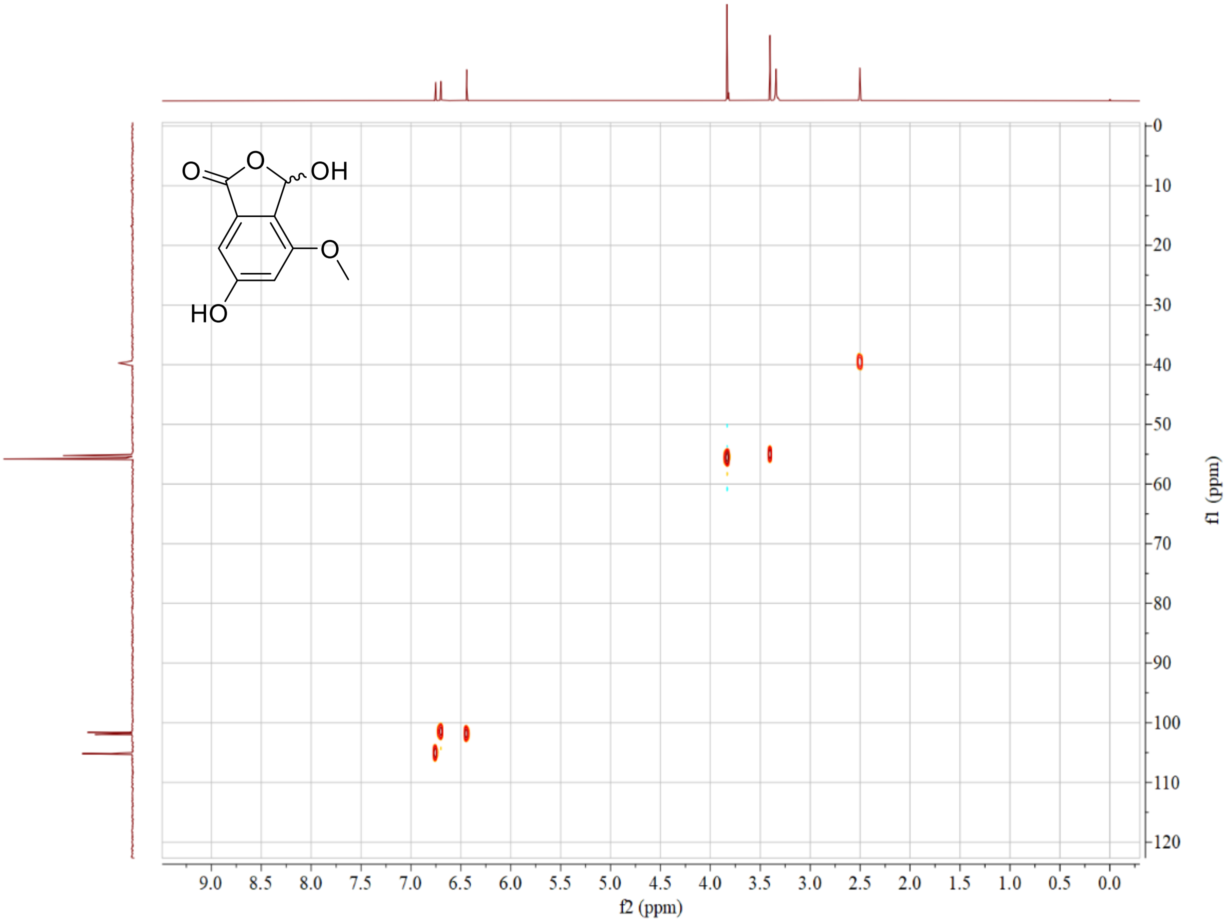

Figure S20. ^{1}H-^{13}C HSQC spectrum of cymopolyphenol C (**3**) in DMSO-d_6.

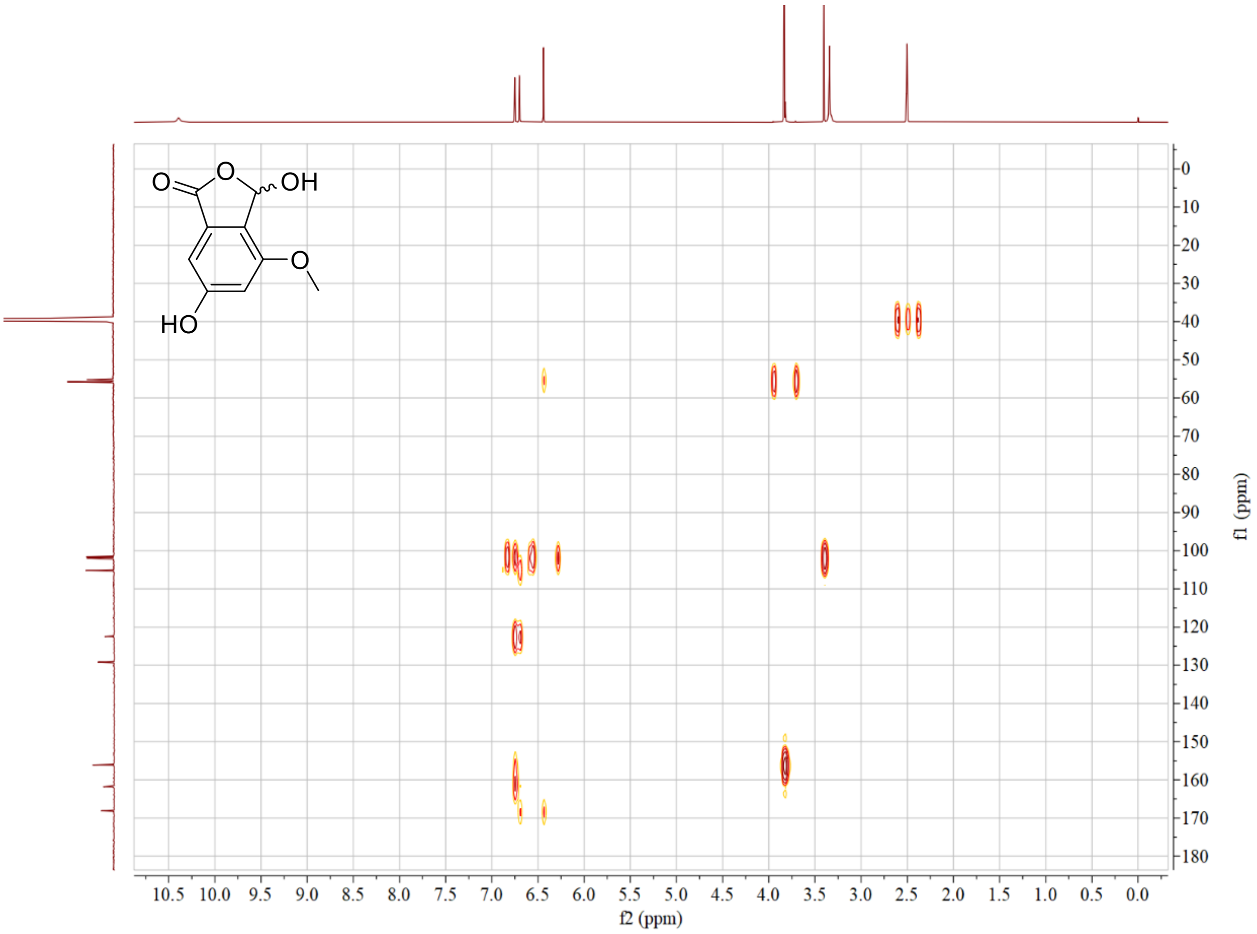

Figure S21. ^{1}H-^{13}C HMBC spectrum of cymopolyphenol C (**3**) in DMSO-d_6.

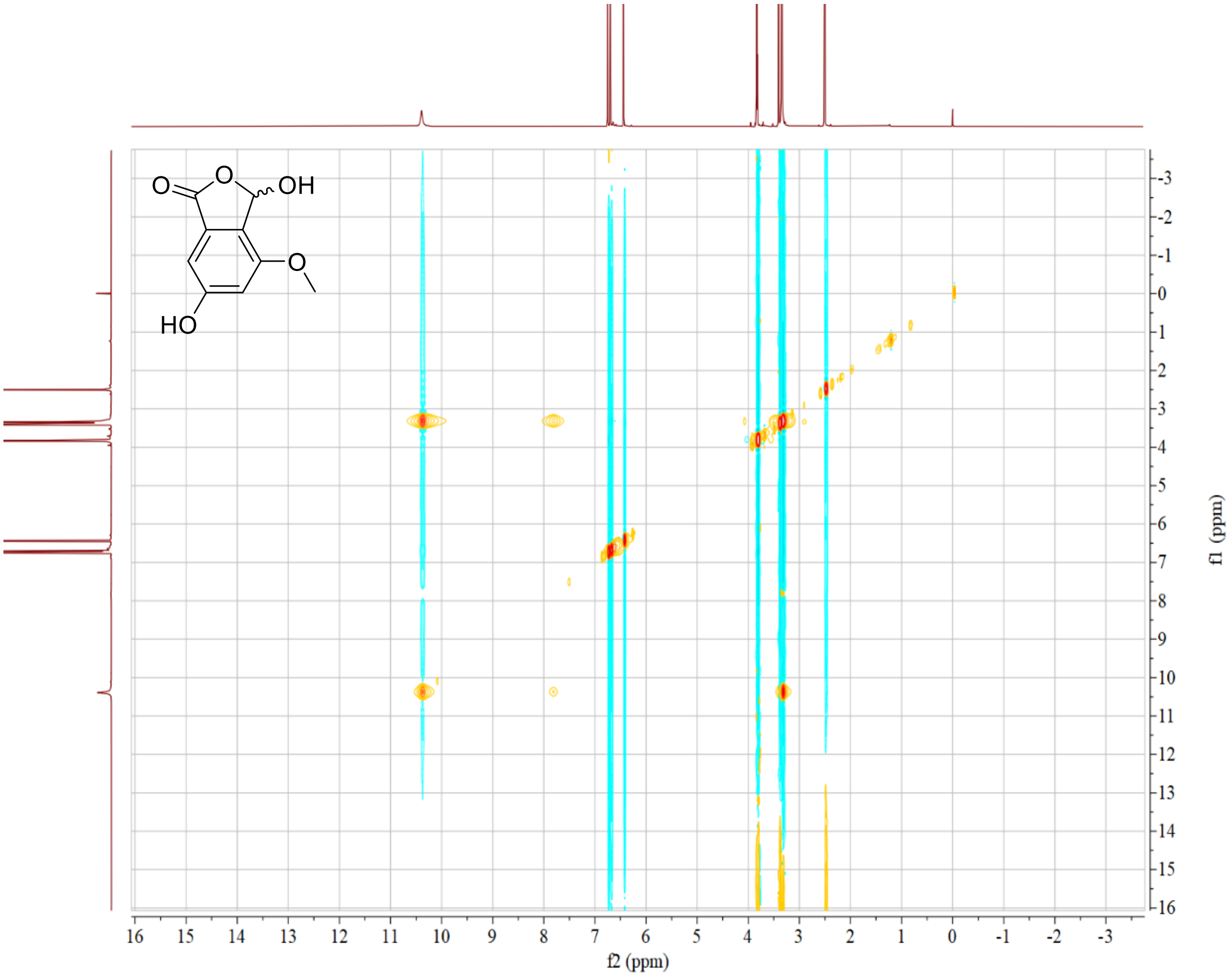

Figure S22. NOESY spectrum of cymopolyphenol C (**3**) in DMSO-d_6.

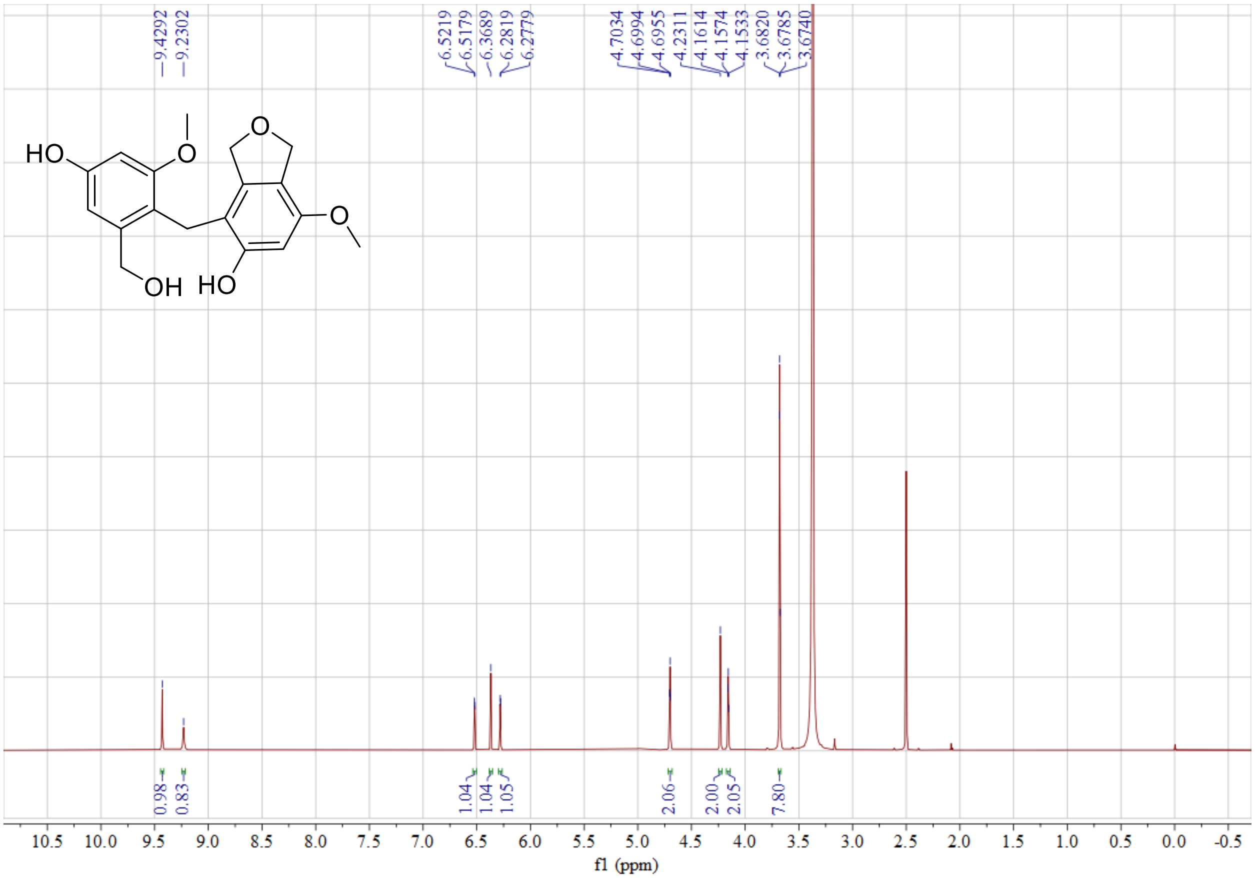

Figure S23. ^{1}H NMR spectrum of cymopolyphenol D (**4**) in DMSO-d_6 (600 MHz).

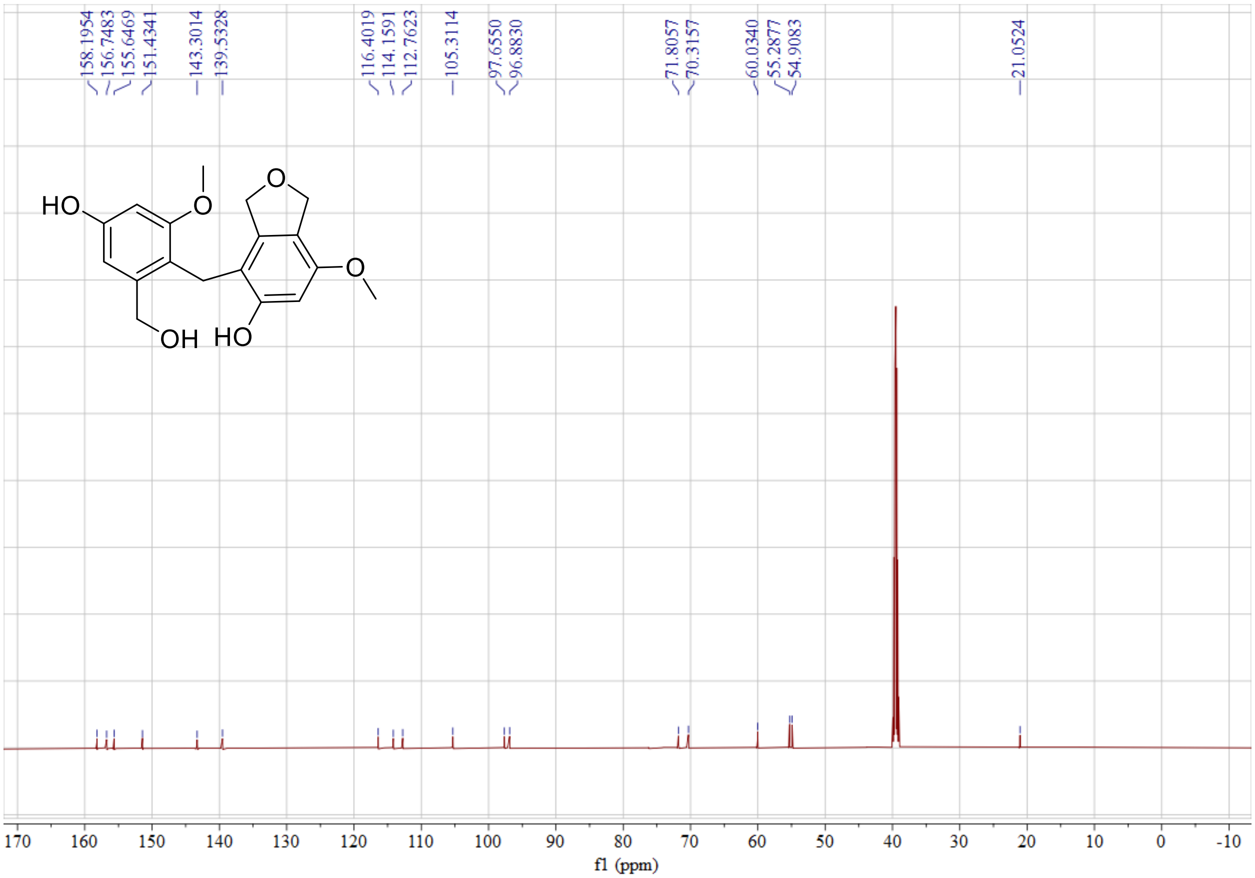

Figure S24. ^{13}C NMR spectrum of cymopolyphenol D (**4**) in DMSO-d_6 (150 MHz).

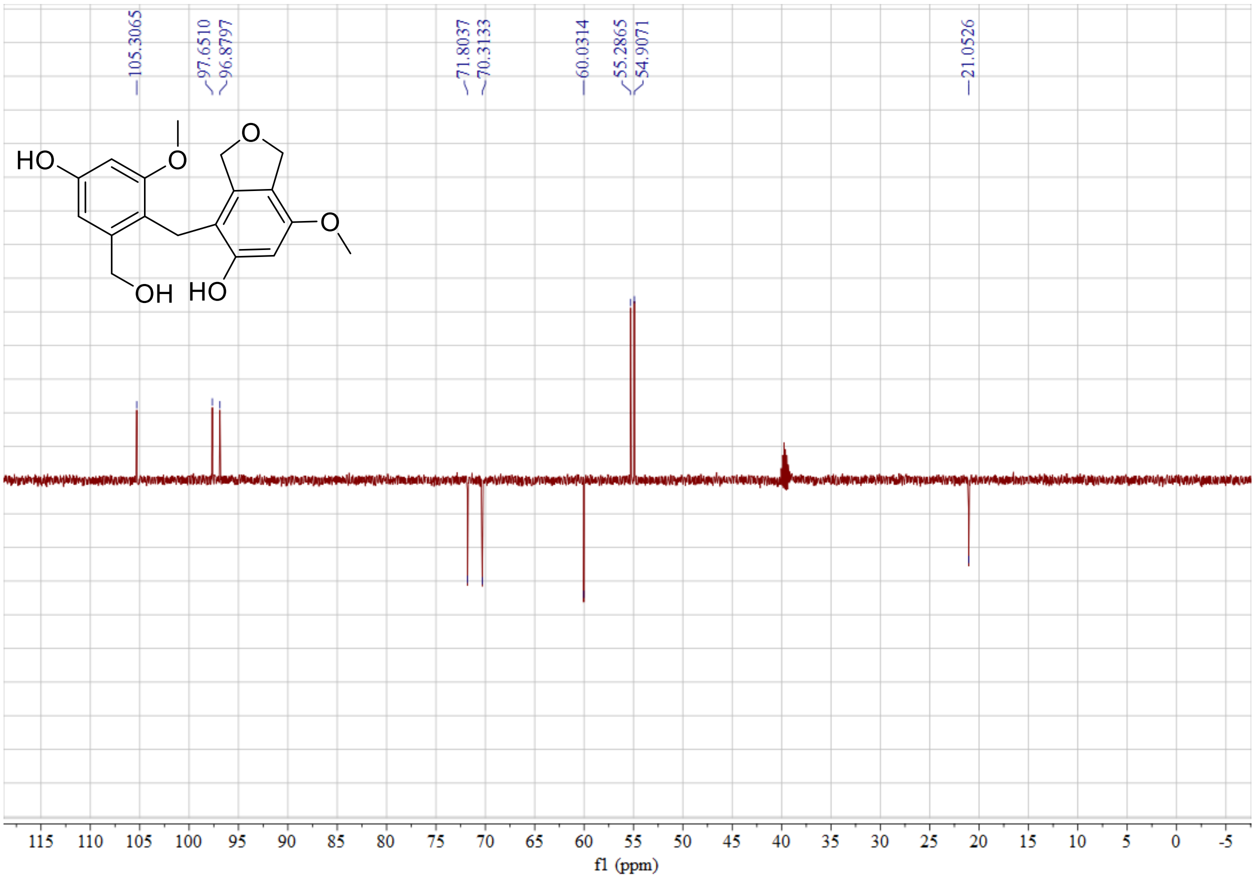

Figure S25. DEPT135 spectrum of cymopolyphenol D (**4**) in DMSO-d_6 (150 MHz).

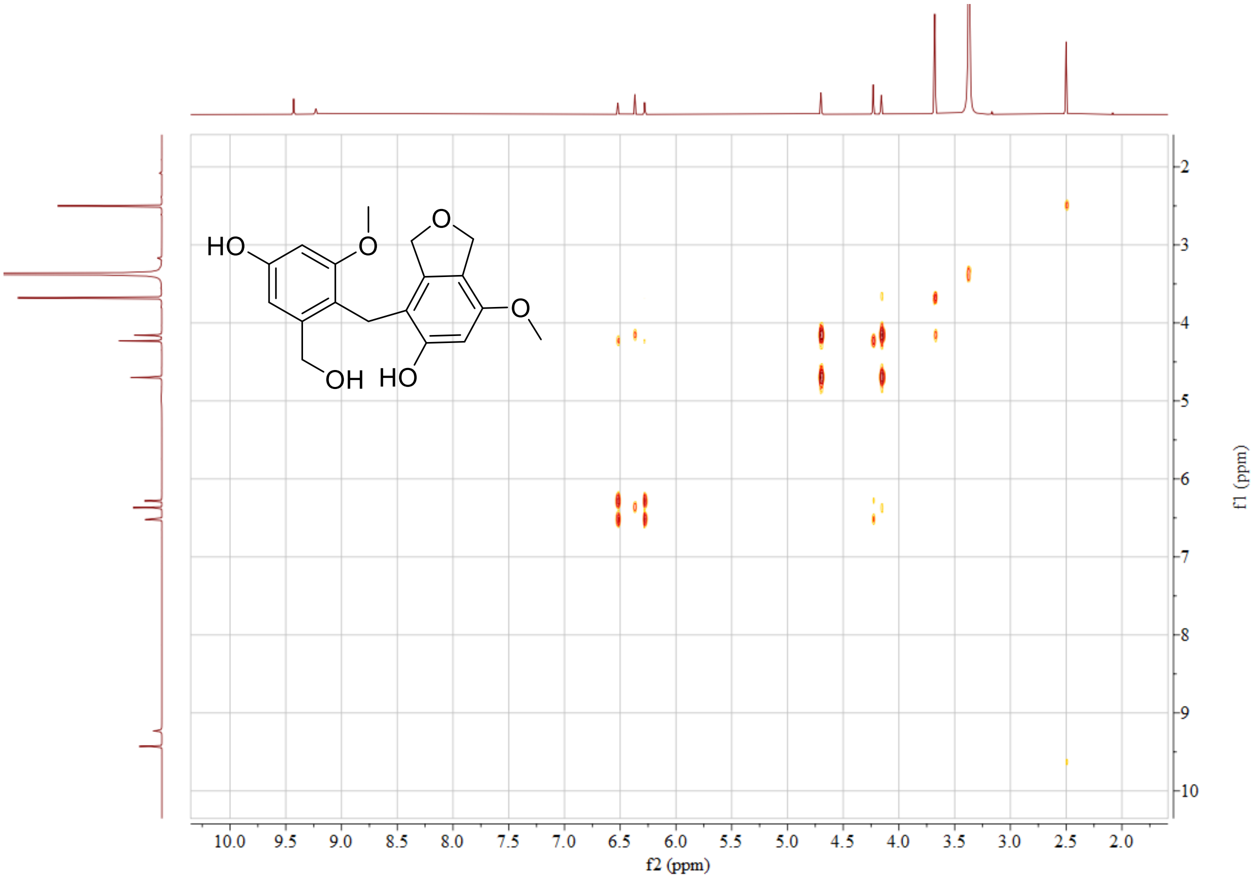

Figure S26. ^{1}H-^{1}H COSY spectrum of cymopolyphenol D (**4**) in DMSO-d_6.

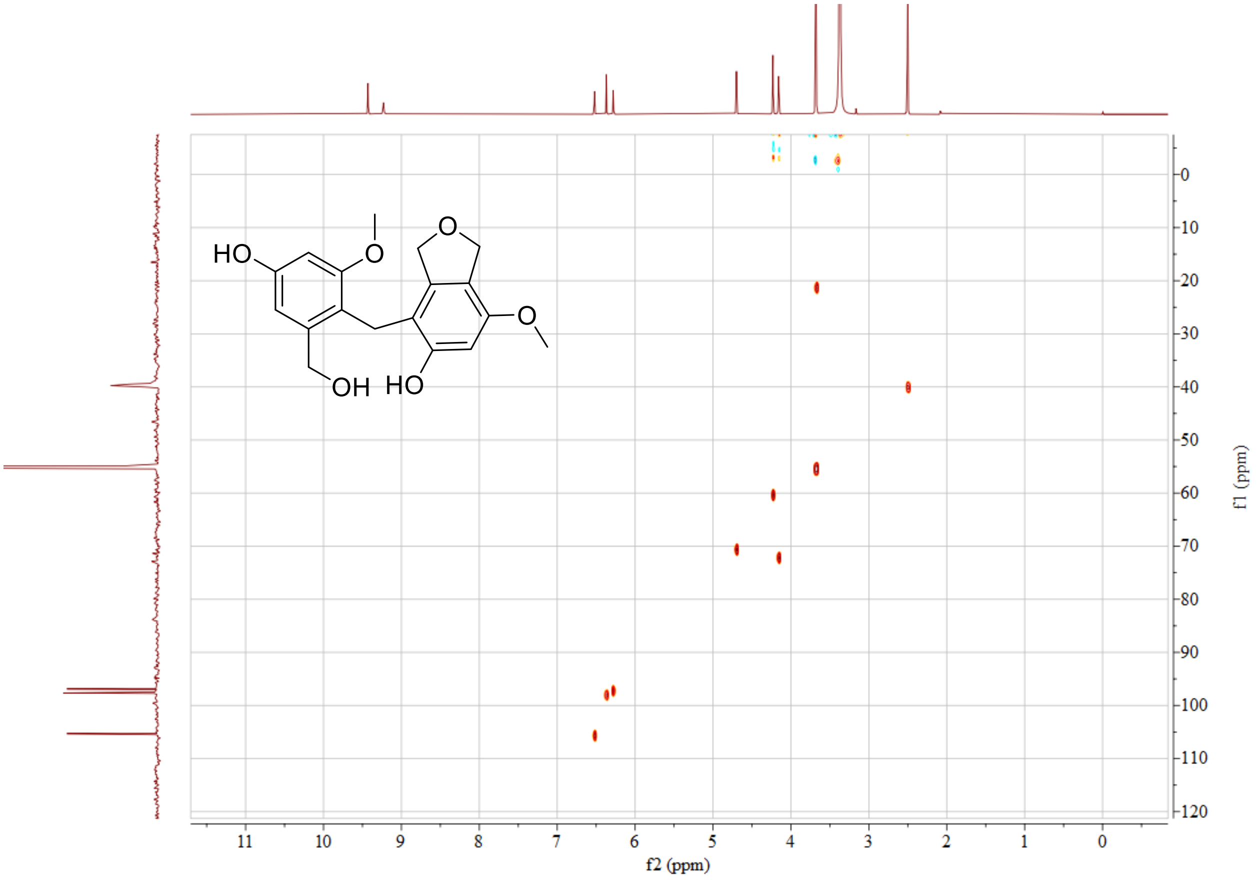

Figure S27. ^{1}H-^{13}C HSQC spectrum of cymopolyphenol D (**4**) in DMSO-d_6.

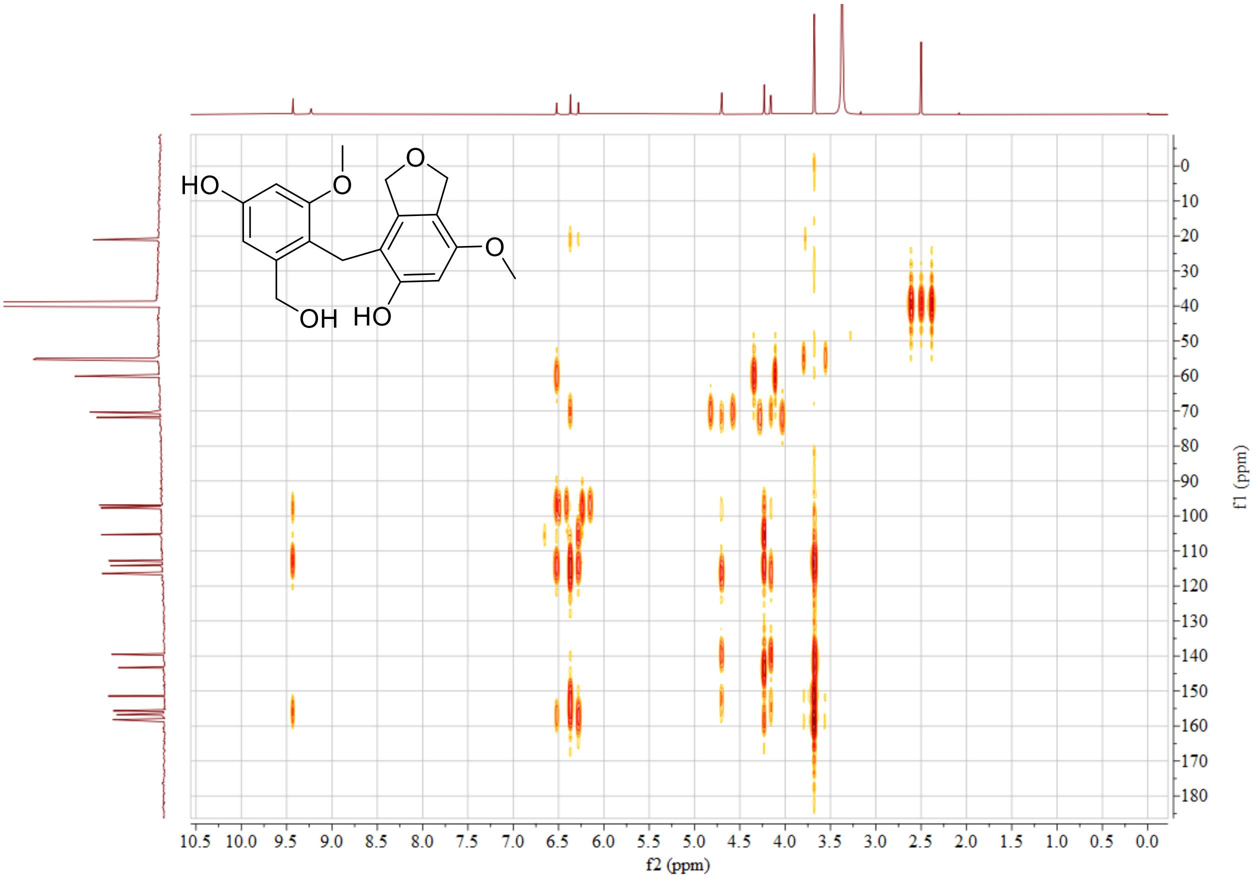

Figure S28. ^{1}H-^{13}C HMBC spectrum of cymopolyphenol D (**4**) in DMSO-d_6.

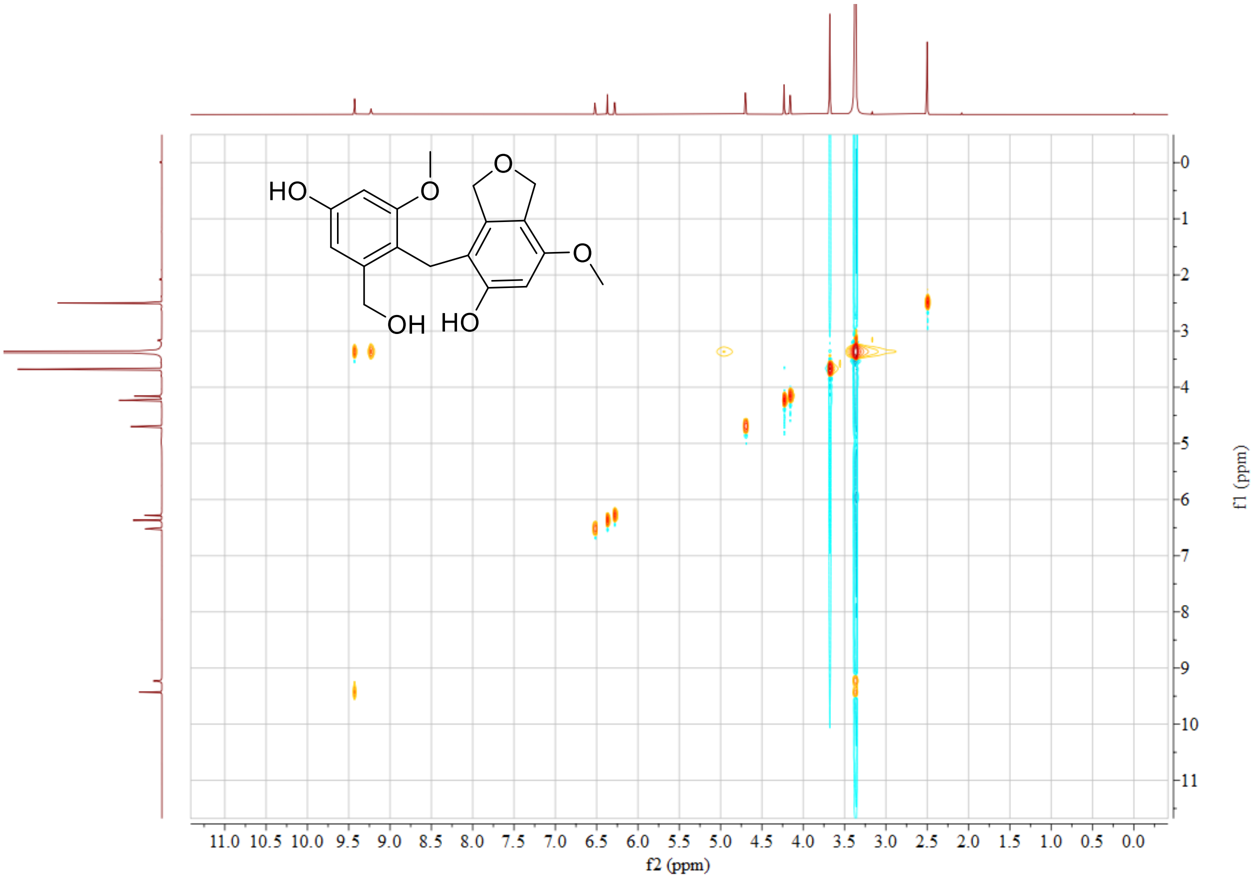

Figure S29. NOESY spectrum of cymopolyphenol D (**4**) in DMSO-d_6.

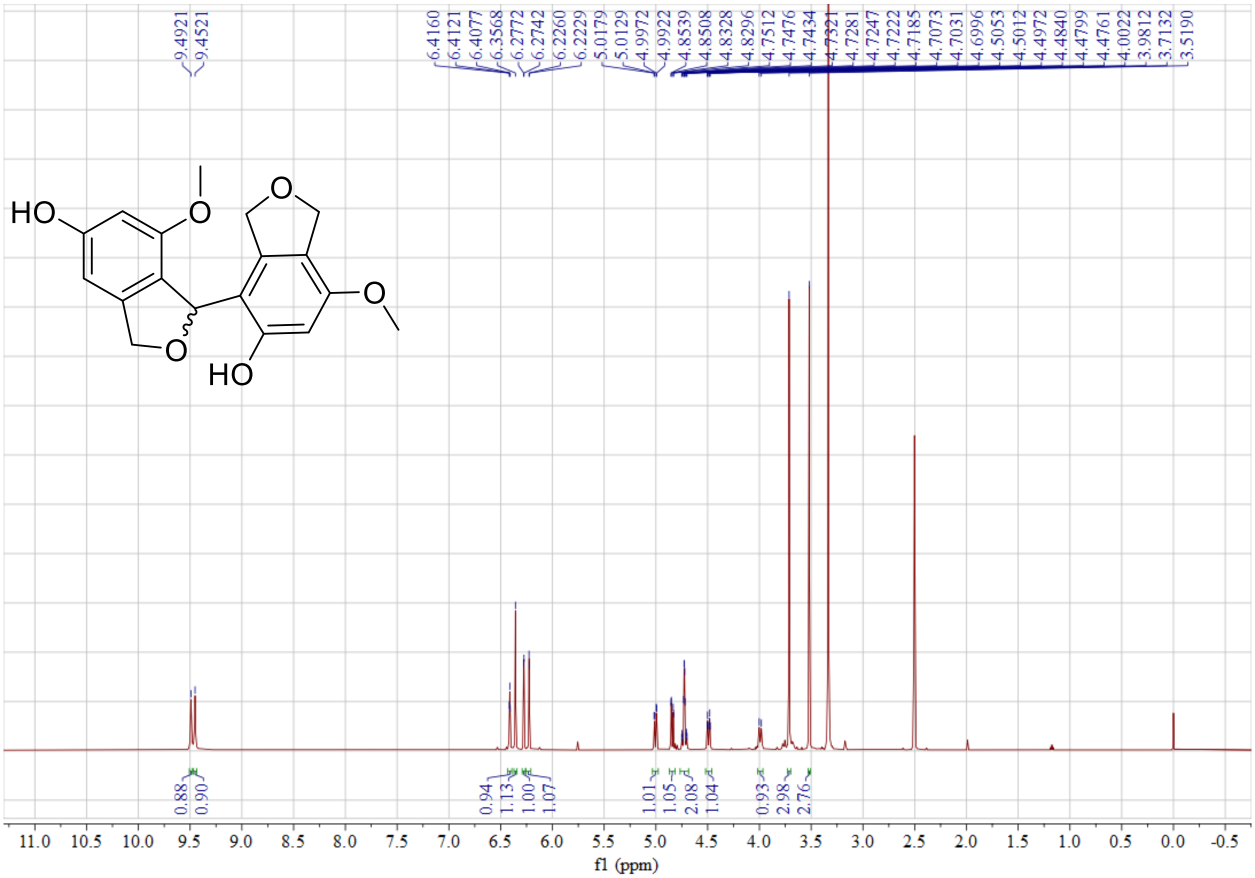

Figure S30. ^{1}H NMR spectrum of cymopolyphenol E (**5**) in DMSO-d_6 (600 MHz).

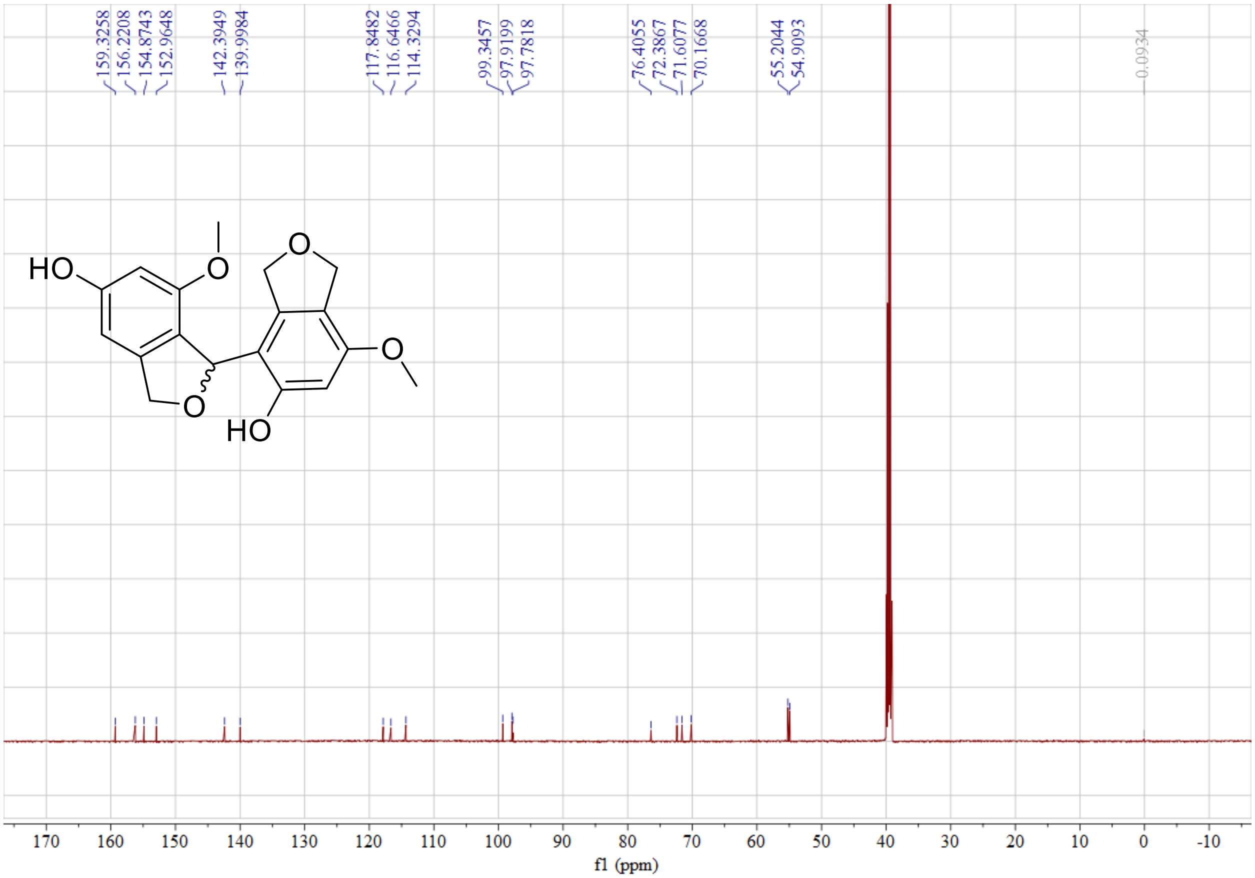

Figure S31. ^{13}C NMR spectrum of cymopolyphenol E (**5**) in DMSO-d_6 (150 MHz).

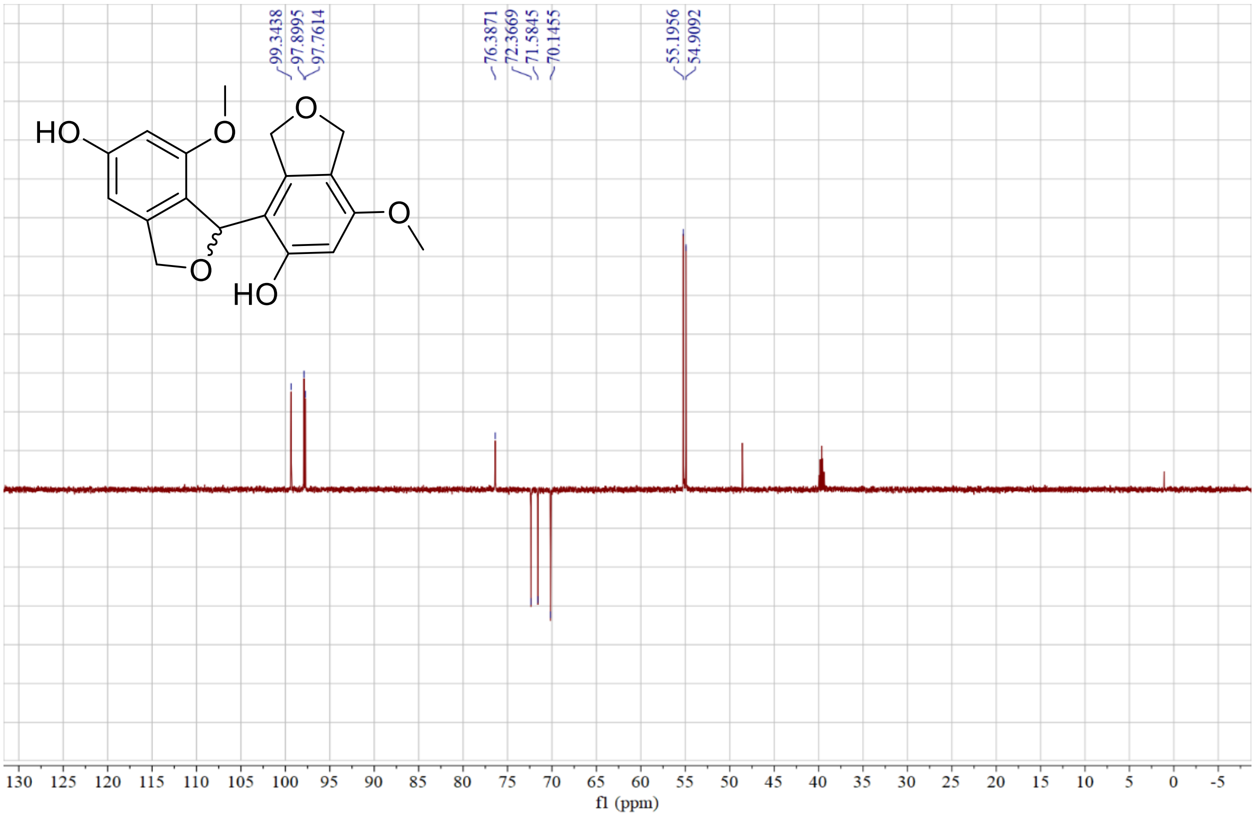

Figure S32. DEPT135 spectrum of cymopolyphenol E (**5**) in DMSO-d_6 (150 MHz).

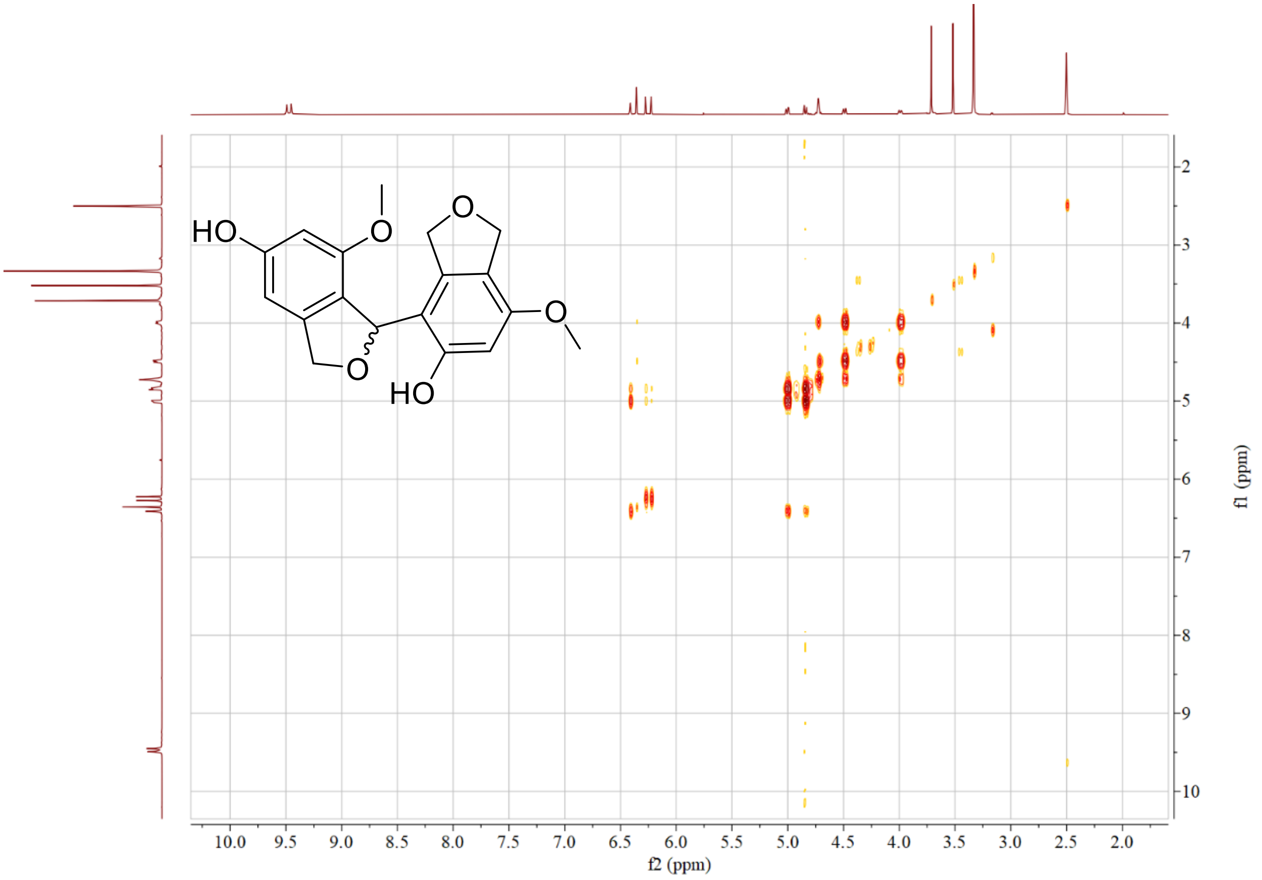

Figure S33. ^{1}H-^{1}H COSY spectrum of cymopolyphenol E (**5**) in DMSO-d_6.

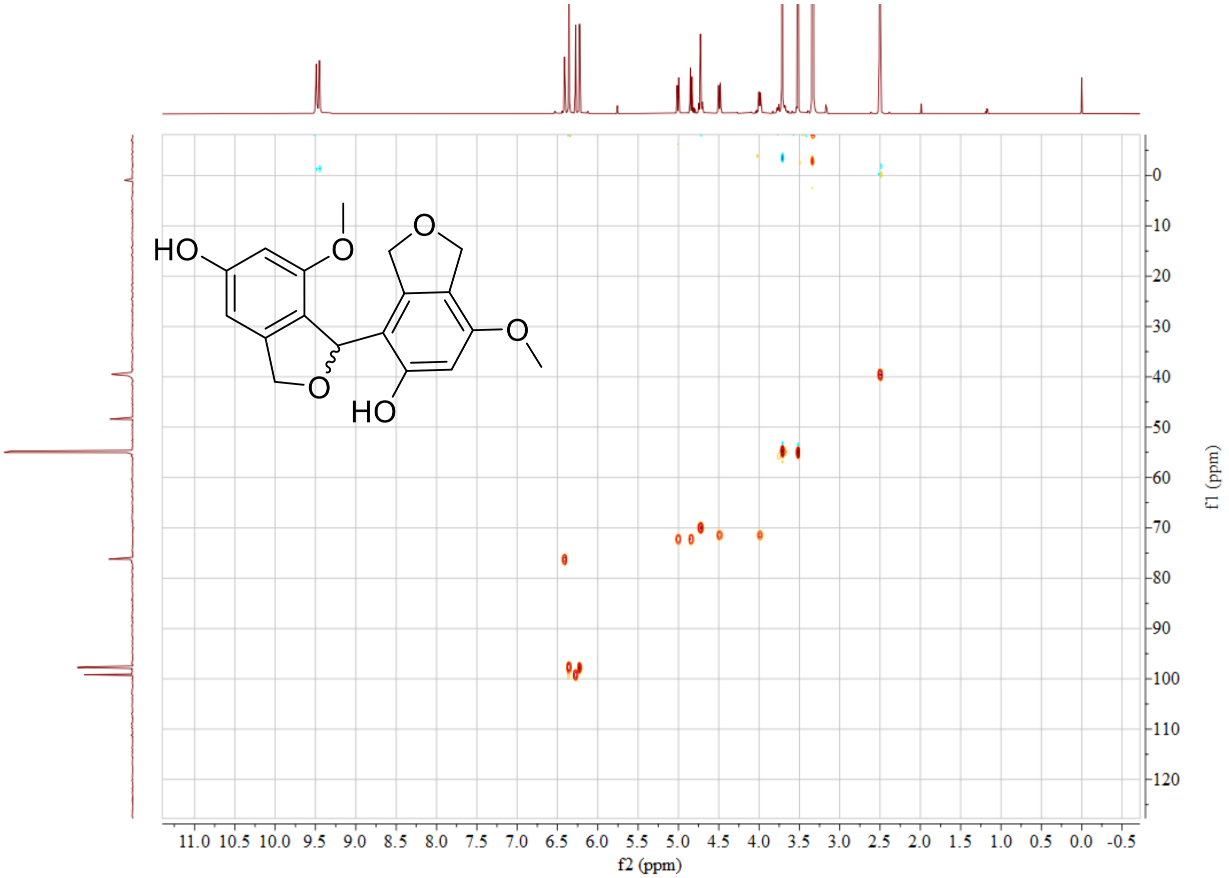

Figure S34. ^{1}H-^{13}C HSQC spectrum of cymopolyphenol E (**5**) in DMSO-d_6.

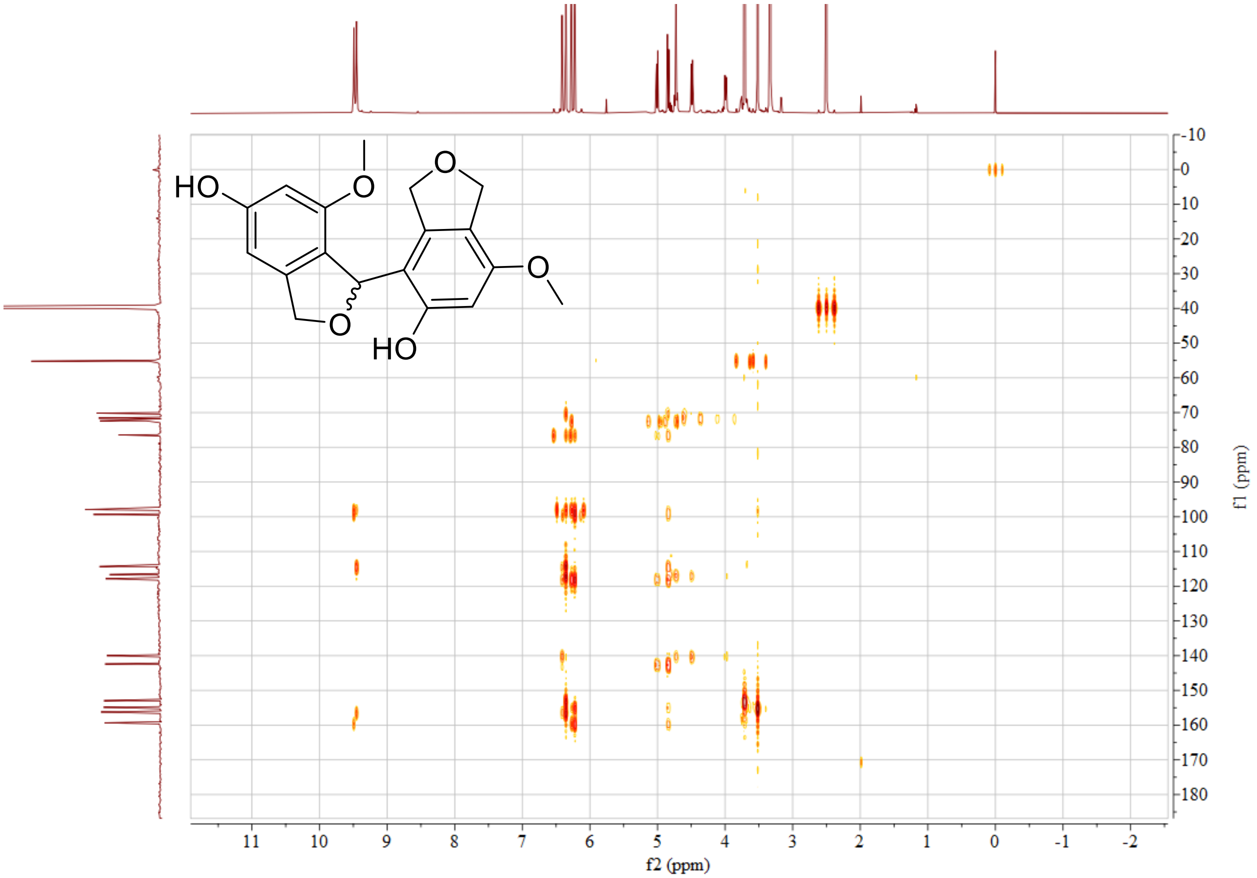

Figure S35. ^{1}H-^{13}C HMBC spectrum of cymopolyphenol E (**5**) in DMSO-d_6.

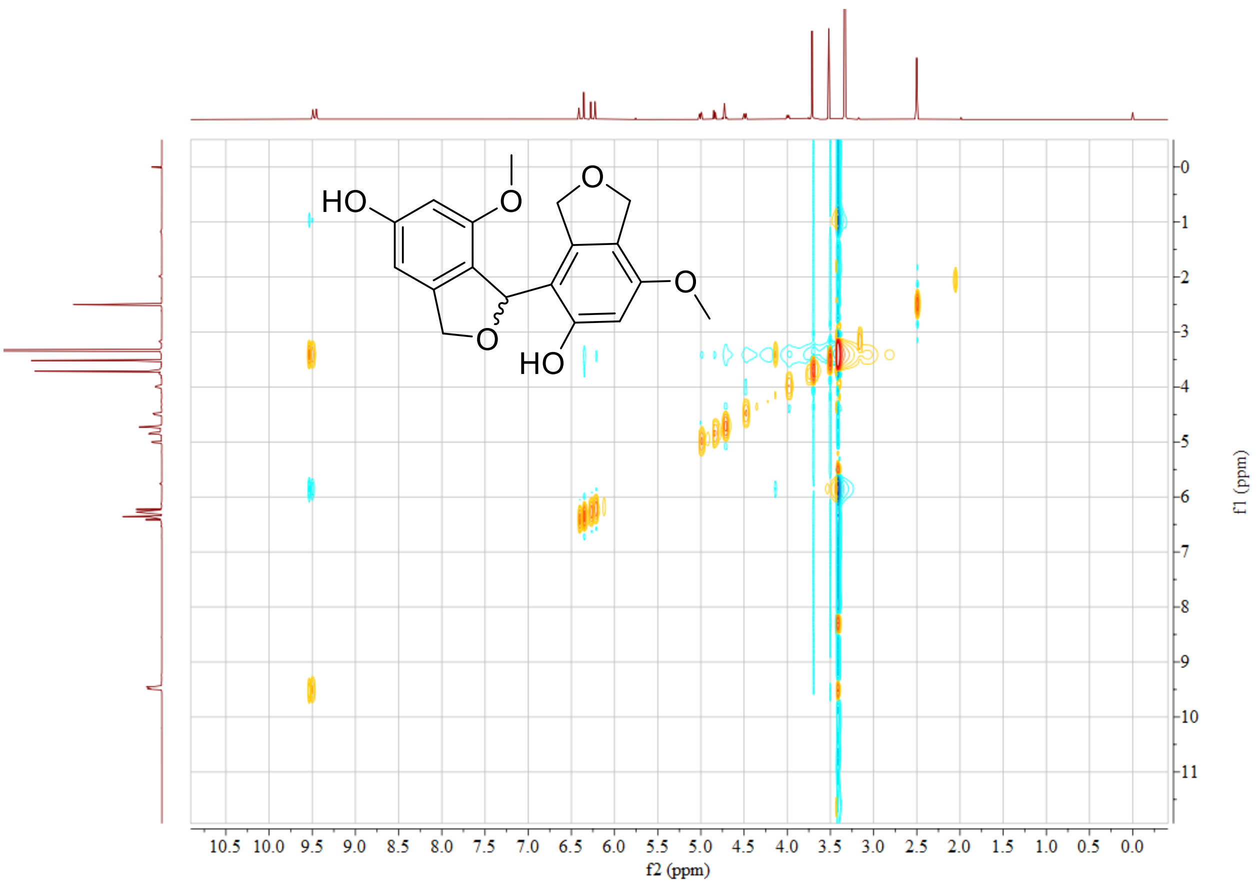

Figure S36. NOESY spectrum of cymopolyphenol E (**5**) in DMSO-*d*6.

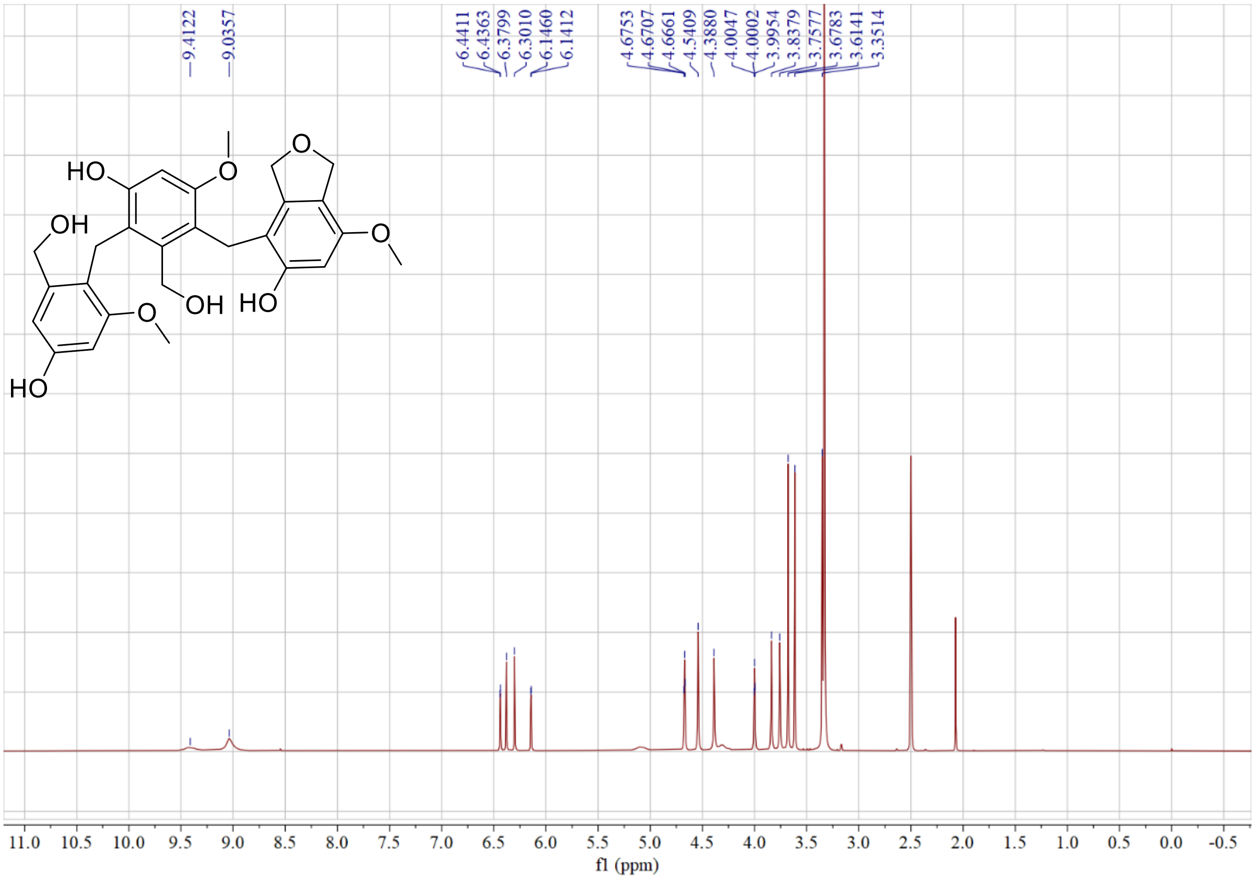

Figure S37. ^{1}H NMR spectrum of cymopolyphenol F (**6**) in DMSO-d_6 (500 MHz).

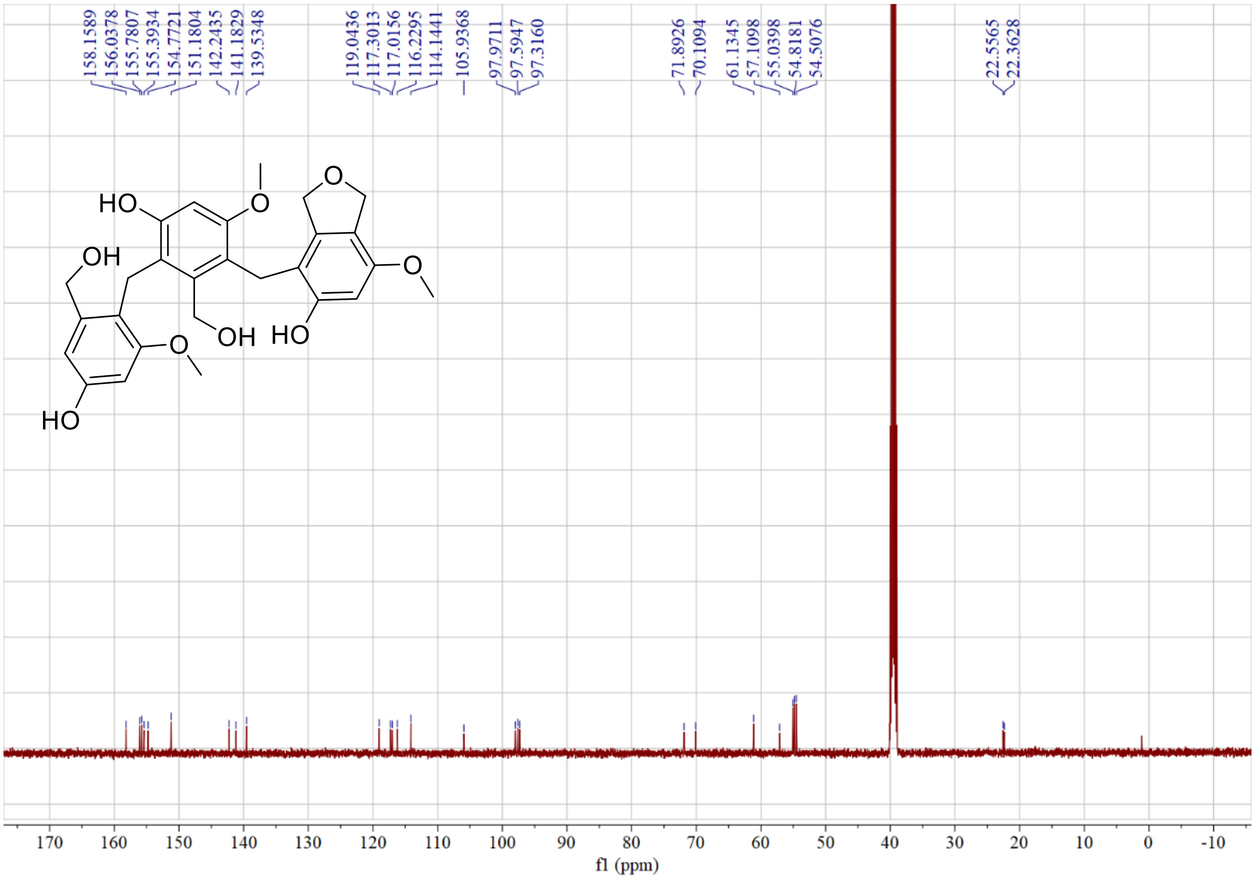

Figure S38. ^{13}C NMR spectrum of cymopolyphenol F (**6**) in DMSO-d_6 (125 MHz).

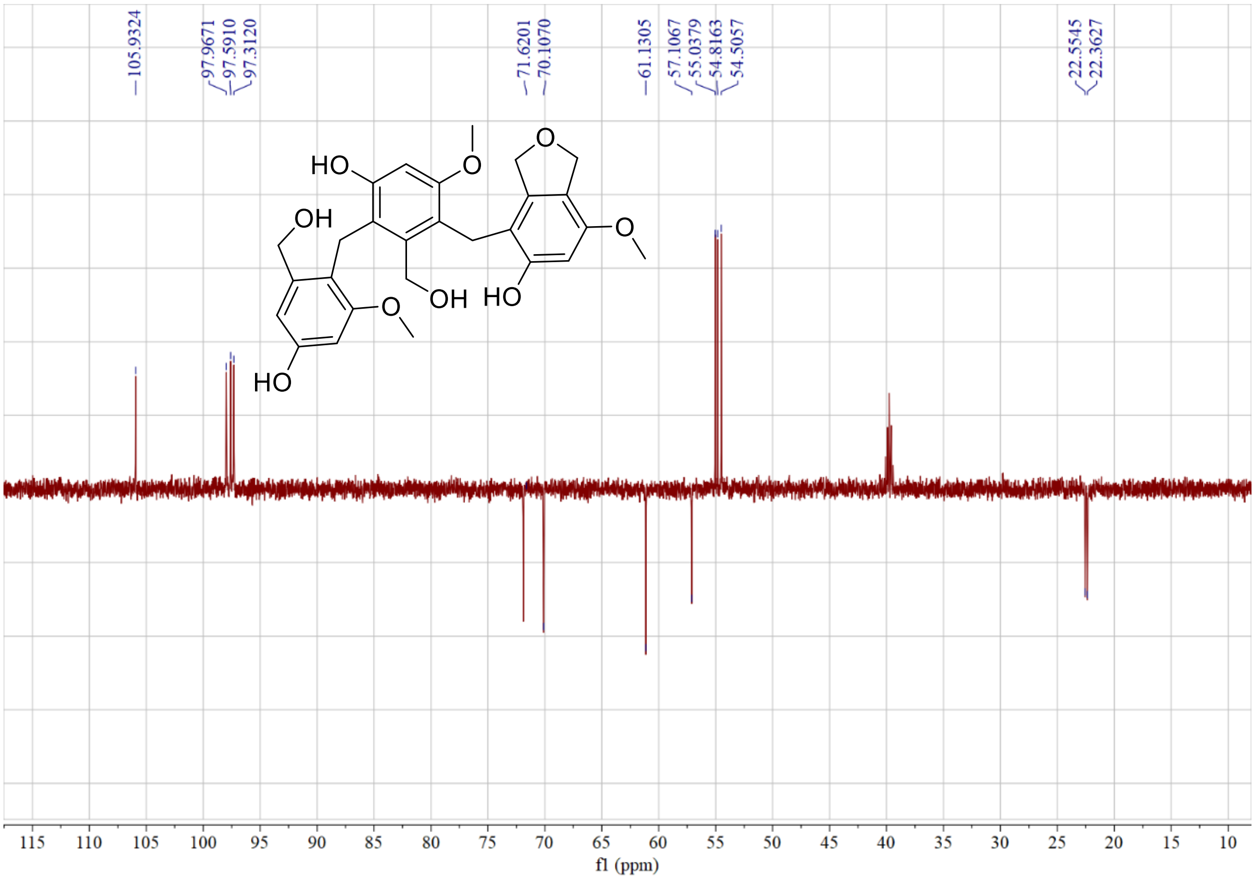

Figure S39. DEPT135 spectrum of cymopolyphenol F (**6**) in DMSO-d_6 (125 MHz).

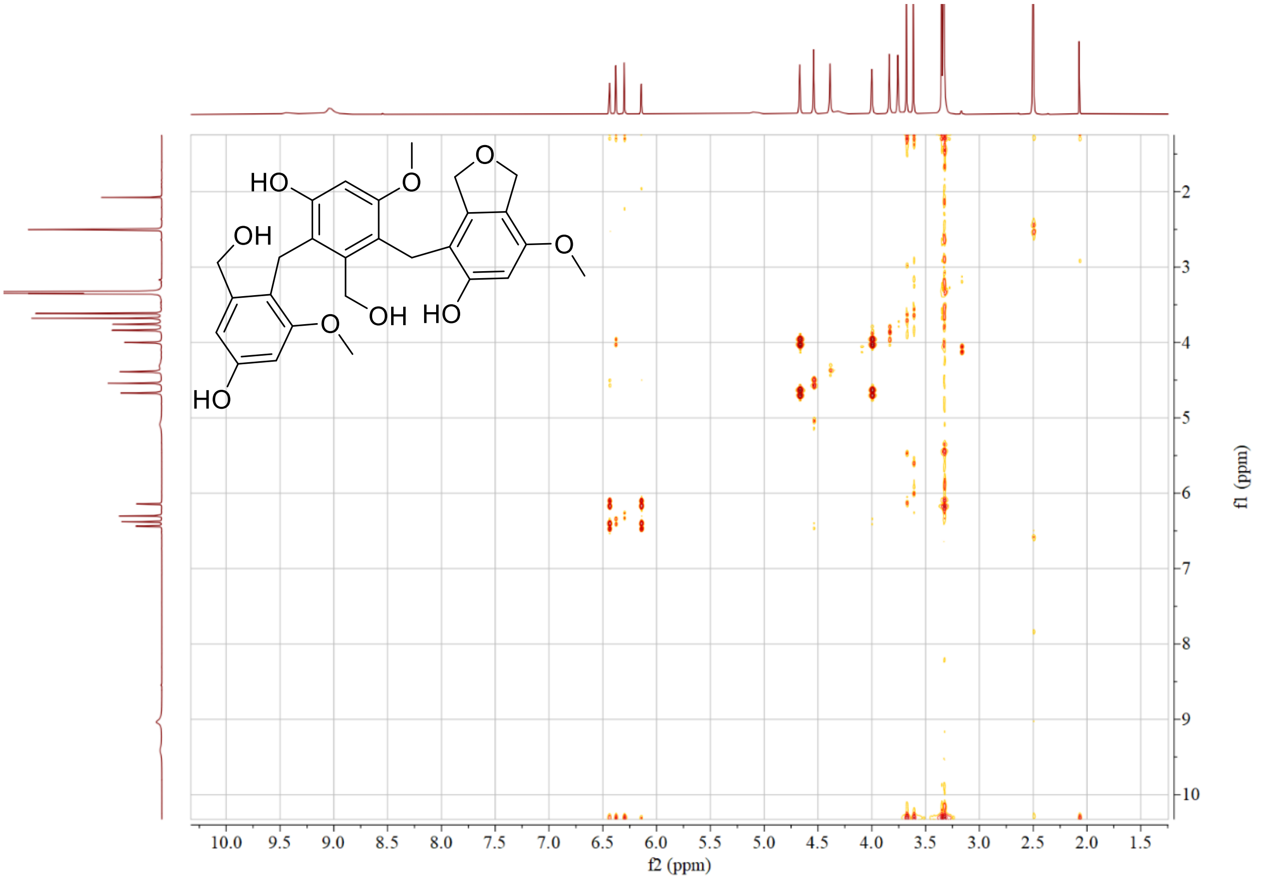

Figure S40. ^{1}H-^{1}H COSY spectrum of cymopolyphenol F (**6**) in DMSO-d_6.

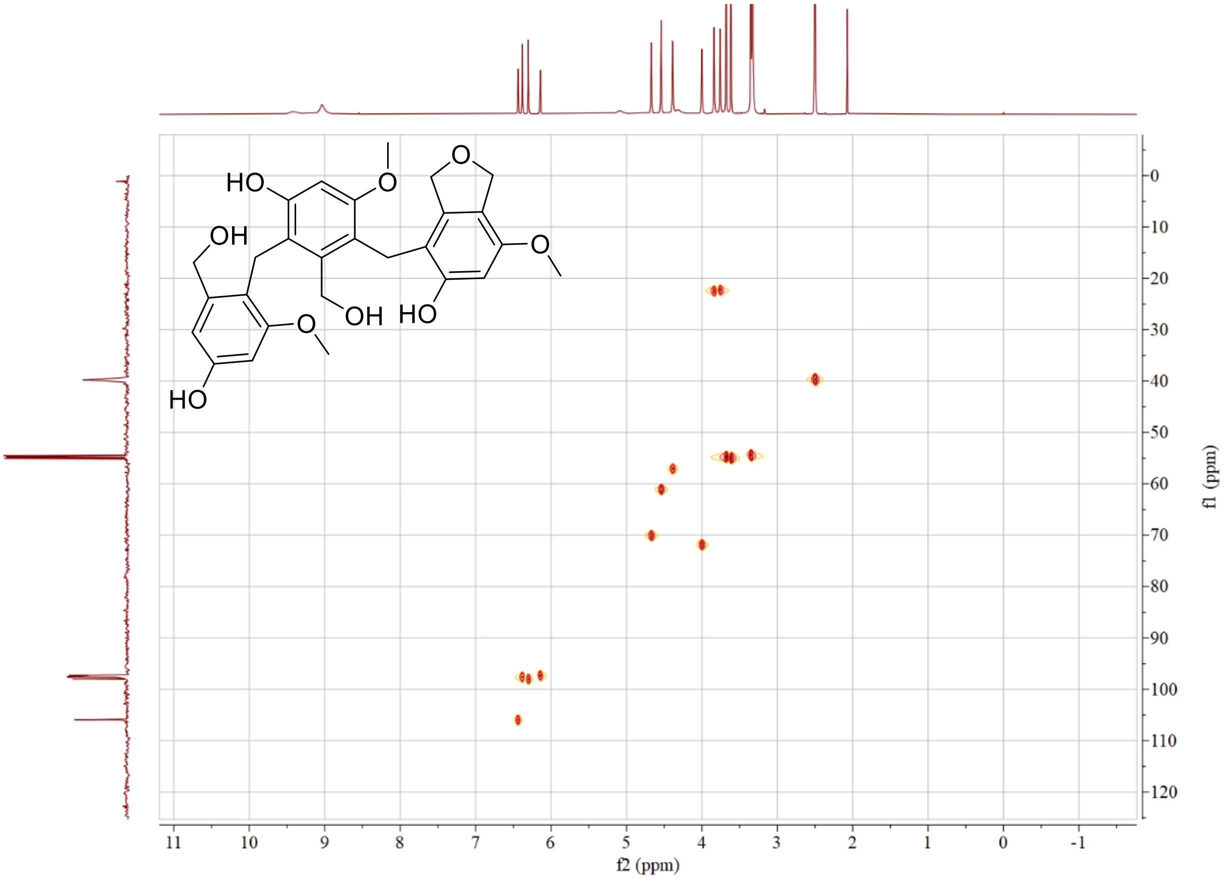

Figure S41. ^{1}H-^{13}C HSQC spectrum of cymopolyphenol F (**6**) in DMSO-d_6.

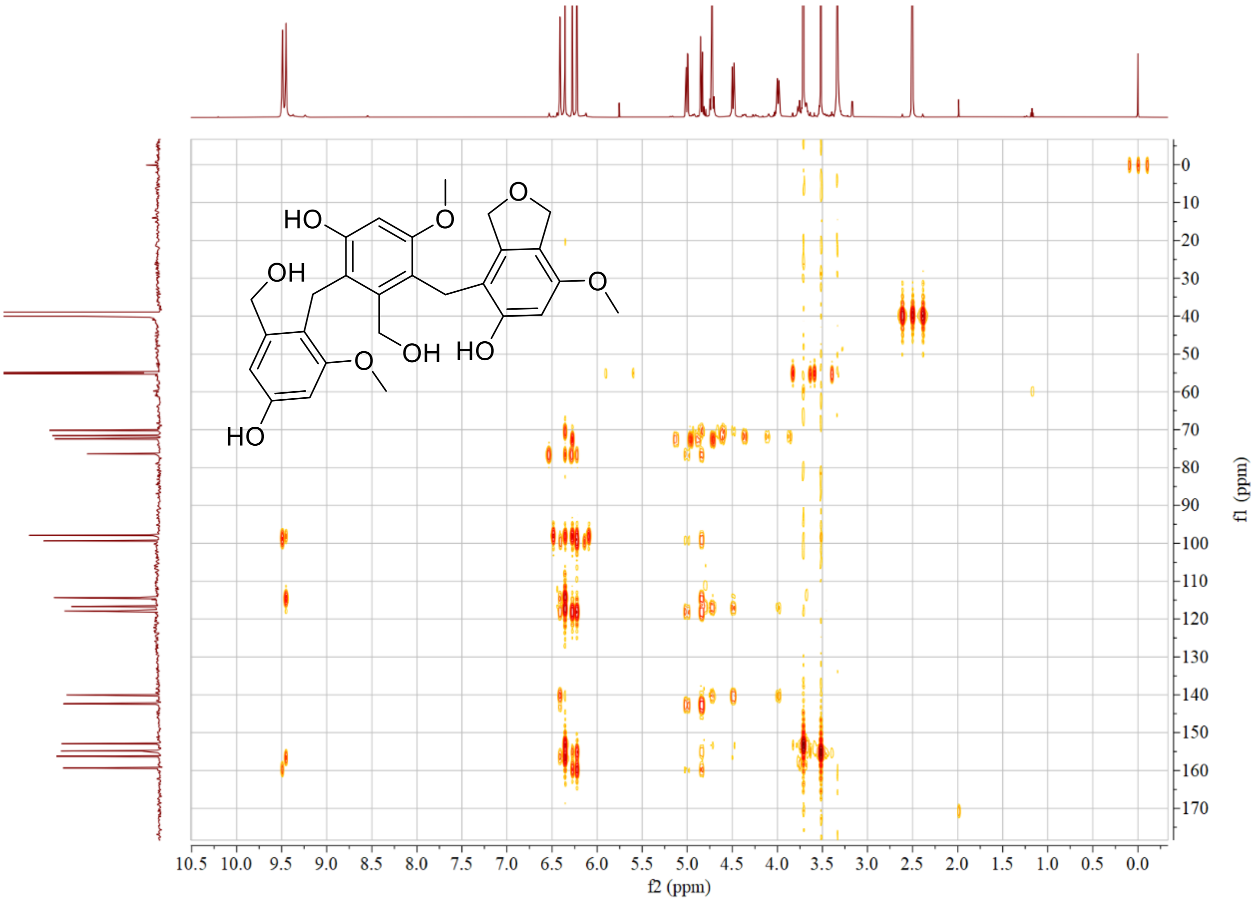

Figure S42. ^{1}H-^{13}C HMBC spectrum of cymopolyphenol F (**6**) in DMSO-*d*$_6$.

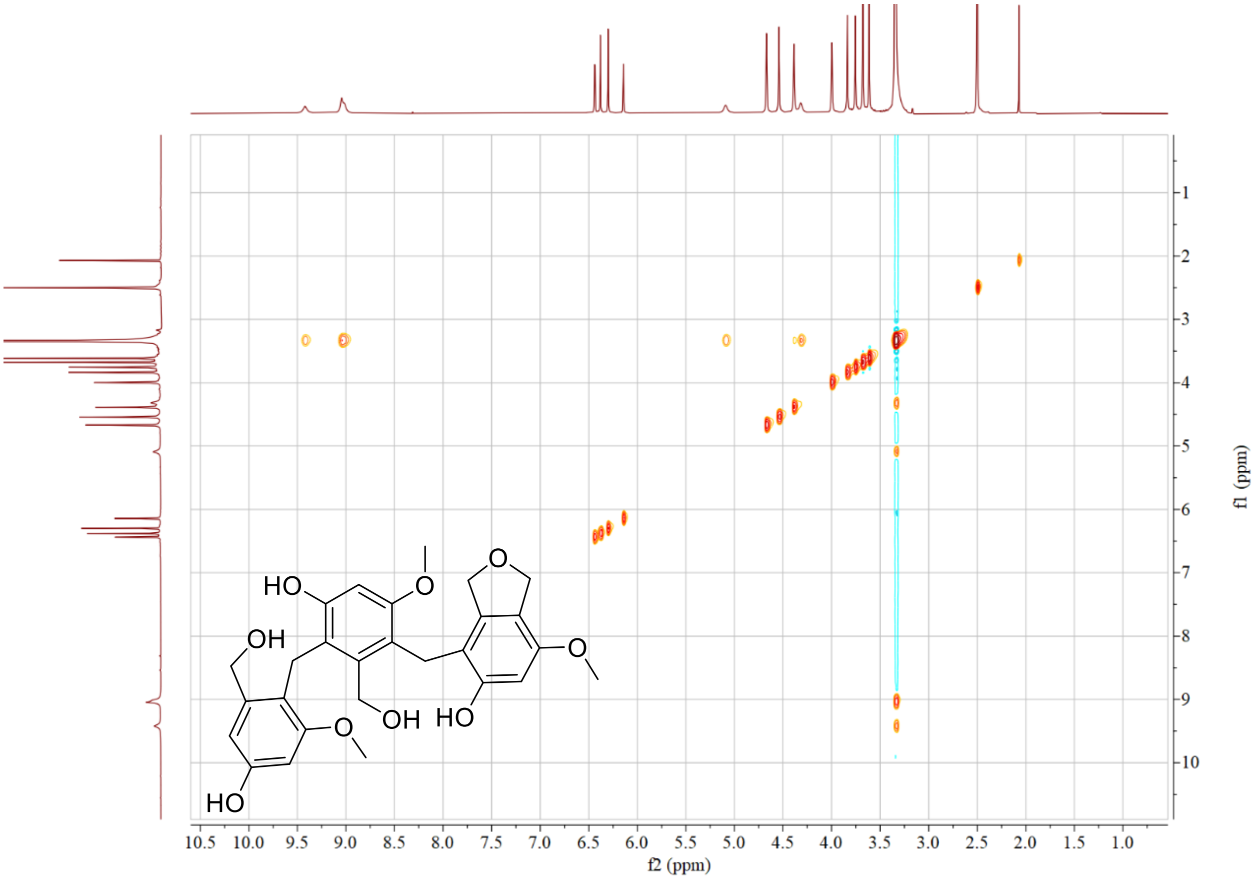

Figure S43. NOESY spectrum of cymopolyphenol F (**6**) in DMSO-d_6.
